
JOURNAL INFORMATION
==============================
NLM Title Abbreviation: Artery Res
Journal ID: Artery Res
NLM Journal ID: 101310251

Artery Research

ISSN: 1872-9312
EISSN: 1876-4401

ARTICLE INFORMATION
==============================
PMCID: PMC8703209
PMID: 34976230
DOI: 10.1007/s44200-021-00008-4
Article ID: 8
Article version: 1
Subjects: Abstracts

Selected Abstracts from Artery 21

Thursday 21 – Saturday 23 October 2021, Hôpital Européen Georges-Pompidou, Paris, France

Electronic publication date: 2021 Dec 24
Print publication date: 2021
Volume: 27
Issue: Suppl 1
First page: 1
Last page: 36
Copyright: © The Author(s) 2021
License: Open AccessThis article is licensed under a Creative Commons Attribution 4.0 International License, which permits use, sharing, adaptation, distribution and reproduction in any medium or format, as long as you give appropriate credit to the original author(s) and the source, provide a link to the Creative Commons licence, and indicate if changes were made. The images or other third party material in this article are included in the article's Creative Commons licence, unless indicated otherwise in a credit line to the material. If material is not included in the article's Creative Commons licence and your intended use is not permitted by statutory regulation or exceeds the permitted use, you will need to obtain permission directly from the copyright holder. To view a copy of this licence, visit http://creativecommons.org/licenses/by/4.0/.
License URL: https://creativecommons.org/licenses/by/4.0/

issue-copyright-statement: © The Author(s) 2021

==============================

*Artery Research* 2021, **27**(1): (2021) 27:S1–S36
https://doi.org/10.1007/s44200-021-00008-4

Artery Research

**ABSTRACTS** **Open Access**

### Selected Abstracts from Artery 21

Thursday 21 – Saturday 23 October 2021, Hôpital Européen Georges-Pompidou, Paris, France

Published online: 24 December 2021

All content was reviewed and approved by the Society Committee, which held full responsibility for the abstract selections.

### 1.1
### Central pulse pressure in adolescence is more strongly associated with future cardiovascular health than peripheral pulse pressure

**Dr Chloe Park**[1], Dr Siana Jones[1], Dr Hannah Taylor[1], Dr Laura Howe[2], Professor Abigail Fraser[2], Professor Nish Chaturvedi[1], Professor Alun Hughes[1]

[1]UCL, London, United Kingdom, [2]Univeristy of Bristol, Bristol, United Kingdom

**Background:** Increased left ventricle mass (LVM), arterial stiffness and carotid intima-media thickness (cIMT) are predictors of cardiovascular disease independent of blood pressure. Cross-sectional investigation in adolescence has shown that central pulse pressure (cPP) is more closely associated with target organ damage than peripheral pulse pressure (pPP) (1). Longitudinal follow-up of these adolescents is required to determine if pPP or cPP is more closely associated with future cardiovascular health.

**Methods:** 3898 participants (2173 female) in the Avon Longitudinal Study of Parents and Children (ALSPAC) underwent repeat measurements of pPP and cPP (SphygmoCor), LVM (echocardiography n = 1346), carotid-to-femoral pulse wave velocity (cfPWV n = 1596, Vicorder) and cIMT (n = 1520) aged 17 years and 24 years. LVM was indexed to height$^{1.7}$ (LVMI). Multivariable linear regression was used to assess longitudinal associations between pPP/cPP aged 17 years and LVMI, cfPWV and cIMT aged 24 years. Data for sexes were pooled and adjusted for age, sex and parental socioeconomic position (model 1). Bootstrapping (10,000 replications) was used to compare pPP and cPP associations.
**Results:** Aged 17 years, the difference between pPP and cPP was marked (mean difference (95% CI) = 20.7 (20.5, 21.0) mmHg). pPP and cPP were both positively associated with future LVMI cfPWV and cIMT aged 24 years (Table 1) but associations were stronger for cPP (bootstrap $p < 0.0001$ for all). Differences in strength of associations remained after adjustment (model 1).

Table 1: Associations between cPP and pPP aged 17yrs and future LVMI, cfPWV and cIMT aged 24yrs
(Data are beta coefficients (ß) from regression analysis and 95% confidence intervals (CI))

| | Unadjusted | | Model 1 Adjusted for age, sex and socioeconomic position | |
|---|---|---|---|---|
| **LVMI (g/m$^{1.7}$)** | ß (95 CI) | p-value | ß (95 CI) | p-value |
| cPP (mmHg) | 0.29 (0.24,0.35) | <0.0001 | 0.40 (0.29,0.51) | <0.0001 |
| pPP (mmHg) | 0.18 (0.15,0.22) | <0.0001 | 0.26 (0.18,0.34) | <0.0001 |
| **cfPWV (m/s)** | | | | |
| cPP (mmHg) | 0.034 (0.026,0.048) | <0.0001 | 0.009 (-0.002,0.019) | 0.1 |
| pPP (mmHg) | 0.020 (0.014,0.026) | <0.0001 | 0.002(-0.005,0.008) | 0.6 |
| **cIMT (mm)** | | | | |
| cPP (mmHg) | 0.0012 (0.0009,0.0017) | <0.0001 | 0.0012 (0.0008,0.0017) | <0.0001 |
| pPP (mmHg) | 0.0009 (0.0006,0.0011) | <0.0001 | 0.0009 (0.0006,0.001) | <0.0001 |

**Conclusion:** Adolescent cPP is more closely associated with future LVM, cfPWV and cIMT than bPP. These results suggest that central rather than peripheral blood pressure may be a better measure of future risk in adolescence.

### 1.2
### New carotid stiffness population centiles in the young and association with measures of general and abdominal obesity

**Mrs. Julia Charlotte Büschges**[1,2], Angelika Schaffrath Rosario[1], Dr. Giselle Sarganas[1,2], Dr. Anja Schienkiewitz[1], Dr. Karsten Königstein[3], Dr. Arno Schmidt-Trucksäss[3], Dr. Hannelore Neuhauser[1,2]

[1]UCL, London, United Kingdom, [2]Univeristy of Bristol, Bristol, United Kingdom, [1]Department of Epidemiology and Health Monitoring, Robert Koch-Institute,, Germany, [2]DZHK (German Centre for Cardiovascular Research), Germany, [3]Department of Sport, Exercise and Health, Division Sports and Exercise Medicine, University of Basel, Basel, Siwtzerland

**Background:** Data on carotid stiffness (cS) in unselected adolescents and young adults are scarce. To validly investigate associations with established risk factors, centiles are needed by age and growth. Although evidence has accumulated linking obesity in the young to elevated cIMT, studies rarely included cS or compare obesity parameters.

**Methods:** The KiGGS cohort 11-year-follow-up included high-resolution B-mode CCA-sonography with semi-automated edge-detection and automatic electrocardiogram-gated real-time quality control. Distensibility coefficient, stiffness index ß, Young's and Peterson's elastic modulus were assessed in 4,305 participants aged 14–28 years. Following cS and CIMT centile estimation with GAMLSS models, associations were investigated using log-binominal regression models with cS and CIMT ≥ 90th centile as outcomes and covariates including

© The Author(s) 2021, corrected publication2022. **Open Access** This article is licensed under a Creative Commons Attribution 4.0 International License, which permits use, sharing, adaptation, distribution and reproduction in any medium or format, as long as you give appropriate credit to the original author(s) and the source, provide a link to the Creative Commons licence, and indicate if changes were made. The images or other third party material in this article are included in the article's Creative Commons licence, unless indicated otherwise in a credit line to the material. If material is not included in the article's Creative Commons licence and your intended use is not permitted by statutory regulation or exceeds the permitted use, you will need to obtain permission directly from the copyright holder. To view a copy of this licence, visit http://creativecommons.org/licenses/by/4.0/.

obesity measures (BMI, waist circumference WC, waist-to- height ratio, fat mass and fat free mass) and a risk score from added z-scores of triglycerides, total/HDL-cholesterol-ratio and HbA1c.
**Results:** Multivariable models show a pattern of associations of obesity measures with various cS parameters with mostly moderate effect estimates, but consistent direction (relative risks between 1 and 2). This holds for longitudinal analyses and cross-sectional analyses, e.g. RR of 1.72 (CI 1.22–2.42) for baseline BMI ≥ P90 on elevated ß stiffness index. Associations with elevated CIMT are pointing in the same direction, but are mostly not statistically significant.
**Conclusions:** Using third-generation sonography and centiles computed with novel statistical methods, this study confirms that adiposity in childhood is linked to subclinical atherosclerosis in young adulthood. The results emphasize the importance of interventions in adolescence to delay the development of vascular alterations.

### 1.3
### Does sex and calibration influence cardiovascular risk prediction from central systolic blood pressure?

**Dr Florence Lamarche**[1], Dr Mohsen Agharazii[3], Dr Siegfried Wassertheurer[4], Dr Bernhard Hametner[4], Dr Annie-Claire Nadeau-Fredette[2], Dr François Madore[1], Dr Remi Goupil[1]
[1]Hopital de Sacré-coeur de Montréal, [2]Hôpital Maisonneuve-Rosemont, [3]CHU de Québec, [4]Austrian Institute of Technology
**Background:** The accuracy of central BP is usually improved from calibration with MAP and DBP (C2SBP) compared to calibration with SBP and DBP (C1SBP). While preliminary data suggest C2SBP may have the best accuracy in females, we aimed to assess whether this could translate into improved cardiovascular (CV) risk prediction.

**Methods:** 12,927 participants free of baseline CV disease, with prospective follow-up from administrative databases and central BP measurements were included. C1SBP was estimated with SphygmoCor Px devices. C2SBP was derived from unprocessed radial pressure waveforms recalibrated with DBP and 40% form factor derived MAP. Participants with heart rate < 60 were excluded due to incomplete waveforms. Major adverse CV events (MACE) comprised myocardial infarction, stroke, heart failure with hospitalization and CV death. Multivariable Cox regressions, differences in area under the curve, net reclassification index and integrated discrimination index were calculated comparing C2SBP to C1SBP and to bSBP, with and without stratification for sex.
**Results:** Over a median follow-up of 10.1 years (IQR 9.9–10.3), 2125 MACE (723/7013 females and 860/5934 males) occured. All BP parameters were significantly associated with MACE, regardless of sex. In the overall cohort, risk prediction metrics marginally favored C2SBP compared to bSBP, but were similar to C1SBP. No significant improvement of CV risk prediction was found in sex-stratified analyses (see Table).
**Conclusions:** C2SBP marginally improved CV risk prediction when compared to bSBP but not to C1SBP in the overall cohort only. All three BP parameters were similarly predictive in both sex, although this analysis possibly lacked power. This may be related to the FF-derived MAP (rather than oscillometric MAP), which is highly dependent on the brachial SBP.

Table 1. Central blood pressure calibration method and cardiovascular risk prediction

| Cohort | Calibration method | ΔAUC (95% CI) | NRI (95% CI) | IDI (95% CI) |
|---|---|---|---|---|
| All | C2SBP vs Brachial SBP model | 0.05 (-0.12, 0.12) | 0.11 (0.03, 0.17) | 0.0002 (-0.0001, 0.0007) |
| | C2SBP vs C1SBP model | -0.01 (-0.10, 0.07) | -0.01 (-0.10, 0.09) | -0.0004 (-0.0006, 0.0003) |
| Males | C2SBP vs Brachial SBP model | 0.05 (-0.05, 0.13) | 0.08 (-0.05, 0.14) | 0.0003 (-0.0001, 0.0010) |
| | C2SBP vs C1SBP model | 0.04 (-0.03, 0.11) | -0.03 (-0.11, 0.07) | -0.0001 (-0.0010, 0.0007) |
| Females | C2SBP vs Brachial SBP model | 0.06 (-0.06, 0.18) | 0.11 (-0.03, 0.19) | 0.0001 (-0.0004, 0.0010) |
| | C2SBP vs C1SBP model | 0.03 (-0.11, 0.17) | 0.03 (-0.11, 0.14) | -0.0000 (-0.0007, 0.0009) |

Each model includes the relevant BP parameter and age, BMI, smoking status, diabetes, HDL-c, total cholesterol, eGFR, heart rate and use of aspirin, statin, β-blockers, calcium channel blockers, diuretics and renin-angiotensin system blockers. C1SBP, central SBP calibrated on brachial SBP and DBP; C2SBP, central SBP calibrated on brachial SBP and MAP; ΔAUC, difference in area under the receiver operating characteristic curve; NRI, net reclassification index; IDI, integrated discrimination index.

### 1.4
### Ethnic variations in body composition may help to explain differences in arterial stiffness: a UK cross-sectional study in hypertension

**Dr Luca Faconti**[1], Mr Ryan McNally[1], Miss Bushra Farukh[1], Professor Phil Chowienczyk[1]
[1]King's College London, London, United Kingdom
**Background::** Ethnic disparities in arterial stiffness have been reported in Europe although it is unclear if these are independent of traditional cardiovascular risk factors including fat mass which a more accurate measure of the metabolic phenotype compared to body mass index (BMI). Here we test if the difference in body composition (rather than BMI) explains any difference in arterial stiffness in a bi-ethnic cohort of hypertensive individuals in the UK.

**Methods:** Anthropometric data, biochemistry (including aldosterone/renin ratio (ARR)), arterial stiffness (carotid femoral pulse wave velocity (cf-PWV)) and multi-frequency bioelectrical impedance analysis (BIA) were measured in subjects with hypertension free from cardiovascular disease.
**Results:** 177 black (55% male) and 142 white (71% male) subjects were recruited. Black individuals had higher blood pressure (mean ± SE) (152 ± 1.48/93 ± 0.95 vs 143 ± 1.17/89 ± 0.9) and higher prevalence of diabetes (13% vs 3%, P < 0.05). BMI was higher in black subjects compared to white (30.08 ± 0.34 kg/m$^2$ vs 28.81 ± 0.49 kg/m$^2$, P = 0.038) and BIA revealed a higher percentage of fat mass (30.18 ± 0.71% vs 27.26 ± 0.77% respectively, P = 0.006). Unadjusted cf-PWV was higher in black vs white individuals (10.21 ± 0.17 m/s vs 9.35 ± 0.16, P = 0.035) After adjustment for age, gender, blood pressure, heart rate, creatinine, ARR, prevalence of diabetes, dyslipidaemia, antihypertensive use and BMI, the difference persisted (P = 0.031). However, when fat mass was substituted for BMI, significance was lost.
**Conclusions:** Difference in body composition may help to explain ethnic differences in arterial stiffness in hypertensive subjects living in the Northern Hemisphere.

### 1.5
### Central-to-peripheral pulse amplification and stiffness gradient determine dicrotic wave: Mediation by triphasic flow fluctuation

**Prof. Junichiro Hashimoto**[1,2], Dr. Kaname Tagawa[1], Dr. Berend Westerhof[3], Prof. Sadayoshi Ito[2,4]
[1]Miyagi University of Education Medical Center, Sendai, Japan, [2]Tohoku University Graduate School of Medicine, Sendai, Japan, [3]Vrije Universiteit Amsterdam, Amsterdam, Netherlands, [4]Katta General Hospital, Shiroishi, Japan
**Purpose:** Blood pressure fluctuates during diastole creating a dicrotic wave, but the mechanistic origin remains poorly understood. We sought to investigate hemodynamic determinants of this pressure fluctuation, focusing particularly on its association with diastolic flow fluctuation.

**Methods:** Using tonometry and ultrasound, pulse waveforms were recorded on the femoral artery in 592 patients (age: 55 ± 14 years) to estimate the diastolic pressure fluctuation as a residual wave amplitude against the mono-exponential decay and the diastolic flow fluctuation as a bidirectional (forward and reverse) velocity pulse height.[1,2] The radial, carotid, and dorsal pedis waves were also recorded to measure the peripheral/aortic pulse pressure (PP) and pulse wave velocity (PWV) ratios.
**Results:** In the femoral waveforms, the beginning of diastole corresponded with the reverse velocity peak, after which the dicrotic notch appeared. The femoral pressure and flow fluctuations were mutually correlated in amplitude as indexed to the total pulse height ($r$ = 0.63), although the latter preceded the former. In multivariate-adjusted models, higher peripheral/aortic PP and PWV ratios independently correlated with greater pressure and flow fluctuation indices ($P$ < 0.001). Mediation analysis revealed that the relationship between PP and PWV ratios and the pressure fluctuation index was mediated by the flow fluctuation index (indirect/total effect ratio: 57 [95% confidence interval, 42–80]% and 54 [30–100]%, respectively).
**Conclusions:** These results suggest that central-to-peripheral PP amplification and stiffness gradients produce triphasic flow fluctuations and thereby generate dicrotic pressure waves. Diminished pressure and stiffness gradients caused by aortic stiffening may thus reduce diastolic runoff leading to ischemic organ damage.

### 1.6

### Differences in systolic-diastolic distensibility indicate carotid wall viscosity in healthy controls, patients with hypertension and type 2 diabetes

**Mr Alessandro Giudici**[1], Professor Carlo Palombo[2], Mrs Michaela Kozakova[3], Dr Carmela Morizzo[2], Professor Giuseppe Penno[3], Dr Giuli Jamagidze[4], Mr Daniele Della Latta[4,5], Professor Dante Chiappino[4], Professor J. Kennedy Cruickshank[6], Professor Ashraf W. Khir[1]
[1]Brunel Institute for Bioengineering, Brunel University London, Uxbridge, United Kingdom, [2]Department of Surgical, Medical, Molecular Pathology and Critical Area Medicine, University of Pisa, Pisa, Italy, [3]Department of Clinical and Experimental Medicine, University of Pisa, Pisa, Italy, [4]G. Pasquinucci Heart Hospital, G. Monasterio Foundation, Pisa, Italy, [5]TeraRecon, Durham, USA, [6]School of Life-Course/Nutritional Sciences, King's College, St. Thomas' & Guy's Hospitals, London, United Kingdom

**Background:** Soft tissues, as the arterial wall, exhibit viscoelastic behaviours (1). Consequently, pressure (*P*)-diameter (*D*) loops show hysteresis, i.e., different loading and unloading paths. Arterial distensibility (*Ds*), an established predictor of cardiovascular risk, is related to the slope of the *P-D* relationship and may, hence, differ between the systolic ejection ($_sDs$) and diastolic recoil ($_dDs$) due to wall viscosity. This study aimed to characterise differences between $_sDs$ and $_dDs$ in a cohort of healthy controls, and hypertensives and type-2 diabetic (T2DM) patients.

**Methods:** *P* and *D* waveforms were acquired simultaneously at the left and right common carotid artery, respectively, in n = 35 controls, n = 113 T2DMs, and n = 41 hypertensives. $_sDs$ and $_dDs$ were estimated via a linear regression of the $P\text{-}D^2$ relationship in the range of end diastolic (DBP)-dicrotic notch pressures in the respective arms of the loop: where $D_d$ is the diameter at DBP. Wall viscosity was evaluated as the hysteresis area ($H_A$) enclosed in the *PD*-loop (2).

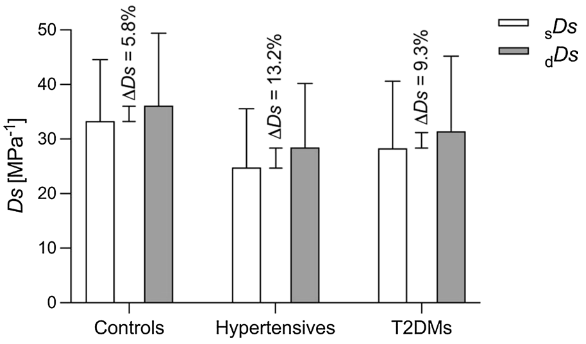

**Results:** $_dDs$ was 36.1 ± 13.2 $MPa^{-1}$ in controls, not significantly different from either hypertensives (28.3 ± 11.7 $MPa^{-1}$) or T2DMs (31.4 ± 13.8 $MPa^{-1}$). However, while $Ds = (_sDs - _dDs)/_dDs$ was only 5.8% in controls, it was higher at 9.3% (p = 0.12) and 13.2% (p = 0.007) in T2DMs and hypertensives, respectively (Figure). Similarly, $H_A$ was much higher in hypertensives at 0.96 ± 0.85 mmHg mm than controls (0.32 ± 0.34 mmHg mm, p < 0.001), with T2DMs exhibiting intermediate values (0.63 ± 0.63 mmHg mm).

**Conclusion:** Increasing distensibility differences between early systole and late diastole are due to elevated viscous properties of the carotid wall in hypertension.

### 1.7

### Acute effect of heat-not-burn versus standard cigarette smoking on arterial stiffness and wave reflections in young smokers

Research Associate Eleni Emmanouil[1], MD Nikolaos Ioakeimidis[1], MD Dimitrios Terentes-Printzios[1], MD Ioanna Dima[1], MD Konstantinos Aznaouridis[1], Professor Dimitris Tousoulis[1], Professor Charalambos Vlachopoulos[1]
[1]Hypertension and Cardiometabolic Syndrome Unit and Smoking Cessation Unit, 1st Cardiology Department, Athens Medical School, Hippokration Hospital, Athens, Greece

**Purpose:** The aim of this study was to compare the acute effects of heat-not-burn cigarette (HNBC) and standard tobacco cigarette (TC) smoking on arterial stiffness, assessed by carotid-femoral pulse wave velocity (c-fPWV), brachial-ankle (b-a) PWV and augmentation index (AIx).

**Methods:** We studied 22 current smokers (age: 33 ± 5 years, 55% females, no other risk factors, or any medications) on three different smoking sessions: a) HNBC heat stick, b) standard TC and c) sham cigarette. The mean nicotine content for both cigarette forms was 0.5 mg. Heart rate (HR), blood pressure (BP), AIx corrected for HR (AIx@75), c-fPWV and b-aPWV were assessed immediately before and after smoking, and then at 5, 10, 20 and 30 min.

**Results:** Both brachial and aortic systolic BP increased immediately after the end of TC smoking (by 11.5 and 10.5 mmHg, P < 0.001 and P < 0.01, respectively) and HNBC use (by 7.5 and 6 mmHg, all P < 0.01). Responses from baseline between the two smoking forms were not statistically significant at any time point throughout the entire study period (all P > 0.05). Compared to sham smoking, cfPWV, baPWV and AIx@75 increased immediately after the end of TC smoking (by 0.29 m/s, 93 cm/s and 3.3%, respectively) and remained increased after 5 min. Likewise, HNBC smoking induced a significant increase in cfPWV, baPWV, AIx@75 (by 0.30 m/s, 86 cm/s and 3.5%, respectively). (Figure).

**Conclusion:** TC and HNBC smoking acutely increased BP and arterial stiffness in young smokers, which is likely mediated, at least in part, by nicotine. This similar effect is questioning the characterization of HNBC smoking as a risk-reduction product, at least in the short-term. (1,2).

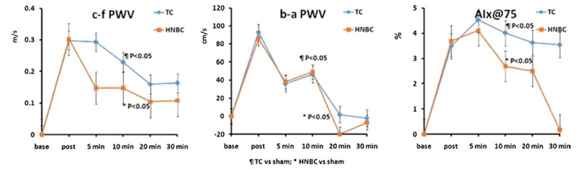

### 2.1

### Prediction of long-term outcomes by arterial stiffness and pressure wave reflections in patients with acute stroke: the Athens Stroke Registry

**Mrs Stamatia Samara**[1,2], Mrs Anastasia Vemmou[3], Mrs Aikaterini Kyrkou[3], Mr Christos Papamichael[3], Dr Eleni Korompoki[3], Dr George Ntaios[4], Dr Efstathios Manios[3], Dr Kimon Stamatelopoulos[3], Dr Athanasios Protogerou[2], Dr Konstantinos Vemmos[3]
[1]Laiko General Hospital, Athens, Greece, [2]Cardiovascular Prevention & Research Unit, Clinic & Laboratory of Pathophysiology, Department of Medicine, National and Kapodistrian University of Athens, Athens, Greece, [3]Therapeutic Clinic, Department of Medicine, National and Kapodistrian University of Athens, Athens, Greece, [4]Department of Internal Medicine, Faculty of Medicine, School of Health Sciences, University of Thessaly, Larissa, Greece, Larissa, Greece

**Background:** Stroke patients' management might be improved by addressing the role of aortic stiffness (carotid-femoral Pulse Wave Velocity—cfPWV) and pressure wave reflections (PWRs, augmentation index—AIx) in their pathogenesis and outcome[1].

**Methods:** We tested the hypothesis that cfPWV and AIx, separately and combined[2], predict long-term outcomes [all-cause mortality, incidence of cardiovascular events, stroke recurrence and disability defined by modified Ranking Scale (mRS) ≥ 3] in patients with acute stroke, using data from the "Athens Stroke Registry". We analyzed data from 552 patients (70% men, mean age: 66.1 10.4 years, mean follow-up 68.4 41.4 months, 13.4% deaths from any cause, 21.2% cardiovascular events, 14.1% stroke recurrences and 20.1% poor mRS).

**Results:** (a) high aortic stiffness (cfPWV > 13 m/sec) alone is an independent predictor of all-cause mortality and CV events, but not of stroke recurrence and poor functional outcome; (b) evaluated separately form aortic stiffness, neither low nor high PWRs have any prognostic value; (c) even after multiple adjustments, patients with both high aortic stiffness (cfPWV > 13 m/sec) and low PWRs (AIx < 22%) have almost twofold higher odds ratio, not only for all-cause mortality and CV events but also for stroke recurrence and poor functional outcome.

**Conclusions:** In the latter subgroup of stroke patients, increased cerebrovascular morbidity could be attributed to excessive pressure pulsatility in brain microcirculation, as a consequence of increased arterial stiffness combined with abnormally very low PWRs generated proximally to the microcirculation, resulting in loss of pulsatility buffering[3].

### 2.2
### Respective roles of hemodynamic conditions and inflammatory status in the degradation of endothelial glycocalyx in adults.

**Phd Jeremy Lagrange**[1], PharmD PhD Simon Toupance[1], Arthur Thomas[1], PhD Carlos Labat[1], PhD Véronique Regnault[1], MD PhD Athanase Benetos[1], MD PhD Patrick Lacolley[1]
[1]INSERM 1116, Vandoeuvre-lès-Nancy, France

**Background and objectives:** Interactions between blood components and the vascular wall involve the glycocalyx, a thin layer of carbohydrates covering endothelial cells. Glycocalyx is implicated in processes such as circulating cells adhesion, inflammation, and coagulation regulation and can be damaged in some pathologies. The prevailing hypothesis is that hypertension is the primary factor involved in the glycocalyx degradation. However, our preliminary results challenge this view and point to a more important role of inflammation. The objective of this study was to assess the respective roles of inflammation and hemodynamic on the endothelial glycocalyx degradation.

**Methods and results:** Plasma concentrations of syndecan-1 and thrombomodulin, two glycocalyx degradation markers, were quantified by ELISA in 327 atherosclerotic and non-atherosclerotic participants (62 ± 14 years) of the TELARTA cohort (telomere in arterial aging). Syndecan-1 was positively associated with circulating IL-6 ($p < 0.001$), IL-8 ($p = 0.002$), and IL-10 concentrations ($p = 0.006$) and with adhesion molecules ICAM-1 and VCAM-1 (p < 001). By contrast, no relation was observed between glycocalyx degradation markers and the hemodynamic parameters (systolic, diastolic and pulse pressure, pulse wave velocity), thus confirming the major role of inflammatory status in the degradation of endothelial glycocalyx. Interestingly, subjects with higher plasma concentration of syndecan-1 (third tertile) were more prone to present clinical manifestation of atherosclerosis (65 *vs* 42%; $p < 0.001$) than those with lower concentration (first tertile).

**Conclusions:** Inflammatory status appears as the major responsible of endothelial glycocalyx degradation in atherosclerosis. Deciphering the processes involved in this degradation and the possible treatment to limit it would be of interest in the next future.

### 2.3
### Apha1A-adrenoceptor-induced increased calcium influx and prostanoids unbalance promote carotid artery dysfunction in senescence-accelerated (SAMP8) female mice

**Dr Tiago J. Costa**[1], M.S. Paula R. Barros[1], Dr Diego Ângelo Duarte[1], M.S. Júlio A. da Silva-Neto[1], Dr Renèe de Nazaré Oliveira- da-Silva[2], Dr Rosangela A. Santos-Eichler[2], Dr Eliana H. Akamine[2], Dr Francesc Jiménez-Altayó[3], Dr Ana Paula Dantas[4], Dr Rita C. Tostes[1]
[1]Department of Pharmacology, Ribeirão Preto Medical School, University of Sao Paulo,, Brazil, [2]Department of Pharmacology, Institute of Biological Science, University of Sao Paulo,, Brazil, [3]Departament de Farmacologia, de Terapèutica i de Toxicologia, Facultat de Medicina, Institut de Neurociències, Universitat Autònoma de Barcelona,, Spain, [4]Laboratory of Experimental Cardiology, August Pi i Sunyer Biomedical Research Institute (IDIBAPS), Hospital Clinic Cardiovascular Institute,, Spain

**The** contribution of sympathetic nervous system overactivation to aging-associated cardiovascular dysfunction in postmenopausal women is controversial[1]. We tested the hypothesis that the alpha$_{1A}$ ($\alpha_{1A}$) adrenoceptor, an α-adrenergic receptor subtype, regulates calcium ($Ca^{2+}$) influx and prostanoids release, contributing to common carotid artery dysfunction in female senescence-accelerated mice. Eight-month-old senescence-accelerated (SAMP8) and control (SAMR1) mice were used to evaluate the carotid function. Binding assay with [3H] prazosin was used to determine the adrenergic receptor density. COX-1 protein expression was determined by western blot and prostanoids levels by ELISA. Results are presented as the mean ± SEM, n = 4–6. Statistical analysis: One-way ANOVA, followed by Bonferroni and $p < 0.05$. SAMP8 mice exhibited increased phenylephrine-induced vasoconstriction vs. SAMR1 [Rmax (% of 60 mM KCl-induced responses): 109 ± 3 vs. 81 ± 1, respectively)]. An α1A receptor antagonist, but not an α1D antagonist, abrogated the increased carotid contractions in SAMP8 (Rmax: 87 ± 4) vs. SAMR1 (89 ± 3) mice. The adrenergic receptor density and $Ca^{2+}$ influx were higher in SAMP8 arteries vs. SAMR1 arteries. An anti-α1A antibody reduced $Ca^{2+}$ influx in vascular smooth muscle cells of SAMP8. Prostacyclin ($PGI_2$), but not thromboxane$A_2$ ($TXA_2$) levels, and COX-1 protein expression were decreased in phenylephrine-stimulated carotids of SAMP8 vs. SAMR1 carotids. Our data suggest that the α1A adrenoreceptor subtype mediates common carotid artery dysfunction in aging by increasing $Ca^{2+}$ influx and promoting COX-1-mediated prostanoids unbalance.

Financial Support: FAPESP, Brazil (2017/25116–2).

### 2.4
### Vascular Ageing Glossary: unifying language for knowledge diffusion

Dr Peter Charlton[1,2], Dr Rachel Climie[3,4,5], Dr Christopher Clemens Mayer[6], Dr Manasi Nandi[7], Dr Arno Schmidt- Trucksäss[8], Dr Patrick Segers[9], **Dr Dimitrios Terentes-Printzios**[10], Dr Bianchini E. for VascAgeNet[11]
[1]The Department of Public Health and Primary Care, University of Cambridge, Cambridge, UK, [2]Research Centre for Biomedical Engineering, City, University of London, London, UK, [3]Menzies Institute for Medical Research, University of Tasmania, Hobart, Australia, [4]Baker Heart and Diabetes Institute, Melbourne, Australia, [5]Université de Paris, INSERM, U970, Paris Cardiovascular Research Center (PARCC), Paris, France, [6]AIT Austrian Institute of Technology, Center for Health & Bioresources, Vienna, Austria, [7]Faculty of life sciences and medicine, King's Collège London., London, UK, [8]MD. Division of Sports and Exercise Medicine, Department of Sport, Exercise and Health, Medical Faculty, University of Basel, Basel, Switzerland, [9]Institute for Biomedical Engineering and Technology (IBiTech), Ghent University, Ghent, Belgium,[10]First Department of Cardiology, Hippokration Hospital, Medical School, National and Kapodistrian University of Athens, Athens, Greece, [11]Institute of Clinical Physiology, CNR, Pisa, Italy

**Purpose:** VascAgeNet is a European multidisciplinary collaborative network on vascular ageing funded by the European Cooperation in Science and Technology (COST, CA18216). Multidisciplinary collaborations benefit from a common language and collective understanding of fundamental principles, concepts and techniques to strengthen communication among researchers within and outside the network and to improve readability of scientific outputs and their impact on the society [1].

**Methods:** The network agreed on the need for a vascular ageing glossary to promote a common language across the field. Experts from the network identified an initial list of terms and preliminary definitions. A dedicated team, including representatives from all Working Groups, was created to design the glossary development process, to facilitate its implementation [2] and to maximize outreach and dissemination.

**Results:** Regular meetings of the dedicated team were conducted, and main decisions were agreed by all members. The key steps of the process for managing the creation and maintenance of the glossary were to determine: (1) the target audience; (2) a list of priority terms; (3) a template structure for definitions; (4) methods for collecting expert feedback; and (5) the dissemination plan. An implementation strategy was provided by the team for each point (Figure). Small groups of terms will be released on a regular basis and published openly at https://vascagenet.eu/.

**Conclusions:** The strategy for the first Vascular Ageing Glossary has been successfully designed and developed within VascAgeNet. It will be a living document, available to the scientific community, which aims to unify the vascular ageing language.

### 2.5

### Acute and long-term effects of aortic banding on central hemodynamics

Stamatia Pagoulatou[1], Dionysios Adamopoulos[2], Vasiliki Bikia[1], **Georgios Rovas**[1], Nikolaos Stergiopulos[1]
[1]Laboratory of Hemodynamics and Cardiovascular Technology, Lausanne, Switzerland, [2]Department of Cardiology, Geneva University Hospitals, Geneva, Switzerland

**Background:** Aortic compliance is an important determinant of cardiac afterload and a contributor to cardiovascular morbidity. The aim of the present study was to provide in-silico insights into the acute as well as long-term effects of aortic compliance decrease on central hemodynamics by means of a 1D, validated mathematical model of the cardiovascular system.

**Methods:** Three hemodynamic states were simulated to represent a) a healthy young adult (baseline), b) acutely after banding of the proximal aorta, c) after the heart remodeled itself due to the increased afterload. The simulated pressure and flow waves were used for subsequent pulse wave and wave separation analysis.

**Results:** Aortic banding induced hypertension, which was sustained after LV remodeling. The main mechanism that drove hypertension was the enhancement of the forward wave, which became even more significant after LV remodeling (forward amplitude 30 mmHg at baseline vs 60 mmHg acutely after banding vs 64 mmHg after remodeling). Accordingly, the forward wave's contribution to the total pulse pressure increased throughout this process. Finally, LV remodeling was accompanied by a decrease in augmentation index (AIx 13% acutely after banding vs -3% after remodeling) and a change of the central pressure wave phenotype from the characteristic Type A ("old") to Type C ("young") phenotype (Fig. 1). Simulated pressure phenotypes were in close agreement with previous experimental findings (1).

**Conclusion:** These findings highlight the importance of the forward pressure wave in the development of hypertension and provoke us to reconsider our understanding of AIx as a solely arterial parameter.

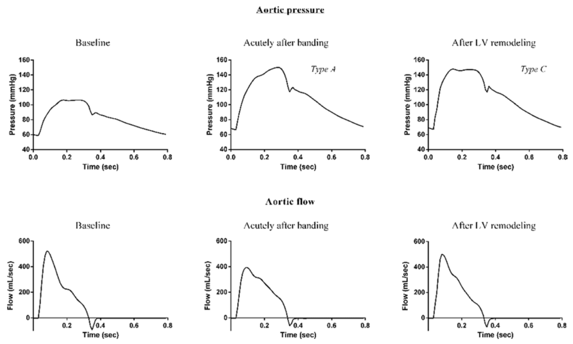

**Fig. 1** Acute and long-term changes of central aortic pressure and flow due to aortic banding. (*left*) Baseline. (*center*) Acutely after banding. (*right*) After LV remodeling under the form of concentric hypertrophy.

### 3.1

### The aortic-femoral arterial stiffness gradient is blood pressure independent in older adults: an atherosclerosis risk in communities (ARIC) study

**Miss Jillian Poles**[1], Mr. Keeron Stone[2], Dr. Simon Fryer[2], Dr. James Faulkner[3], Dr. Michelle Meyer[4], Dr. Kevin Heffernan[5], Dr. Anna Kucharska-Newton[6], Mr. Gabriel Zieff[1], Mr. Craig Paterson[2], Dr. Kunihiro Matsushita[7], Dr. Timothy Hughes[8], Dr. Hirofumi Tanaka[9], Dr. Lee Stoner[1]
[1]Department of Exercise and Sport Science, University Of North Carolina at Chapel Hill, Chapel Hill, United States, [2]School of Sport and Exercise, University of Gloucestershire, Gloucester, United Kingdom, [3]Department of Sport, Exercise & Health, University of Winchester, Winchester, United Kingdom, [4]Department of Emergency Medicine, School of Medicine, University of North Carolina at Chapel Hill, Chapel Hill, United States, [5]Department of Exercise Science, Syracuse University, Syracuse, United States, [6]Department of Epidemiology, The Gillings School of Global Public Health, University of North Carolina at Chapel Hill, Chapel Hill, United States, [7]Department of Epidemiology, Johns Hopkins Bloomberg School of Public Health, Baltimore, United States, [8]Section of Gerontology and Geriatric Medicine, Department of Internal Medicine, Wake Forest School of Medicine, Winston Salem, United States, [9]Department of Kinesiology and Health Education, The University of Texas at Austin, Austin, United States

**Background:** Aortic arterial stiffness is a strong independent predictor of cardiovascular disease (CVD), however its dependence on mean arterial pressure (MAP) limits its clinical utility. The aortic-femoral arterial stiffness gradient (af- SG), a novel marker of CVD risk, may be a promising alternative, but its dependence on MAP is not known. The aim of this study was to determine the relationship between MAP and the af-SG.

**Methods:** We evaluated the dependency of the af-SG on MAP in healthy older adults ($n = 694$, aged $74 \pm 5$ years), and adults with hypertension ($n = 2040$, aged $76 \pm 5$ years), and diabetes ($n = 1405$, aged $75 \pm 5$ years) as part of the community- based Atherosclerosis Risk in Communities (ARIC) Study. Carotid-femoral pulse-wave velocity (cfPWV), femoral-ankle PWV (faPWV), and blood pressure were measured using standardized protocols. The af-SG was calculated as faPWV divided by cfPWV. Multivariable regression analysis was performed to test the independent association of MAP with af-SG, with adjustments for known confounders including age, sex, body mass index, blood glucose and heart rate.

**Results:** There was no significant relationship between the af-SG and MAP in healthy ($\beta = 0.002$, $p = 0.301$), hypertension ($\beta = -\ 0.001$, $p = 0.298$) or diabetes ($\beta = -\ 0.001$, $p = 0.063$) population groups, with MAP explaining < 0.1, < 0.1 and 0.2% of the variance in the af-SG, respectively.

**Conclusions:** These findings suggest that the af-SG may be regarded as a MAP independent index of arterial health and CVD risk in older adults.

### 3.2

### Arterial stiffness is associated with impaired orthostatic diastolic blood pressure reaction and increased central blood pressure: A prospective population-based study

**Dr. Madeleine Johansson**[1], Prof. Peter M Nilsson[1], Prof. Gunnar Engström[1], Assoc Prof. Viktor Hamrefors[1]
[1]Lund University, Malmö, Sweden

**Background:** Arterial stiffness is independently associated with orthostatic hypotension. We aimed to investigate the relationship between orthostatic blood pressure reaction and arterial stiffness as well as central hemodynamics in younger subjects from the general population.

**Methods:** We analyzed a large prospective cohort study of 3756 individuals from the general population (mean age $41.9 \pm 14.5$ years, 52.1% women) in the Malmö Offspring Study (MOS), Sweden. Assessment of arterial stiffness and central hemodynamics was made by measurement of carotid-femoral pulse wave velocity (c-f PWV)

(Sphygmocor® AtCor, Australia) and pulse wave analysis (PWA) at the *arteria radialis* in relation to an orthostatic blood pressure reaction after 5 min standing using linear regression models.
**Results:** We found a significant association between orthostatic diastolic blood pressure reaction and c-f PWV levels, that is, increased c-f PWV as observed in arterial stiffness, was associated with lower diastolic blood pressure increase upon standing after adjusting for age and sex ($p=0.016$). Moreover, we observed that increased systolic blood pressure on standing was associated with a higher central blood pressure in the ascending aorta after adjusting for age, sex, hemoglobin $A_{1C}$ ($HbA_{1c}$) levels, and current smoking ($p=0.006$).
**Conclusions:** Our preliminary findings suggest that orthostatic hypotension, which is commonly observed in older individuals, is associated with markers of arterial stiffness (vascular ageing) and hemodynamic changes also in a younger population.

### 3.3
### The effect of mRNA vaccine against COVID-19 on endothelial function and arterial stiffness

**Dr. Dimitrios Terentes-Printzios**[1], Dr. Vasiliki Gardikioti[1], Dr. Eirini Solomou[1], Mrs Elena Emmanouil[1], Dr. Ioanna Gourgouli[1], Dr. Panagiotis Xydis[1], Mrs. Georgia Christopoulou[1], Dr. Christos Georgakopoulos[1], Dr Ioanna Dima[1], Mrs Antigoni Miliou[1], Dr. Georgios Lazaros[1], Dr. Maria Pirounaki[2], Prof. Konstantinos Tsioufis[1], Prof. Charalambos Vlachopoulos[1]
[1]First Department of Cardiology, Hippokration Hospital, Athens Medical, Athens, Greece, [2]Second Department of Medicine, University of Athens, Medical School, Hippokration General Hospital, Athens, Greece, Athens, Greece
**Purpose/background/objectives:** To fight the COVID-19 pandemic, messenger RNA (mRNA) vaccines were the first to be adopted by vaccination programs worldwide. We sought to investigate the short-term effect of mRNA vaccine administration on endothelial function and arterial stiffness.

**Methods:** Thirty-two participants (mean age $37 \pm 8$ years, 20 men) that received the BNT162b2 mRNA COVID-19 vaccine were studied in 3 sessions in a sequence-randomized, sham-controlled, assessor-blinded, cross-over design. Primary outcome was endothelial function assessed by brachial artery flow-mediated dilatation (FMD), and secondary outcomes were aortic stiffness, evaluated with carotid-femoral pulse wave velocity (PWV), and inflammation measured by high-sensitivity C- reactive protein (hsCRP) in blood samples. The outcomes were assessed prior to, and at 8 h, 24 h post the 1st dose of vaccination, and 8 h, 24 h and 48 h post the 2nd.
**Results:** There was an increase in hsCRP that was apparent at 24 h after both the 1st dose (− 0.60 [95% confidence intervals [CI] − 1.60 to − 0.20], $p=0.013$) and the 2nd dose (max median difference at 48 h − 6.60 [95% CI − 9.80 to − 3.40], $p<0.001$) compared to sham. The vaccine did not change PWV. FMD remained unchanged during the 1st dose but decreased significantly by 1.5% (95% CI 0.1–2.9%, $p=0.037$) at 24 h post the 2nd dose. FMD values returned towards baseline at 48 h.
**Conclusions:** Our study shows that the mRNA vaccine causes a prominent increase in inflammatory markers, especially after the 2nd dose and a transient deterioration of endothelial function at 24 h that returns towards baseline at 48 h. These results confirm the short-term cardiovascular safety of the vaccine.

### 3.4
### Early vascular ageing in patients with hypoparathyroidism

**Mr Saverio Fabbri**[1,2], Mr Pierre Boutouyrie[1], Mr Hakim Kettab[1], Mr Gérard Maruani[1], Mr Pascal Houillier[1], Ms Rosa Maria Bruno[1]
[1]University of Paris, Paris, France, [2]University of Perugia, Perugia, Italy
**Objectives:** Hypoparathyroidism may induce arterial damage, but underlying mechanisms are not well established. Few papers have addressed the topic, reporting an increase in central values and in carotid intima-media thickness [1][2]. The aim of this study was to determine the presence of early vascular ageing signs in large and small arteries.
**Methods:** 17 patients affected by hypoparathyroidism and 17 healthy controls (matched for age, sex, blood pressure and risk factors) were studied to obtain carotid-femoral PWV, carotid and digital intima-media thickness and stiffness by standard and ultra-high frequency ultrasound respectively. In the patients affected by hypoparathyroidism, complete calcium-phosphorus metabolism blood tests were taken.
**Results:** Carotid distensibility was significatively lower in the patients group ($18.7 \pm 6.0$ vs $39.4 \pm 10.5$ $p<0.001$), with a marked increase in carotid stiffness ($7.6 \pm 1.3$ vs $5.3 \pm 0.9$ m/s, $p<0.001$). No significant difference was reported in PWV and central pressure among the groups. A reduced digital artery diameter was present (mean diameter $0.8 \pm 0.1$ vs $1.0 \pm 0.2$ mm $p=0.005$), with a reduced stiffness ($5.6 \pm 2.5$ vs $9.2 \pm 4.9$ m/s $p<0.05$). A positive correlation between carotid artery stiffness and Calcium–Phosphorus product was found in the patients group ($r=0.661$ $p=0.037$).
**Conclusions:** Hypoparathyroidism is characterized by increased carotid artery stiffness. Digital stiffness (UFHS) is paradoxically reduced (possibly as compensatory mechanism), with hypotrophic remodeling. Our data support altered calcium-phosphorus metabolism as mechanism of arterial damage.

### 3.5
### Associations of lower limb atherosclerosis and arteriosclerosis with cardiovascular risk factors and disease in older adults: the ARIC study

**Patricia Pagan Lassalle**[1], Keeron Stone[2], Simon Fryer[2], James Faulkner[3], Michelle Meyer[1], Kevin Heffernan[4], Anna Kucharska-Newton[1,5], Gabriel Zieff[1], Craig Paterson[2], Kunihiro Matsushita[6], Timothy Hughes[7], Hirofumi Tanaka[8], Lee Stoner[1]
[1]The University Of North Carolina At Chapel Hill, Chapel Hill, United States, [2]University of Gloucestershire, Gloucestershire, United Kingdom, [3]University of Winchester, Winchester, United Kingdom, [4]Syracuse University, Syracuse, United States, [5]University of Kentucky, Lexington, United States, [6]Johns Hopkins, Baltimore, United States, [7]Wake Forest, Winston Salem, United States, [8]The University of Texas, Austin, United States
**Background:** Atherosclerosis and arteriosclerosis contribute to advanced vascular aging and cardiovascular disease (CVD) risk. Both processes can be assessed simply in the lower-limbs and reflect systemic pathology. However, only atherosclerosis is routinely assessed, typically via ankle-brachial index (ABI). Arteriosclerosis can be assessed using femoral-ankle pulse- wave velocity (faPWV), but no studies have identified whether ABI and faPWV similarly associate with overt CVD and risk factors, nor whether faPWV confers additional information.

**Objectives:** i) Compare independent associations of ABI and faPWV with traditional CVD risk factors, including age, sex, systolic blood pressure (SBP), high-density lipoprotein (HDL), total cholesterol (TC), smoking, and diabetes. ii) Determine the independent and additive associations of ABI and faPWV with a composite measure of prevalent CVD status.
**Methods:** We evaluated 4,330 older-aged ($75.3 \pm 5.0$ years) adults using an oscillometric device. Associations between ABI and faPWV with CVD risk factors and CVD were determined using mixed-model linear- and logistic-regression analyses.
**Results:** Both ABI and faPWV were associated with age, HDL, and smoking. ABI was associated with sex, TC, diabetes. faPWV was associated with SBP. Both ABI and faPWV were inversely associated with CVD. In categorical analysis low ABI ($\leq 0.9$ vs. $>0.9$) and low faPWV ($\leq 9.94$ vs. $>9.94$) increased the odds of CVD by 2.41-fold (95% CI 1.85, 3.17) and 1.46-fold (95% CI 1.23, 1.74), respectively. The inverse association between faPWV and CVD was independent of ABI and CVD risk factors.
**Conclusion:** Both ABI and faPWV are independently associated with CVD risk factors and prevalent CVD. The assessment of faPWV may confer additional risk information beyond ABI.

### 3.6

### Developing a questionnaire on the knowledge and perceptions of people working with vascular ageing

**Prof. Areti Triantafyllou**[1], Ms Stavria Artemis Elia[2], Chloe Park[3], Rachel Climie[4], Christopher C. Mayer[5], Andrie G. Panayiotou[2]
[1]3rd Dep. of Internal Medicine, Aristotle University of Thessaloniki, Thessaloniki, Greece, [2]Cyprus University Of Technology, Limassol, Cyprus, [3]University College London, London, UK, [4]BAKER HEART AND DIABETES INSTITUTE, Melbourne, Australia, [5]AIT Austrian Institute of Technology GmbH, Vienna, Austria

**Objective:** Vascular ageing (VA) is an emerging cardiovascular disease risk predictor, and an appealing therapeutic target, however, it remains underused in clinical practice. We aimed to create a questionnaire to investigate awareness and perceptions of healthcare providers and people working in the field of VA.

**Methods:** An online qualitative questionnaire with open-ended questions was developed based on key questions that were identified by an expert working group in the Network for Research in Vascular Ageing (VascAgeNet) COST Action/CA18216. The questionnaire was distributed to participants working in the field of VA. The questions were intentionally left open-ended to assess knowledge and perceptions, without guiding answers.

**Results:** Out of the 127 responses, 104 questionnaires were complete. The mean age of participants was 42.5 ± 10.5 years, 60.6% male, 54.8% were academics and/or researchers, 14.4% clinical doctors, and 9.6% worked in the industry. 67.3% reported that VA is important to them. For an explanation of importance, 14.4% reported personal/research interest, and 46.2% reported a combination of the following: improved primary prevention-CV risk evaluation/treatment strategies, marker of overall health, and underlying cause of diseases. 16.4% and 53.9% reported using VA measurements in clinical practice and research, respectively. Amongst the most frequently reported limiting factors were time, lack of reimbursement of tests, cost of equipment, and lack of guidelines for VA measurements.

**Conclusion:** Regardless of occupation and specialty, the overall perceptions of participants were positive. Future work will investigate why, despite the positive perceptions regarding VA measurements, the use of VA measures in clinical practice remains low.

### 3.7

### The VaSera heart-to-ankle pulse wave velocity is a nearly diastolic wave speed metric

**Mr Alessandro Giudici**[1,2], Professor Ashraf W. Khir[2], Professor Koen D. Reesink[1], Professor Tammo Delhaas[1], Professor Bart Spronck[1,3]
[1]Department of Biomedical Engineering, CARIM School for Cardiovascular Diseases, Maastricht University, Maastricht, Netherlands, [2]Biomedical Engineering Research Group, Brunel University London, Uxbridge, United Kingdom,[3]Department of Biomedical Engineering, School of Engineering and Applied Science, Yale University, New Haven, USA

**Background:** Estimation of heart-to-ankle arterial stiffness, as quantified by heart-to-ankle pulse wave velocity (haPWV), relies on the summation of the time differences between: 1) the aortic valve closing sound and notch of the brachial pressure waveform (tb), and 2) the feet of brachial and ankle pressure waveforms (tba). Cardio-ankle vascular index (CAVI) and $CAVI_0$ aim to normalise haPWV for blood pressure (BP) at the time of measurement. As tb and tba are determined at diastolic (DBP) and dicrotic notch ($P_{notch}$) BP, respectively (**Fig. A**), the choice of the haPWV-relevant normalisation pressure ($P_{haPWV}$) is not trivial and represents a major difference between $CAVI_0$, assuming $P_{haPWV}$ = DBP, and CAVI, assuming $P_{haPWV} P_m$ = (SBP + DBP)/2 (SBP: systolic BP). We aimed to analytically estimate $P_{haPWV}$.

**Methods:** Consistent with CAVI/$CAVI_0$ assumptions, the arterial tree was modelled as a system of tubes with exponential pressure-diameter relationship and uniform stiffness index. PWV then follows from [1,2]: where $P$ = {DBP, $P_{notch}$}, $P_{ref}$ = 100 mmHg and = 1050 kg/m$^3$. The resulting PWVs, together with the heart-to-ankle ($L_{ha}$) and heart-to-brachial ($L_{hb}$) path lengths [3], allow estimation of: a weighted average of which yields Given $P_{notch}$ = 0.55·DBP + 0.45·SBP [4].

**Results:** Choosing SBP/DBP = 120/80 mmHg and = 10 yielded tb = 0.048 s, tba = 0.187 s, $w_1$ = 0.908 and $w_2$ = 0.092, implying that DBP and SBP account for 91% and 9% of *P*haPWV, respectively (**Fig. B**). This was only marginally influenced by BP: e.g., SBP/DBP = 160/90 mmHg yielded $w_1$ = 0.912 and $w_2$ = 0.088.

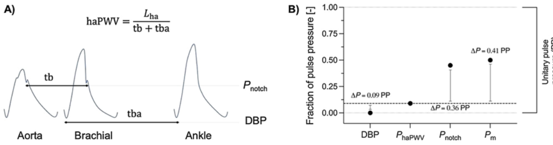

**Conclusions:** DBP offers a more accurate approximation of $P_{haPWV}$ than $P_{m.}$, supporting the utility of $CAVI_0$ as an enhancement of CAVI.

### 3.8

### Antithrombotic therapy in secondary and tertiary prevention for peripheral arterial disease: a network meta-analysis

**Dr Loes Willems**[1], Ms Dominique Maas[1], Dr Michel Reijnen[2], Dr Niels Riksen[1], Dr Hugo Ten Cate[3], Dr Rozemarijn Van der Vijver-Coppen[1], Dr. Clark Zeebregts[4], Dr. Gerjon Hannink[1], Dr. Michiel Warlé[1]
[1]Radboud University Medical Center, Nijmegen, The Netherlands, [2]Rijnstate Hospital, Arnhem, The Netherlands,[3]Maastricht University Medical Center, Maastricht, The Netherlands, [4]University Medical Center Groningen, Groningen, The Netherlands

**Purpose/Background/Objectives:** recent trials demonstrate the superiority of acetylsalicylic acid (ASA) with low-dose rivaroxaban in peripheral arterial disease (PAD), compared to ASA monotherapy.[1–2] An important question remains how the efficacy of ASA + rivaroxaban relates to clopidogrel, as the first choice antithrombotic therapy in PAD.[3] Therefore, we conducted a network meta-analysis on the efficacy of all antithrombotic regimens.

**Methods:** A systematic search was conducted of randomized controlled trials, published between 1995 and 2020. ASA was the universal comparator. The primary endpoint was major adverse cardiovascular events (MACE) and the safety endpoint was major bleeding. Secondary endpoints were all-cause mortality (ACM), major adverse limb events (MALE) and acute limb events (ALI).

**Results:** Twenty-eight randomized controlled trials were identified including 49.434 patients. Compared to ASA, MACE was less prevalent with clopidogrel [RR0.78, 95% CI 0.66–0.93], ASA + ticagrelor [RR0.79 95% CI 0.64–0.97], ticagrelor [RR0.79, 95% CI 0.65–0.97], and ASA + rivaroxaban [RR0.84, 95% CI 0.76–0.93] (A). Major bleedings occurred more frequently with ASA + rivaroxaban [RR1.46, 95% CI 1.18–1.80], rivaroxaban [RR1.47, 95% CI 1.06–2.05], vitamin K antagonists [RR1.93, 95% CI 1.41–2.64], ASA + vitamin K antagonists [RR2.77, 95% CI 1.93–3.97] and ASA + cilostazol [RR2.88, 95% CI 0.12—67.41] (B). All regimens were non-superior to ASA concerning ACM (C) and MALE (D). ASA + rivaroxaban was more effective in preventing ALI [RR0.67, 95% CI 0.55–0.80] (E).

**Conclusion:** Both clopidogrel and ASA + rivaroxaban were more effective than ASA in secondary prevention, however, ASA + rivaroxaban increases the risk of major bleeding. Concerning tertiary prevention, information on clopidogrel is lacking, while ASA + rivaroxaban is more effective in preventing ALI.

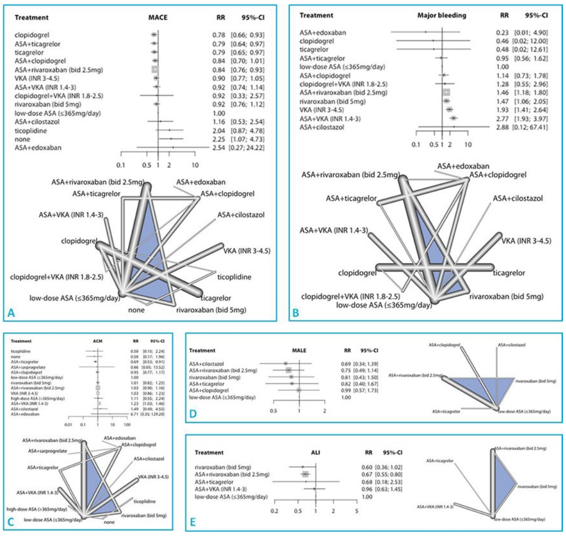

### 3.9

### Superior effect of community-based high-intensity interval exercise for reducing blood pressure and arterial stiffness in low-income older women

**Mrs. Vanessa Amaral**[1], Mr Gabriel Zanini[1], Ms Isabela Roque Marçal[1], Miss Bianca Fernandes[1], Mr. Lucas Bueno Gimenez[1], Miss Fernanda Bianchi Souza[1], Mr Gabriel Locato[1], PhD Student Awassi Yuphiwa Ngomane[1], Dr. Emmanuel Gomes Ciolac[1]

[1]Universidade Estadual Paulista, Bauru, Brazil

**Aim:** To assess the effect of different community-based exercise programs on blood pressure, arterial stiffness, and functional capacity in low-income older women.

**Methods:** 92 insufficiently active low-income older women (71.2 ± 5.21 years) were randomly assigned to community-based high-intensity interval training combined with resistance training (HIIT + RT, n = 34), moderate-intensity continuous training combined with resistance training (MICT + RT, n = 38) or resistance training alone (RT, n = 20) for nine months. Anthropometric (weight, waist circumference and body mass index [BMI]), hemodynamic (resting blood pressure [BP], heart rate and arterial stiffness [carotid-femoral pulse wave velocity—PWV]), and functional capacity (handgrip strength and 6-min walking test [6MWT]) were assessed before, nine months after training, and three months after training interruption.

**Results:** Significant improvements ($P < 0.05$) on waist circumference, handgrip strength and 6MWT were found after both HIIT-RT (waist circumference: − 3 cm; handgrip strength: + 4 kgf; 6MWT: + 83 m) and MICT-RT (waist circumference: − 3 cm; handgrip strength: 4 kgf; 6MWT: + 46 m), while only handgrip strength (+ 4 kgf) and 6MWT (+ 42 m) improved after RT. However, systolic BP and PWV reduced ($P < 0.01$) only after HIIT-RT (− 7 mmHg and − 0.6 m/s, respectively). In addition, waist circumference, handgrip strength, 6MWT and PWV (Fig. 1) improvements were maintained (at least in part) three months after HIIT + RT interruption, while only waist circumference and handgrip improvements were maintained after MICT + RT interruption, and no improvements were maintained after RT interruption.

**Conclusion:** Community-based HIIT + RT was superior to other exercise interventions for improving systolic BP and arterial stiffness of low-income older women.

**Fig. 1** Arterial stiffness before, nine months after training and three months after training interruption.

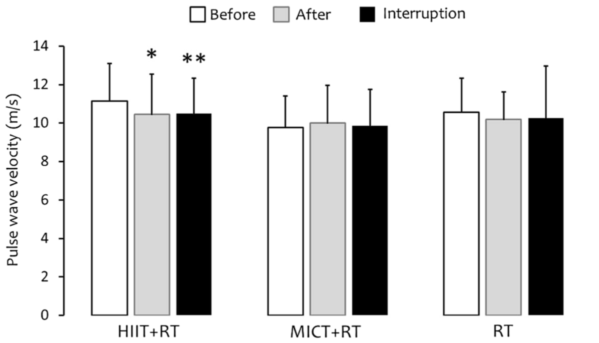

HIIT + RT: high-intensity interval training combined with resistance training; MICT + RT: moderate-intensity continuous training combined with resistance training; RT: resistance training alone; Asterisk indicates significant difference from before training (*: $P = 0.01$; **: $P = 0.001$).

### 3.10

### Evaluation of hemodynamic and vascular responses after a continuous exercise session of moderate intensity and high intensity intervals in individuals with normal and high normal blood pressure

**Miss Sara Rodrigues**[1], Miss Renata Verardino[1], Mr Marcel Costa[1], Miss Valéria Costa-Hong[1], Miss Maria Alves[1], Mr Luiz Bortolotto[1]

[1]InCor HC FM USP, São Paulo, Brazil

**Purpose:** Physical exercise (PE) prevents cardiovascular diseases[1]. There is no consensus if different intensities of PE changes arterial stiffness—by pulse wave velocity (PWV) and augmentation index (AIx) -, a marker of cardiovascular risk[2], related to blood pressure (BP).

**Objective**: In normotensive—normal BP (120–129/80–84 mmHg) and high normal BP (130–139/85–89 mmHg), compare: 1st) arterial stiffness after one session of moderate-intensity continuous PE (MICPE) and high-intensity interval PE (HIIPE). 2nd) BP after MICPE and HIIPE.

**Methods**: PE intensity and equalized energy expenditure defined by cardiopulmonary exercise test. Individuals randomized to PE sessions, performed in cross-over fashion. PWV and AIx were measured at rest, immediately after and 24 h after HIIPE and MICPE, compared among all moments (baseline, immediately and 24 h after each session). Ambulatory BP monitoring-24 h (ABPM-24 h) was performed after rest and each session, using the first two hours for comparison.

**Results**: Individuals (N = 29; 76%women; age = 48 ± 7 years; BMI = 28.3 ± 4 kg/m$^2$; SBP = 126 ± 9; DBP = 84 ± 4 mmHg) had lower ($p < 0.01$) AIx after MICPE (27.1 ± 2.0) and HIIPE (22.7 ± 2.2), than baseline (33.0 ± 1.8). AIx after MICPE (27.1 ± 2.0) was lower ($p < 0.05$) than MICPE24h (30.6 ± 2.3). AIx after HIIPE (22.7 ± 2.2) was lower ($p = 0.01$) than MICPE (27.1 ± 2.0), and than HIIPE24h (32.2 ± 1.9). SystolicBP (2 h) reduced after both sessions –MICPE (128 ± 2) and HIIPE (127 ± 2 mmHg), compared to baseline (131 ± 2 mmHg; $p = 0.02$). There was no difference in PWV among times, and between individuals with normal BP and high normal BP.

**Conclusion**: In normotensive, one PE session, regardless of intensity, reduces systolicBP during the first 2 h and AIx immediately after, returning to baseline values 24 h after PE session.

### 7.1
### Pulse Wave Velocity for 24-h Ambulatory Blood Pressure Monitoring

**M.Sc. Fabian Beutel**[1,2], B.Eng. Chaim Zax[2], B.Eng. Jesse Kling[2], B.Eng. Anthony van der Heijden[2], Ph.D. Chris Van Hoof[1,3], Ph.D. Evelien Hermeling[2]
[1]KU Leuven, Leuven, Belgium, [2]imec The Netherlands, Eindhoven, The Netherlands, [3]imec, Leuven, Belgium

**Background:** Ambulatory blood pressure monitoring (ABPM) is a much stronger cardiovascular risk predictor than office BP, allowing for identification of white-coat and borderline hypertensives (1), and prognostically relevant patterns of nocturnal surges (2). However, ABPM availability in clinical practice is limited and causes patient discomfort from frequent cuff inflations (1). Therefore, we propose a wearable, cuff-less system to track BP changes from inherently related arterial pulse wave velocity (PWV).

**Methods:** Data was acquired from 10 subjects (35±7 years) over 24 h in free living conditions. The novel wearable system (imec,NL) combines electrocardiogram (ECG) and photoplethysmogram (PPG) signals to obtain beat-to-beat pulse arrival time (PAT), converted to PWV via arterial path length (L). PWV and heart rate (HR) data was smoothed using a 1-h moving average window and values were extracted corresponding to simultaneously acquired intermittent ABPM readings (Suntech Medical Inc.,USA). Mean values were subtracted per subject to obtain changes per variable (D). Unbiased multivariate regression models were generated to estimate DSBP and DDBP responses from DPWV and DHR predictors.

**Results:** BP tracking errors were conform with AAMI standard error criteria (5 mmHg and SD 8 mmHg) (3), achieving = − 0.2/0.0 mmHg and SD = 7.6/6.0 mmHg for SBP/ DBP. Accounting for the subtracted means demonstrated accurate estimations over a wide absolute range (55–105 mmHg mean BP) and a 90% classification accuracy for diagnostic hypertension thresholds, contrasting 62% if BP was assumed constant.

**Conclusion:** The proposed system demonstrates significant predictive utility for BP changes and may facilitate unobtrusive and cuff- less ABPM at high temporal resolution.

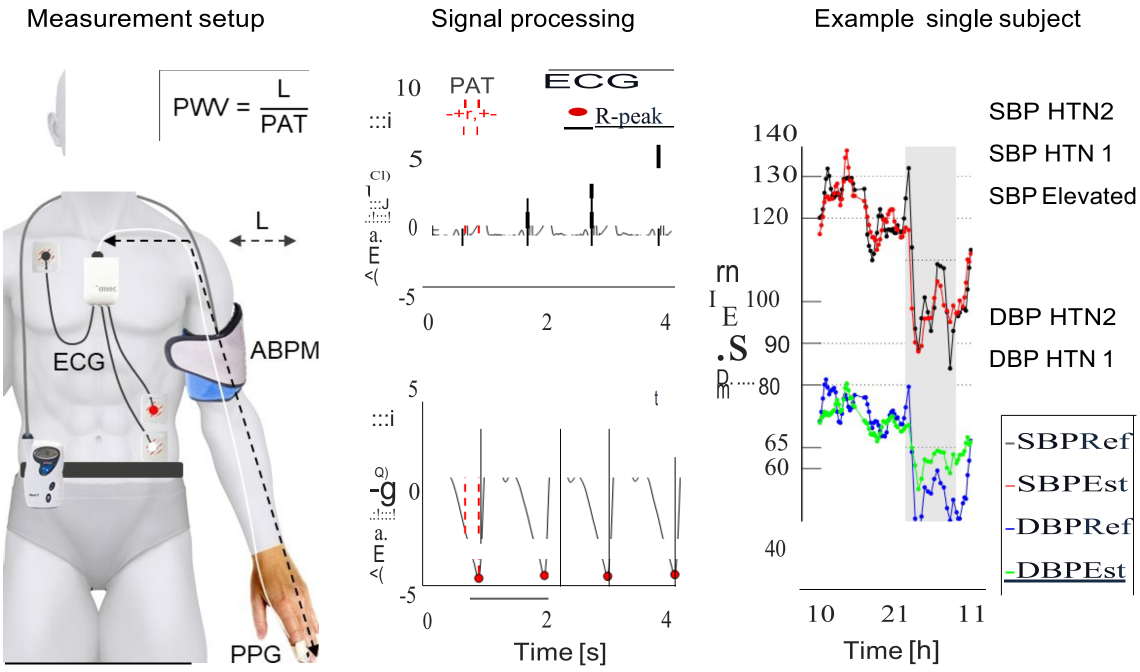

### 7.2

### Intra-Operative Video-Based Measurement of Biaxial Strains of the Ascending Thoracic Aorta

**MSc Shaiv Parikh**[1], MSc Berta Ganizada[2], Mr. Gijs Debeij[2], Dr. Ehsan Natour[2], Prof. Dr. Jos Maessen[2], Dr. Bart Spronck[1], Prof. Dr. Leon Schurgers[3], Prof. Dr. Tammo Delhaas[1], Dr. Wouter Huberts[1], Dr. Elham Bidar[2], Dr. Koen Reesink[1]

[1]Department of Biomedical Engineering, CARIM School for Cardiovascular Diseases, Maastricht University, Maastricht, Netherlands, [2]Department of Cardiothoracic Surgery, Heart & Vascular Centre, Maastricht University Medical Centre, Maastricht, Netherlands, [3]Department of Biochemistry, CARIM School for Cardiovascular Diseases, Maastricht University, Maastricht, Netherlands

**Background:** Local biaxial deformation measurements are essential for the in-depth investigation of tissue properties and remodeling of the ascending thoracic aorta, particularly in aneurysm formation[1]. Current clinical imaging modalities pose limitations around the resolution and tracking of anatomical markers[2–4].

**Methods:** We evaluated a new intra-operative video-based method to assess local biaxial strains of the ascending thoracic aorta **(Figure A)**. In patients undergoing open-thorax surgery, two pairs of diametrically opposite felt markers were sutured on the adventitial surface of the aorta after the sternotomy **(Figure B)**. Videos of markers were captured at low- and high- pressure conditions **(Figure C)**, and marker tracking was performed using an in-house tracking algorithm to determine local biaxial strains. We obtained in 30 patients repeated biaxial strain measurements, for each pressure condition. **(Figure D, E)**.

**Results:** Precision was acceptable, with coefficients of variation for biaxial strains remaining below 20% and the magnitude of strains we obtained (range: 0.02–0.05) is in line with previous reports, using clinical imaging. Interestingly, with our four-marker arrangement, we were able to detect significant local differences in longitudinal strain as well as in circumferential strain **(Figure D, E)**.

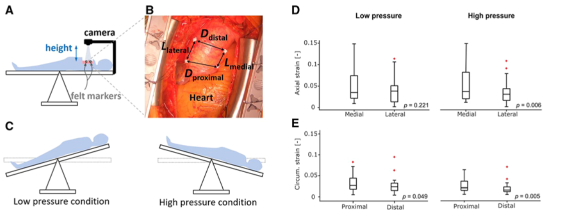

**Figure.** (A,B,C): Key elements of the intra-operative, video-based strain measurement method. (A): Intraoperative set-up. (B): Single video image showing heart on bottom and markers sutured on the adventitia of an ascending aortic aneurysm. Arrows between markers define the positions at which cranial and caudal (circumferential) strains, and medial and lateral (axial) strains were assessed. (C): Low and high transmural pressure conditions were created by tilting the table. Tilted table position for low-pressure condition shown is anti-Trendelenburg, while tilted-table position for high-pressure condition shown is Trendelenburg position. Horizontal position of the table is referred to as neutral position. (D,E): Potential for detecting local strain differences. *p*-values indicate paired Wilcoxon Signed Ranks testing, with $n = 30$ for low and $n = 29$ for high pressure. Boxplots indicate medians [25th, 75th percentile], with whiskers indicating variability beyond the first and the third quartile, while red plus signs are the outliers. (D): Lateral axial strains tended to be lower than those captured medially, but only achieving statistical significance in the high-pressure condition. (E): Cranial circumferential strains tended to be significantly lower than at the caudal location. Note: one outlier not shown in B (values for caudal and cranial strains > 0.2 for both pressure conditions).

**Conclusion:** The proposed method enables the assessment of local aortic biaxial strains and may enable new, clinically informed mechanistic studies using biomechanical modeling as well as mechanobiological profiling.

### 7.3

### On the estimation of arterial compliance from carotid pressure waveform

**Ms Vasiliki Bikia**[1], Professor Patrick Segers[2], Mr Georgios Rovas[1], Ms Stamatia Pagoulatou[1], Professor Nikolaos Stergiopulos[1]

[1]École Polytechnique Fédérale De Lausanne, Lausanne, Switzerland, [2]IBiTech, University of Ghent, Ghent, Belgium

**Abstract**

**Background:** Total arterial compliance ($C_T$) is a biomechanical property of the arterial tree with great pathophysiological importance (1,2) and thus its estimation via a simple, accurate method is valuable. Direct noninvasive measurement of $C_T$ is not feasible in the clinical practice. Previous methods for indirect $C_T$ estimation require noninvasive, yet complex and expensive, recordings of the central pressure and flow (3,4).

**Methods:** This study introduces a novel, noninvasive method for estimating $C_T$ from a single carotid waveform measurement using neural networks. Features were extracted from the carotid wave and were combined with demographic data. A prediction pipeline was adopted for estimating $C_T$ using (i) firstly, a feature-based analysis ($ANN_{FB}$), and (ii) secondly, the raw carotid pulse wave ($ANN_{RAW}$) as input. The methodology was evaluated using the large Asklepios cohort ($n = 2256$).

**Results:** Accurate estimates of $C_T$ were yielded for both prediction schemes (Figure); namely, $r = 0.83$/normalized- RMSE = 9.6% for $ANN_{FB}$, and $r = 0.83$/normalized-MSE = 9.7% for $ANN_{RAW}$, respectively.

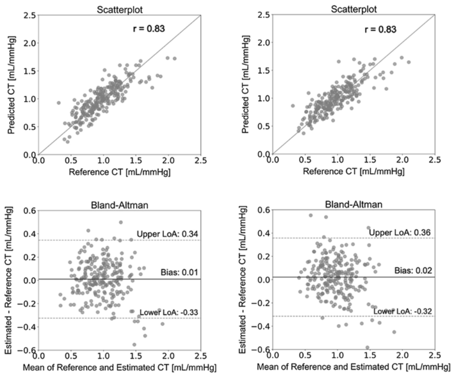

**Figure.** Comparison between predicted and reference data. Scatterplot and Bland–Altman plot between the predicted $C_T$ and the reference $C_T$ for $ANN_{FB}$ (left panel), and $ANN_{RAW}$ (right panel). The solid line of the scatterplots represents equality. In Bland–Altman plots, limits of agreement (LoA) are defined by the two horizontal dashed lines.

**Conclusions:** The method may allow for easily applicable and convenient CT monitoring. Such an approach could suggest promising applications, ranging from fast and cost-efficient hemodynamical monitoring by the physician to integration in wearable technologies.

### 7.4

### Assessing radiotherapy-induced carotid vasculopathy using ultrasound after unilateral irradiation

**MD Judith Pruijssen**[1], MD PhD Joyce Wilbers[1], MD Ashwin Wenmakers[1], MD PhD Jacqueline Loonen[1], Prof Dr Chris de Korte[1,2], Prof MD PhD Johannes Kaanders[1], PhD Hendrik Hansen[1]

[1]Radboud University Medical Center, Nijmegen, Netherlands, [2]University of Twente, Twente, Netherlands

**Background:** Improved diagnostic and therapeutic regimens have increased head and neck cancer (HNC) survival. However, the younger, growing survivor population is prone to long-term treatment-related vascular complications. Irradiated patients have a minimally twofold stroke risk compared to the general population (1). However, the pathophysiology of radiation- induced vasculopathy is unclear. Vascular stiffness might be a biomarker of this injury. We investigated whether irradiated compared to unirradiated carotids in unilaterally irradiated patients showed alterations in stiffness-related parameters, shear wave velocity (SWV) and pulse wave velocity (PWV), and intima-media thickness (IMT).

**Methods:** Twenty-six patients (41 ± 9 years), median 7 years after unilateral radiotherapy for head and neck tumors underwent bilateral carotid ultrasounds with an Aixplorer Ultimate system (Supersonic Imagine, France) with SL18-5 and SL10-2 transducers. IMT, begin- and end-systolic PWV, and average SWV were assessed in the proximal-, mid-, and distal common (CCA) and internal carotid artery (ICA). Three consecutive measurements were averaged. Differences between irradiated and unirradiated carotids were tested using Wilcoxon signed-rank tests. Radiation dose–effect relations were explored with linear regression.

**Results:** CCA-IMT was higher in the irradiated than unirradiated carotids (Table 1). Although overall stiffness seemed higher in irradiated carotids, differences were only statistically significant for anterior mid-CCA and posterior ICA SWV. A dose–effect relation seemed apparent for end-systolic PWV ($\beta = 0.051$/Gray, $p = 0.07$), not for IMT or SWV.

**Conclusion:** Some IMT and stiffness increases were observed in irradiated carotids. To further investigate vascular stiffness as a biomarker for radiation-induced vasculopathy a larger, typical HNC cohorts (higher age/radiation doses/number of cardiovascular risk factors) is required.

**Table 1**: Difference between irradiated and non-irradiated side and radiotherapy dose-effect correlation (n=26).

| Parameter | Location | Time point | Difference (irr-co)[1] | P | Regr coeff radiation dose (95%-CI)[2] | P |
|---|---|---|---|---|---|---|
| **IMT (mm)** | Distal CCA | Diastole | 0.02 [0.00-0.09] | 0.001* | -0.001 (-0.003-0.002) | 0.60 |
| | ICA | | 0.00 [-0.01-0.01] | 0.59 | 0.000 (0.000-0.001) | 0.20 |
| **PWV (m/s)** | Mid CCA | Begin-systole | 0.51 [-1.02-1.32] | 0.36 | 0.040 (-0.032-0.111) | 0.26 |
| | | End-systole | 0.11 [-0.43-1.45] | 0.24 | 0.052 (-0.005-0.110) | 0.07 |
| **SWV (m/s)** | Proximal CCA | Averaged over cardiac cycle | A: -0.09 [-0.56-0.35] | 0.48 | A: 0.009 (-0.025-0.043) | 0.61 |
| | | | P: 0.04 [-0.52-0.71] | 0.42 | P: 0.024 (-0.007-0.055) | 0.12 |
| | Mid CCA | | A: 0.43 [-0.19-0.68] | 0.03* | A: 0.003 (-0.023-0.029) | 0.79 |
| | | | P: 0.36 [-0.65-0.73 | 0.46 | P: 0.011 (-0.026-0.047) | 0.55 |
| | Distal CCA | | A: 0.17 [-0.06-0.65] | 0.06 | A: -0.004 (-0.028-0.020) | 0.75 |
| | | | P: -0.15 [-0.59-0.46] | 0.74 | P: -0.006 (-0.032-0.020) | 0.66 |
| | ICA | | A: 0.28 [-0.54-0.85] | 0.34 | A: 0.009 (-0.024-0.043) | 0.57 |
| | | | P: 0.62 [-0.24-1.22] | 0.01* | P: 0.001 (-0.022-0.024) | 0.93 |

IMT = intima-media thickness; PWV = pulse wave velocity; SWV = shear wave velocity; A = anterior; P = posterior. [1] Data expressed as median [inter-quartile range]. [2] Based on difference in radiotherapy dose between irradiated and control carotid (in Gray) versus difference in ultrasound parameters. * Significant difference ($p<0.05$) between irradiated and control side.

### 7.5

### Sublingual nitroglycerine ingestion is associated with an increase rather than decrease in brachial-artery retrograde blood flow in healthy human subjects

**Dr Smriti Badhwar**[1], Dr. Dinu Chandran[1], Prof Ashok Jaryal[1], Prof Rajiv Narang[1], Prof Chetan Patel[1], Prof Kishore Kumar Deepak[1]
[1]All India Institute of Medical Sciences, New Delhi, India

**Background:** Nitroglycerine can reduce left ventricular afterload by decreasing amplitude of wave reflections (1). Increase in brachial-artery retrograde flow is associated with endothelial dysfunction. It was hypothesized that nitroglycerine mediated reduction in peripheral wave reflections would reduce brachial-artery retrograde flow. The current study aimed to evaluate effect of nitroglycerine on brachial-artery retrograde flow.

**Methods:** Brachial artery retrograde flow velocity and diameter were measured in duplex mode using Pulsed-wave doppler (M7, MindRay) at baseline and 5 min after sublingual Isosorbide dinitrate (5 mg) ingestion in 32 healthy subjects. Time-averaged mean anterograde and retrograde blood flow velocity (ABRV and RBFV) were measured, and blood flows (ABF and RBF) were calculated from the velocities and corresponding diameters.

**Results:** There was significant increase in RBFV at 4th and 5th min (1.96 1.25 vs 1.60 0.99 vs1.89 0.97 vs 1.80 0.89 vs 2.38 1.33 vs 2.67 1.27 cm/s) and RBF at 5th min (0.32 0.22 vs 0.23 0.16 vs 0.27 0.19 vs 0.41 0.32 vs 0.48 0.32 $cm^3/s$) after nitrate ingestion. ABFV showed a significant decrease from baseline to 5th min (10.6 4.0 vs 8.92 4.09 vs 7.66 3.31 vs 6.73 2.63 vs 7.11 vs 2.80 vs 7.12 2.60 cm/s), while ABF decreased from baseline to 3rd min and then increased again at 4th and 5th min (1.33 0.53 vs 1.23 0.57 vs 1.11 0.53 vs 1.06 0.43 vs 1.20 0.56 vs 1.20 0.48 $cm^3/s$).

**Conclusions:** A paradoxical increase in RBF is seen with nitroglycerine ingestion which may be a result of acute-reflex mediated decrease in aortic distensibility. Alternatively, persistence of increase in RBF may be a possible mechanism for nitrate-induced endothelial dysfunction and needs to be investigated.

### P.1

### Higher systolic blood pressure in females compared to males with similar brachial cuff systolic blood pressure: an effect mediated by height

**Ms Yasmine Abbaoui[1]**, Dr Florence Lamarche[1], Dr Annie-Claire Nadeau-Fredette[2], Dr François Madore[1], Dr Mohsen Agharazii[3], Dr Remi Goupil[1]
[1]Hopital de Sacré-Coeur de Montréal, [2]Hôpital Maisonneuve-Rosemont, [3]CHU de Québec

**Background:** Females have higher risks of cardiovascular events compared to males with similar BP. We assessed whether the accuracy of non-invasive BP measurements towards aortic BP could be influenced by biological sex.

**Methods:** We enrolled 500 patients undergoing coronary angiography for simultaneous measurements of invasive aortic BP with non-invasive BP (Mobil-o-Graph device). Linear regression and mediation analyses were used to adjust for potential confounders in the relationship between biological sex and brachial cuff BP accuracy.

**Results:** Of 500 participants, 145 were females. Several characteristics were different in males and females (Table). Brachial cuff SBP was identical in both groups whereas aortic SBP was 6.2 mmHg higher in females ($p < 0.001$). As such, the brachial cuff appreciably underestimated aortic SBP in females but not in males. In an adjusted linear regression model, only height and pulse pressure were independently associated with the accuracy of brachial cuff SBP. This effect of sex on accuracy was mostly mediated by height (3.5 mmHg; 95% CI 1.4 to 5.6; 57% mediation) to an extent that the direct effect of sex became non-significant (2.9 mmHg; 95% CI -0.3 to 6.2).

**Conclusion:** Females have higher aortic SBPs than males with identical brachial cuff SBP, which is mostly mediated by a lower height. This unrecognized higher aortic SBP provides indirect evidence to support the increased cardiovascular risk of females compared with males with similar brachial cuff SBP.

| **Clinical characteristics** | **Female (n=145)** | **Male (n=355)** | **p-value** |
|---|---|---|---|
| Age | 66 ± 11 | 66 ± 10 | 0.6 |
| Height (cm) | 159 ± 7 | 174 ± 7 | <0.001 |
| Weight (kg) | 74 ± 18 | 86 ± 18 | <0.001 |
| BMI (kg/m²) | 29 ± 6 | 28 ± 5 | 0.3 |
| Active smoking | 26% | 24% | 0.6 |
| Diabetes | 26% | 29% | 0.5 |
| eGFR (mL/min/1.73m²) | 79 ± 18 | 81 ± 17 | 0.5 |
| Anti-hypertensive medication | 76% | 81% | 0.2 |
| Cuff Brachial SBP (mmHg) | 125 ± 18 | 124 ± 16 | 1.0 |
| Cuff brachial DBP (mmHg) | 74 ± 11 | 78 ± 11 | <0.001 |
| Brachial cuff PP (mmHg) | 51 ± 13 | 47 ± 11 | <0.001 |
| Heart rate (bpm) | 71 ± 13 | 66 ± 11 | <0.001 |
| Aortic pulse wave velocity (m/s) | 9.4 ± 2.1 | 9.4 ± 1.7 | 0.9 |
| Augmentation index @ 75 bmp | 26 ± 13 | 17 ± 13 | <0.001 |
| **Accuracy** | | | |
| Invasive Aortic SBP | 131 ± 22 | 125 ± 20 | <0.001 |
| Difference with Invasive Aortic SBP | | | |
| Cuff Brachial SBP | -6.5 ± 12.1 | -0.3 ± 11.7 | <0.001 |
| Cuff Type I central SBP | -17.3 ± 13.1 | -8.8 ± 13.1 | <0.001 |
| Cuff Type II central SBP | 0.6 ± 15.3 | 8.3 ± 14.2 | <0.001 |

Mean differences represent the difference between non-invasive BP and aortic BP and are expressed with ± SD. Type I central BP is obtained through calibration with brachial cuff SBP and DBP. Type II central BP is obtained through calibration with brachial cuff mean BP and DBP.

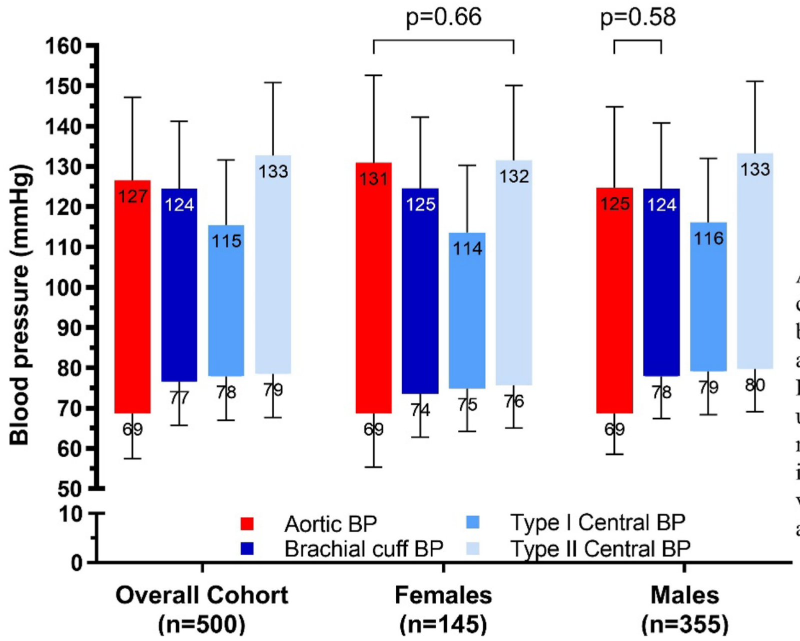

All pair-wise comparisons between aortic and non-invasive BPs are p<0.001 unless otherwise noted. Numbers indicate mean values with SDs as error bars.

### P.2

### Agreement of non-invasive blood pressure- and standard oscillometry-derived pulse wave velocities

**N Nathan Adams**[1], J Jillian Poles[1], G Gabriel Zieff[1], K Keeron Stone[2], C Craig Paterson[2], S Simon Fryer[2], M Michelle L. Meyer[1], L Lee Stoner[1]
[1]University of North Carolina at Chapel Hill, Chapel Hill, United States,
[2]University of Gloucestershire, Gloucester, United Kingdom

**We** assessed the overall and repeated measures agreement between a criterion measure of carotid-wrist pulse wave velocity (cwPWV) and a PWV measure derived from a continuous non-invasive blood pressure device ($PWV_{NIBP}$). A continuous method of PWV velocity would allow for the detection of acute changes in arterial stiffness.

**Methods:** cwPWV was measured with carotid artery and wrist cuffs. $PWV_{NIBP}$ was continuously obtained, with pulse wave transit time measured as the delay between the R wave of the electrocardiogram and the foot of the finger cuff pressure wave. Measurements were made twice in the supine posture, and then once in the seated position. The seated posture was used as an orthostatic challenge, to cause vascular changes for ascertainment of repeated-measures agreement. Mixed model regression was used to calculate overall agreement (independent of posture), and repeated-measures correlation was used to determine whether changes in the two measures agree. Acceptable intraclass correlation coefficient (ICC) agreement was set at 0.70.

**Results:** Complete data was collected for all 20 subjects (age: 22.5 ± 2.7 years, 14 female). The overall agreement between $PWV_{NIBP}$ and cwPWV was acceptable (ICC: 0.78, 95% CI [0.66, 0.86]). The repeated-measures agreement was weak (ICC: 0.30, 95% CI [0.05, 0.51]).
**Conclusions:** Acceptable overall agreement between $PWV_{NIBP}$ and cwPWV indicates that individual $PWV_{NIBP}$ may be useful for the determination of peripheral arterial stiffness. Repeated-measures agreement was weak, potentially due to lack of change in either measurement. Further research assessing different peripheral arterial changes may be necessary to determine whether $PWV_{NIBP}$ can accurately track vascular changes.

### P.3

### Analysis of wave intensity using non-invasive pressure waveform only: application to people with type 2 diabetes

**Dr Kunihiko Aizawa**[1], Prof. Alun D Hughes[2], Dr Francesco Casanova[1], Mr David M Mawson[1], Dr Kim M Gooding[1], Dr W David Strain[1], Dr Phillip E Gates[1], Prof. Isabel Gonçalves[3,4], Prof. Jan Nilsson[3], Prof. Faisel Kahn[5], Prof. Helen M Colhoun[6], Prof. Carlo Palombo[7], Prof. Kim H Parker[8], Prof. Angela C Shore[1]
[1]NIHR Exeter Clinical Research Facility, University of Exeter Medical School, Exeter, United Kingdom, [2]Institute of Cardiovascular Science, University College London, London, United Kingdom, [3]Department of Clinical Sciences, Lund University, Malmö, Sweden, [4]Department of Cardiology, Skåne University Hospital, Malmö, Sweden, [5]Division of Systems Medicine, University of Dundee, Dundee, United Kingdom, [6]Centre for Genomic and Experimental Medicine, University of Edinburgh, Edinburgh, United Kingdom, [7]Department of Surgical, Medical, Molecular and Critical Area Pathology, University of Pisa, Pisa, Italy, [8]Department of Bioengineering, Imperial College, London, United Kingdom
**Background:** Analysis of wave intensity provides valuable information about wave propagation in the arterial circulation. However, the need to measure both pressure and flow hinders its widespread use in large scale studies. A recently proposed method enables estimation of wave intensity patterns from non-invasive pressure waveforms.[1] We applied this method in type 2 diabetes (T2DM) to determine 1) whether it is feasible in T2DM, 2) whether wave intensity patterns differed between people with and without T2DM, and 3) whether the trajectories of those patterns, measured 3 years apart differed between cohorts.

**Methods:** We studied 227 individuals with T2DM (66.9 ± 7.9 years, 87F) and 99 without T2DM (66.0 ± 9.0 years, 43F). Radial pressure waveforms were recorded using a SphygmoCor system and aortic pressure waveforms were derived; these were analysed using custom-written software to calculate wave intensity.
**Results:** Wave intensity showed the typical pattern, and the magnitude of the forward compression and decompression waves were similar in both cohorts. However, the area of backward compression wave was smaller in people with T2DM than those without ($152 \times 10^4$ vs $180 \times 10^4$ J/m$^2$, $p = 0.025$), and wave reflection index was reduced (0.25 vs 0.29 au, $p = 0.050$). Both cohorts showed slightly upward trajectories in wave intensity patterns over 3 years, and the changes in magnitude of wave intensity were similar in both cohorts.
**Conclusions:** It is feasible to estimate wave intensity using the pressure-only method in T2DM. Using the method, wave reflection was smaller in people with T2DM compared with those without T2DM.

### P.4

### Longitudinal changes in aPWV in chronic obstructive pulmonary disease

Mrs Mahfoudha AL Shezawi[1,2], Maggie Munnery[2], Laura Watkeys[2], John Cockcroft[2], Nichola Gale[1], Barry McDonnell[2]
[1]Cardiff University, Cardiff, UK, [2]Cardiff Metropolitan University, Cardiff, UK
**Introduction:** Chronic Obstructive Pulmonary Disease (COPD) is characterised by irreversible airflow obstruction, chronic inflammation, increased cardiovascular disease (CVD) risk and mortality. Increased arterial stiffness measured by aortic pulse wave velocity (aPWV) may reflect subclinical CVD and is increased in COPD patients. However, the time course of longitudinal changes in aPWV have not been investigated in COPD. This study aimed to compare changes in aPWV over a 2-year and 6-year period, in patients with COPD and aged-matched controls.

**Methods:** This longitudinal study included patients with COPD and age-matched controls from the ARCADE study. Measurements included aPWV using the SphygmoCor system. Lung function was assessed by forced expiratory volume in the first second/forced vital capacity ($FEV_1$/FVC) using spirometry. Self-reported history of CVD and use of cardiovascular acting medications were recorded.
**Results:** Data were available in seventy-five patients with COPD and seventy-one controls (mean age 65 ± 7 years) at baseline, 2- and 6-years follow up. At each assessment time-point, patients with COPD had higher aPWV, lower $FEV_1$/FVC, and greater use of cardiovascular acting medications than controls (all at $p < 0.05$). Both groups showed a similar increase in aPWV over the 6 years (annual increase of 0.2 m/s/year, $p = 0.560$).
**Conclusion:** The similar relative change in aPWV observed in both groups may be explained by the progression in age. However, the higher baseline levels of aPWV in COPD were maintained and tracked over the 6-years, even in the presence of additional cardiovascular acting medications, suggesting a premature and consistently elevated vascular ageing process in COPD patients.

### P.5

### Assessment of vascular markers of large artery dysfunction and circulating biomarkers of endothelial dysfunction and thromboinflammation in patients with psoriasis

**Dr Panagiota Anyfanti**[1], Dr Anastasia Margouta[1], Dr Antonios Lazaridis[1], Dr Eleni Gavriilaki[1], Dr Efi Yiannaki[2], Dr Barbara Nikolaidou[1], Dr Areti Triantafyllou[1], Dr Elizabeth Lazaridou[3], Dr Stella Douma[1], Dr Aikaterini Patsatsi[3], Dr Eugenia Gkaliagkousi[1]
[1]3rd Department of Internal Medicine, Papageorgiou Hospital, Aristotle University of Thessaloniki, Thessaloniki, Greece,[2]Department of Hematology, Theagenion Cancer Center, Thessaloniki, Greece, [3]2nd Department of Dermatology and Venereology, General Hospital "Papageorgiou", Medical School Aristotle University of Thessaloniki,, Thessaloniki, Greece
**Purpose/background/objectives:** Cardiovascular risk is increased in psoriasis.[1] Endothelial (EMVs), platelet (PMVs) and erythrocyte (ErMVs) microvesicles represent novel biomarkers of endothelial dysfunction and thromboinflammation.[2] Pulse wave velocity (PWV) and carotid intima-media thickness (IMT) are robust measures of arterial stiffness and atherosclerosis. We tested whether the above markers of micro- and microcirculation are impaired in psoriasis.

**Methods:** Patients with psoriasis without cardiovascular diseases and controls matched for cardiovascular risk factors were studied. Psoriasis severity was assessed with PASI (Psoriasis Area Severity Index). Microvesicles were measured by a standardized flow cytometry protocol.[3,4] Carotid IMT and PWV were assessed with carotid ultrasound.
**Results:** Patients with psoriasis ($n = 41$) presented increased levels of both PMVs [354.9 (468) vs 151.0 (202)/μL, $p < 0.001$] and ErMVs [19.8 (96.4) vs 15.0 (25)/μL, $p = 0.046$] compared to controls ($n = 41$), whereas EMVs did not significantly differ. Nonsignificant differences were observed in carotid PWV and cIMT. Patients with higher PASI score (≥ 10) presented increased ErMVs compared to those with lower PASI score (< 10) [41.3 (123.5) vs 12.9 (98)/μL, $p = 0.047$]. Multivariate analysis showed that psoriasis independently predicted both PMVs ($p < 0.001$) and ErMVs ($p = 0.043$), while age ($p = 0.005$) and hypertension ($p < 0.001$) were independently associated with EMVs.
**Conclusions:** Circulating biomarkers of thromboinflammation, specifically PMVs and ErMVs, were increased in psoriasis before the clinical onset of overt cardiovascular complications and correlated with clinical disease activity. By contrast, vascular biomarkers of atherosclerosis and arterial stiffness were not increased. Further studies are needed to address the prognostic potential of microvesicles in psoriasis in terms of cardiovascular disease prediction.
**References**

1. Masson W, Lobo M, Molinero G. Psoriasis and Cardiovascular Risk: A Comprehensive Review. *Adv Ther*. 2020;37:2017–2033. https://doi.org/10.1007/s12325-020-01346-6
2. Lipets EN, Antonova OA, Shustova ON, Losenkova K V., Mazurov A V., Ataullakhanov FI. Use of Thrombodynamics for revealing the participation of platelet, erythrocyte, endothelial, and monocyte microparticles in coagulation activation and propagation. *PLoS One*. 2020;15(5):e0227932. https://doi.org/10.1371/journal.pone.0227932
3. Gkaliagkousi E, Gavriilaki E, Vasileiadis I, et al. Endothelial Microvesicles Circulating in Peripheral and Coronary Circulation Are Associated with Central Blood Pressure in Coronary Artery Disease. *Am J Hypertens*. 2019;32(12):1199–1205. https://doi.org/10.1093/ajh/hpz116
4. Gkaliagkousi E, Nikolaidou B, Gavriilaki E, et al. Increased erythrocyte- and platelet-derived microvesicles in newly diagnosed type 2 diabetes mellitus. *Diabetes Vasc Dis Res*. 2019;16(5):458–465. https://doi.org/10.1177/1479164119844691

### P.6

### Males with abdominal aortic aneurysm have reduced left ventricle function

**Mrs Ida Åström Malm**[1], Dr Rachel De Basso[1], Professor Jan Engvall[2,3], Dr Peter Blomstrand[1,4]
[1]Department of Natural Sciences and Biomedicine, School of Health and Welfare, Jönköping University, Jönköping, Sweden, [2]Department of Clinical Physiology and Department of Health, Medicine and Caring Sciences, Linköping University, Linköping, Sweden, [3]Center for Medical Image Science and Visualization, Linköping University, Linköping, Sweden, [4]Department of Clinical Physiology, County Hospital Ryhov, Jönköping, Sweden

**Introduction:** Abdominal aortic aneurysm (AAA) is a common disease in the ageing population with a prevalence of approximately 1.5–3% of males aged 65–70 years in Europe (1, 2). AAA is mostly an asymptomatic disease, however, if the AAA ruptures it is a life-threatening event. Even without a rupture, AAA increases the risk of developing chronic heart failure and other major cardiovascular events.

**Purpose**: Even though AAA may lead to heart failure, no study has previously systematically explored the left ventricular function in patients with AAA. Thus, the aim of this echocardiographic study is to investigate whether AAA is associated with left ventricular dysfunction.
**Methods:** Echocardiography was performed in 307 males (199 AAA and 108 controls) aged 55–80 years. The males were recruited from a regional ultrasound surveillance program of known AAA or an ongoing ultrasound screening program during 2011–2016.
**Results:** Males with AAA had a 2.2% lower left ventricular ejection fraction and 1% lower global longitudinal strain compared to control. They had also a significantly thicker septal and posterior wall and a higher E/e'. Ischemic heart disease was reported eight times, hypertension and smoking four times more frequently in participants with AAA compared to controls.
**Conclusion:** While hypertension, smoking and ischemic heart disease are far more common in males with AAA than controls the difference in left ventricular function is only mild. Subjects with AAA have a slight impaired left ventricular systolic and diastolic function.

**References**

1. Svensjo S, Bjorck M, Gurtelschmid M, Djavani Gidlund K, Hellberg A, Wanhainen A. Low prevalence of abdominal aortic aneurysm among 65-year-old Swedish men indicates a change in the epidemiology of the disease. Circulation. 2011;124(10):1118–23.
2. Lederle FA, Johnson GR, Wilson SE, Chute EP, Littooy FN, Bandyk D, et al. Prevalence and associations of abdominal aortic aneurysm detected through screening. Aneurysm Detection and Management (ADAM) Veterans Affairs Cooperative Study Group. Ann Intern Med. 1997;126(6):441–9.

### P.7

### Heart rate modulates the relationship of augmented systolic blood pressure with the blood natriuretic peptide levels

**Professor Hirofumi Tomiyama**[1]
[1]Tokyo Medical University

**Aims:** The present study examined whether the association of the Central systolic blood pressure (cSBP) with the the serum N-terminal fragment B-type natriuretic peptide (NT-proBNP) levels might differ between subjects with high and low HRs.

**Methods**: In 2257 middle-aged healthy Japanese men, serum NT-proBNP levels, radial augmentation index (radial AI), and the first and second peaks of the peripheral systolic blood pressure (SP1 and SP2, markers of the cSBP) were measured.
**Results**: The serum NT-proBNP levels among three groups classified by the HR (i.e., $\leq 69$, 70–79, and $\geq 80$). While the serum NT-proBNP levels were similar among the three groups, the radial AI increased and the SBP1-2 (SBP1 minus SBP2, i.e., a low SBP1-2 corresponds to augmentation of the cSBP relative to the bSBP) decreased significantly with decreasing HR. In multivariate linear regression analyses, the SBP2 showed a significant association with the serum NT-proBNP levels in the overall study population. However, in subgroup analyses, the SBP2 showed a significant association with the serum NT-proBNP levels only in subjects with HR $\leq 69$ beats/minute.
**Conclusion:** In middle-aged Japanese men, the relationship between the cSBP and the cardiac afterload appears to differ depending on the heart rate; the results of our analysis showed that the relationship between the cSBP and cardiac overload may be more pronounced and strongly significant in patients with low HRs as compared to patients with high HRs.

### P.8

### Carotid enlargement is associated with the presence and severity of coronary artery disease assessed by Gensini Score in patients submitted to coronary angiography

Professor Luiz Bortolotto[1], **Dra Nadja Mendes**[1], Ms Valeria Costa-Hong[1], Dr Stefano Garzon[1], Professor Pedro Lemos[1]
[1]Instituto Do Coração, São Paulo, Brazil

**We** evaluated the association of carotid measurements (intima-media thickness-cIMT, internal diameter and distensibility) obtained by radio-frequency ultrasound (Wall Track System®) with the presence and severity of coronary artery disease (CAD) assessed by Gensini Score in 69 patients prospectively submitted to coronary angiography due clinical suspicion of CAD. The presence of CAD was considered when the Gensini score $\geq 1$. The mean age was $58.4 \pm 8$ years, 50% men, 85% arterial hypertension, 37% diabetes and 40% smoking. The presence of CAD was observed in 44 patients (63%), with a higher proportion of men in patients with CAD (59% vs. 28%, $p < 0.05$). Patients with CAD had a greater internal diameter of carotid artery [7758.2 (7096.1; 8797.2) µm vs. 7009.0 (6595.5; 7396.0) µm, $p = 0.003$), while it was not observed differences in cIMT or distensibility. In logistic regression analysis, the chance of presenting CAD by Gensini score increases by 6.7% at each 100 µm increase in the internal diameter regardless of gender. A significant correlation was observed between the carotid diameter and Gensini score ($r = 0.289$, $p = 0.02$). Among carotid artery measurements obtained by radio-frequency ultrasound, the internal diameter was the only significantly associated with the presence and severity of CAD estimated by Gensini score.

### P.9

### A novel ultrasound-based method for heart failure screening

**Albert Chang**[1], Dhruv Reddy[1], Shashank Adapa[1], Anenta Ratneswaren [1,2], Ryan Reavette[2], Peter Weinberg[2]
[1]Faculty of Medicine, [2]Department of Bioengineering, Imperial College London

**Background:** Heart failure (HF) is prevalent and, although treatable, has a one-year prognosis of 40%. Moreover, 80% of diagnoses in England are made after a hospital admission despite 40% of patients visiting their GP with symptoms beforehand (1). The most common symptoms – breathlessness, fatigue and ankle swelling – are nonspecific, so patients are not always referred for echocardiography, the gold-standard for diagnosis. This motivates improved HF screening in Primary Care.

Studies have shown that arterial wave intensity is altered in HF (2, 3), but the technique relies on measuring pressure and velocity waveforms simultaneously; current methods are invasive, inaccurate or cumbersome. We propose a noninvasive method that uses ultrafast ultrasound to measure diameter and velocity instead; both variables can be accurately measured with one scan but give similar clinical indications.

**Methods:** Patients undergoing echocardiography were recruited from outpatient Cardiology clinics, and B-mode ultrasound scans were taken of their left carotid, right carotid and left brachial arteries. Blood signal was enhanced using spatiotemporal filtering. Velocity and diameter waveforms were obtained using cross-correlation-based techniques.

**Results:** Wave intensities have been computed for 100 patients. There is a significant difference ($p < 0.05$) in the intensities of waves that represent systolic contraction and peripheral reflection between patients with HF with reduced ejection fraction and those without symptoms of HF or reduced ejection fraction.

**Conclusions:** Noninvasive wave intensity analysis has potential to improve HF screening in Primary Care.

We gratefully acknowledge support from BHF, Imperial College BHF Centre of Research Excellence and EPSRC Impact Acceleration Award.

### P.10

### A comparison of aortic haemodynamic parameters between the SphygmoCor CvMS (radial tonometry) device and the PULSE (brachial oscillometry) device

**Mr James Cox**[1], Dr Ahmad Qasem[2], Dr Isabella Tan[1], Emeritus Professor Alberto P. Avolio[1], Dr Mark Butlin[1]

[1]Macquarie University, Sydney, Australia, [2]CardieX-AtCor, Sydney, Australia

**Background:** Current cardiovascular outcomes are based primarily on the measurement of brachial arterial blood pressure (BP). Whilst this measurement is acceptable, aortic haemodynamic parameters offer a more representative assessment of an individual's cardiac load. Currently, there is a lack in devices assessing aortic parameters.

**Objective(s):** To compare aortic haemodynamic parameters between the previously validated SphygmoCor CvMS device and the new consumer PULSE device.

**Methods:** Sequential randomised measurements were performed in triplicate in a seated position with the SphygmoCor CvMS device (radial tonometry) and the PULSE device (brachial oscillometry). A subset of individuals underwent an isometric handgrip exercise to compare measured haemodynamic parameters under conditions of elevated BP. Aortic parameters were generated by applying a transfer function to the radial and brachial waveforms, respectively, and compared using Bland–Altman plots and mean differences.

**Results:** Participants' ($n = 41$, 20 female, age: $44 \pm 19$ years) seated baseline oscillometric brachial systolic and diastolic BP were $122 \pm 15$ and $76 \pm 11$ mmHg, respectively. Baseline differences for all aortic pressure parameters (systolic BP, diastolic BP, mean BP, pulse pressure, augmentation pressure) were equal to or less than $1.4 \pm 2.8$ mmHg. The difference in augmentation index between the devices was $0.1 \pm 7.3\%$. Following a significant increase in BP ($p < 0.01$) from the isometric handgrip exercise, aortic pressure parameters were equal to or less than $0.9 \pm 3.7$ mmHg, whilst the difference in augmentation index between the devices was $-1.5 \pm 6.0\%$.

**Conclusions:** Aortic haemodynamic parameters measured by the brachial ocillometry PULSE device were comparable to those measured in the radial tonometry CvMS system under baseline and exercise conditions.

### P.11

### Poor cardiovascular health is associated with high body fat and sympathetic tone in obese subjects

**Mrs Michelle Cunha**[1], Mrs Samanta Mattos[1], Mrs Thayná Brum[1], Mrs Marcia Klein[1], Mr Mario Neves[1]

[1]State University of Rio de Janeiro, Rio de Janeiro, Brazil

**Background:** According to the American Heart Association, seven behaviors and risk factors define metrics of cardiovascular health, classifying it as ideal, intermediate (iCVH) or poor (pCVH)[1]

**Objective:** To evaluate body adiposity and sympathetic tone in obese individuals with different cardiovascular health classifications.

**Methods:** Cross-sectional study with patients aged 40–70 years and body mass index (BMI) $\geq 30$ and $< 40$ kg/m$^2$, submitted to sympathetic tone assessment by heart rate variability and body composition by Dual X-Ray Absorptiometry.2 Patients ($n = 57$) were divided into two groups: iCVH ($n = 32$) and pCVH ($n = 25$).

**Results:** The patients were predominantly females in both groups (66 vs 88%, $p = 0.067$) with similar age ($52 \pm 7$ vs $51 \pm 6$ years, $p = 0.648$). Systolic and diastolic blood pressure presented higher values in pCVH group ($120 \pm 15/76 \pm 10$ vs $129 \pm 13/83 \pm 8$ mmHg; $p = 0.026/p = 0.06$), and no statistical significance in cardiovascular risk ($9.6 \pm 8.8$ vs $9.0 \pm 6.5$ years, $p = 0.787$). Similar results were also observed in BMI ($34.2 \pm 2.7$ vs $34.0 \pm 2.8$ kg/m$^2$, $p = 0.784$) and waist-hip ratio ($0.86 \pm 0.07$ vs $0.84 \pm 0.06$ cm, $p = 0.306$). Total body fat ($40 \pm 6$ vs $48 \pm 6\%$, $p = 0.004$) and trunk fat ($45 \pm 6$ vs $53 \pm 4\%$, $p = 0.008$) were significantly higher in pCVH. There were significant differences in Sympathetic Nervous System Index (iSNS) ($0.39 \pm 1.05$ vs $1.20 \pm 1.22$, $p = 0.050$), Parasympathetic (iPNS) ($0.13 \pm 0.92$ vs $-0.39 \pm 0.85$, $p = 0.037$), heart rate ($62 \pm 7$ vs $68 \pm 8$ bpm, $p = 0.019$), and no significance but higher values in Low Frequency-High Frequency ratio ($1.06 \pm 0.94$ vs $1.65 \pm 2.24$, $p = 0.195$) in pCVH group.

**Conclusion:** In these obese subjects BMI was not useful to assess cardiovascular risk, and poor CVH was associated with higher sympathetic tone and fat percentage in body composition evaluation.

### P.12

### Moderate to severe obstructive sleep apnea associated with early vascular aging and sympathetic hyperactivity in obese individuals

**Ms Samanta Mattos**[1], Ms Michelle Cunha[1], Medical Student Larissa Silva[1], MD PhD Márcia Klein[1], MD PhD Mario Neves[1]

[1]State University Of Rio De Janeiro, Rio De Janeiro, Brazil

**Introduction:** Obstructive sleep apnea (OSA) and obesity may have synergistic effects on the progress of cardiovascular disease.

**Objective:** To evaluate sympathetic tone and vascular disease in obese patients with moderate and severe OSA.

**Methods:** Individuals of both sexes, aged 40–70 years and body mass index (BMI) $\geq 30$ and $< 40$ kg/m$^2$, submitted to assessment of heart rate variability (HRV), endothelial function by flow-mediated dilatation (FMD), central parameters by Mobil-O-Graph and carotid ultrasound. The sleep study was performed through a portable home sleep test device (WatchPAT).

**Results:** Patients ($n = 61$) were divided by the apnea–hypopnea index (AHI): mild-absent (MA) group (AHI $< 15$, $n = 30$) and moderate-severe (MS) group (AHI $\geq 15$, $n = 31$). There was no significant difference in age ($50 \pm 6$ vs $54 \pm 8$ years) and BMI ($35 \pm 3$ vs $34 \pm 2$ kg/m$^2$). MS group presented higher neck circumference ($38 \pm 3$ vs $40 \pm 4$ cm, $p = 0.025$), waist-hip ratio ($0.83 \pm 0.07$ vs $0.88 \pm 0.06$ cm, $p = 0.005$), systolic blood pressure ($120 \pm 13$ vs $129 \pm 15$ mmHg, 0.017), pulse pressure ($42 \pm 9$ vs $48 \pm 9$ mmHg, $p = 0.007$), cardiovascular risk ($6.4 \pm 3.8$ vs $11.9 \pm 9.5\%$, $p = 0.012$). As expected, the oxygen desaturation index ($2.5 \pm 2.4$ vs $20.2 \pm 14.5$ events/h, $p = < 0.001$) was higher in the MS group, which also had lower FMD ($9.9 \pm 6.3$ vs $6.9 \pm 4.3\%$, $p = 0.032$). The low/high frequency ratio ($0.85 \pm 0.53$ vs $1.72 \pm 2.07$ms$^2$, $p = 0.046$), pulse wave velocity ($7.0 \pm 0.7$ vs $7.8 \pm 1.2$ m/s, $p = 0.004$), vascular age ($48 \pm 7$ vs $54 \pm 8$ years, $p = 0.05$), mean intimal-media thickness (IMT) ($0.59 \pm 0.10$

vs $0.67 \pm 0.12$ mm, $p = 0.008$) and maximum IMT ($0.64 \pm 0.12$ vs $0.72 \pm 0.14$ mm, $p = 0.023$) were significantly higher in the MS group.
**Conclusion:** In this sample of obese individuals, moderate to severe OSA was associated with sympathetic hyperactivity, endothelial dysfunction and arterial stiffness.

### P.14

### Loss of stearoyl-CoA desaturase 1 induces inflammation and arterial wall remodelling

**Dr Anna Filip**[1], Dr hab Pawel Dbrzyn[1]
[1]Nencki Institute of Experimental Biology, Polish Academy of Sciences, Warsaw, Poland, 3 Pasteur Street, Poland

**The** role of stearoyl-CoA desaturase 1 (SCD1) in vascular smooth muscle cells (VSMC) has been studied in the context of vascular dysfunction development. The SCD1 and SCD2 control a process of calcification and regulate lipotoxicity in VSMC. Moreover, the role of SCD1 in lipid accumulation in VSMC and foam cell formation from VSMC was recently revealed [1- 3]. Therefore, the present study aimed to identify the role of SCD1 in VSMC phenotypic and metabolic alteration and the development of vascular dysfunction associated with inflammation. Using the $SCD1^{-/-}$ mouse model, fed chow or high-fat diet (HFD), we have studied (1) inflammation development in blood and aorta, (2) immune cell infiltration toward the aortic wall, (3) VSMC phenotypic alteration, and (4) the regulation of main cellular metabolic pathways. Obtained results show that (a) SCD1 expression is down-regulated in the aorta in WT mice fed HFD; (b) loss of SCD1 expression induced the inflammation in the blood and aortic wall; (c) $SCD1^{-/-}$ mice presented increased recruitment of immune cells toward the aorta; (d) SCD1 deficiency affects the VSMC dedifferentiation; (e) SCD1 regulates cell cycle and PDGF signaling pathway in VSMC in mice fed chow or HFD. Taken together, our data show that signaling pathways controlled by SCD1 represent an important step in the regulation of VSMC phenotype thus vascular wall morphology and function.

This work has been supported by the funding from National Science Centre, Poland grants no UMO-2017/26/D/NZ4/00696 and UMO-2016/22/E/NZ4/00650).

### P.15

### Skin autofluorescence and serum biomarkers of glucose metabolism: which parameters contribute most to aortic stiffness?

**Professor Jan Filipovský**[1], Professor Otto Mayer, Associate Professor Jitka Seidlerová, Doctor Július Gelžinský
[1]Charles University Medical Faculty Pilsen, Czech Republic, Pilsen, Czech Republic

**Background:** Impaired glucose metabolism, leading to the deposition of advanced glycation end products (AGE), plays an important role in the stiffening of large arteries. This process is counterbalanced by circulating soluble receptor for AGE (sRAGE).

**Methods:** We investigated the association of arterial stiffness with several biomarkers and with the degree of skin deposition of AGE in a sample of 867 subjects from general population, examined in the Czech post-MONICA study. Carotid-femoral pulse wave velocity (PWV) was measured by SphygmoCor device (AtCor Medical Ltd.) and skin AGE were measured using a dedicated autofluorescence method (AGE Reader mu®); the method is quick and easy to use. To quantify the circulating status of AGE, carboxymethyl lysine (CML) was assessed by ELISA; sRAGE concentrations were also assessed by ELISA.
**Results:** When analyzing the whole sample using both multiple linear and logistic regression models and after adjustment for potential covariates, significant associations of PWV with fasting glycemia, HbA1c, sRAGE, skin AGEs, and the skin AGE- to-sRAGE ratio were found. Stepwise models identified strong association with the skin AGE and the ratio of skin AGE-to- sRAGE, independently of serum glucose level, age and mean arterial pressure; this was also true when diabetic subjects were excluded. In contrast, neither CML, nor its ratio to sRAGE showed any association with arterial stiffness.
**Conclusion:** The amount of skin AGE and sRAGE, which prevents deposition of AGE into tissues, are important factors contributing to aortic stiffness over and above serum glucose level.

### P.16

### Central arterial pressure changes during and after head-down tilt bedrest.

**Dr Catherine Fortier**[1], Dr Antoine Fayol[2], Dr Hakim Khettab[1,2], Dr Rosa-Maria Bruno[1,2], Professor Pierre Boutouyrie[1,2]
[1]INSERM U970, Paris Cardiovascular Research Center (PARCC), Cellular molecular and physiological mechanisms of heart failure (Team 7), Paris, France, [2]AP-HP, Pharmacology Unit, Hôpital Européen Georges Pompidou, Université de Paris, Paris, France

**Background/objectives:** Head-down tilt bed rest (HDTBR) induces a redistribution of blood volume from lower limbs to the upper and central parts of the body, which mimics physiological changes during long-term exposure to microgravity. However, the effects of HDTBR on central arterial pressure parameters during bedrest and after the return to verticality remain poorly described.

**Methods:** 20 healthy men (age: $34 \pm 8$ years) lay supine on a 6-degrees declined bed for 60 days. Brachial blood pressure measurements and central arterial pressure obtained from carotid arterial tonometry were measured before (B0), during bed rest (H52), and after 30 days of recovery (R30). Pairwise comparisons between times of measurement were assessed using mixed models for repeated measures with Bonferroni correction.
**Results:** From B0 to H52, central systolic pressure (cSP) and central diastolic pressure (cDP) increased to a similar extent, without reaching significance (ΔcSP: 5.8 mmHg, [95% CI − 1.6; 13.2]; ΔcDP: 6.0 mmHg, [95% CI − 0.9; 12.9]). AIx@75 ($-11 \pm 10\%$ vs. $1 \pm 11\%$, $p = 0.005$) and end-systolic pressure (ESP: $84 \pm 9$ mmHg to $92 \pm 9$ mmHg, $p = 0.012$) increased, while P1 amplitude did not change ($29 \pm 8$ mmHg vs. $27 \pm 5$ mmHg, $p = 0.980$). At R30, cSP was significantly higher compared to baseline ($104 \pm 8$ mmHg vs. $95 \pm 9$ mmHg, $p = 0.002$), but not cDP ($71 \pm 9$ mmHg vs. $66 \pm 10$ mmHg, $p = 0.093$). At R30, AIx@75 ($-4 \pm 11\%$) and ESP ($92 \pm 9$ mmHg) did not recover back to baseline values (both $p < 0.05$).
**Conclusions:** This study suggest that HDTBR leads essentially to an increased afterload which is not fully corrected after 30 days of recovery.

### P.17

### Radial-digital pulse wave velocity: response of small peripheral arteries to nitroglycerin

**Charles-antoine Garneau**[1], Catherine Fortier[1,2], Hasan Obeid[1], Mathilde Paré[1], Karine Duval[1], Dr. Mohsen Agharazii[1,3]
[1]CHU de Québec Research Center- Hôtel-Dieu de Québec Hospital, Québec, Canada, [2]Paris Cardiovascular Research Center, INSERM U970, Paris, France, [3]Division of Nephrology, Department of Medicine, Faculty of Medicine, Université Laval, Québec, Canada

**Objectives:** We have already demonstrated that nitroglycerin (NTG) induces different regional stiffness response from elastic and muscular arteries, predisposing small peripheral arteries to increased pulsatility1. Peripheral smaller arteries may also respond differently to nitroglycerin. The objective of this study is to compare the changes in radial-digital pulse wave velocity (RD-PWV) following administration of NTG from healthy individuals to those with chronic kidney disease (CKD).

**Method:** This study was conducted among 36 participants, 27 healthy (56% women, age 48.4 years) and 9 CKD (33% women, age 70.3 years). Piezoelectric sensors (Complior, France) were placed on the carotid and radial arteries, as well as on the tip of the index finger to obtain the carotid-radial PWV (CR-PWV) and the RD-PWV. These measurements were repeated 4 min after sublingual administration of 0.4 mg NTG.
**Results:** At baseline, the CKD group had a significantly lower RD-PWV than the healthy group ($3.27 \pm 1.38$ m/s vs. $4.72 \pm 1.20$ m/s, $p = 0.008$). After NTG administration, the RD-PWV of the healthy and CKD groups both significantly increased to reach $6.09 \pm 2.23$ m/s and $4.69 \pm 1.87$ m/s, respectively ($p < 0, 01$). However, in both populations, the stiffness of the entire upper limb (carotid-digital PWV) did not change significantly ($p = 0.816$ and $p = 0.169$).
**Conclusions:** Again, we observed opposite changes in the stiffness of different vascular territories with vasodilator drug resulting in no change in stiffness for the entire upper limb. These are preliminary results, but this new technique may open up the path for a better understanding of the consequences of a modified stiffness gradient on the microcirculation.

### P.18

### Are arteries designed to minimise variation in arterial pressure of the blood volume stored during the systole?

**Ph.d. Benjamin Gavish**[1]
[1]Yazmonit Ltd., Jerusalem, Israel
**Background:** Arterial compliance enables arteries to store blood during the systole ('pulse volume'). This temporary storage plays the important role of buffering arterial pressure pulsatility. Arterial compliance varies with pressure. Here, we investigate the pressure dependence of pulse volume using a model approach.

**Methods:** Expression of the pulse volume using two phenomena observed in individuals (figure): i) exponential relationship between arterial pressure (P) and arterial volume (or cross-sectional area) V, in normal and high pressures given by $P = \alpha + \exp(V)$, where α, and are pressure-independent, and ii) linear relationship of repeated measurements of systolic blood pressure SBP and diastolic blood pressure DBP, i.e., $SBP = A + K\,DBP$, where A and K are constants.
**Results:** Taking Vs and Vd to be the arterial blood volumes corresponding to SBP and DBP, respectively, it is straightforward to show that $K = \exp[(Vs\text{-}Vd)]$, where Vs-Vd is the pulse volume. Since K andare constants, this model shows that pulse volume is independent of blood pressure.
**Conclusions:** The model supports the view that the pressure–volume relationship in arteries may play a role in regulating blood volume stored by arteries during the systole.

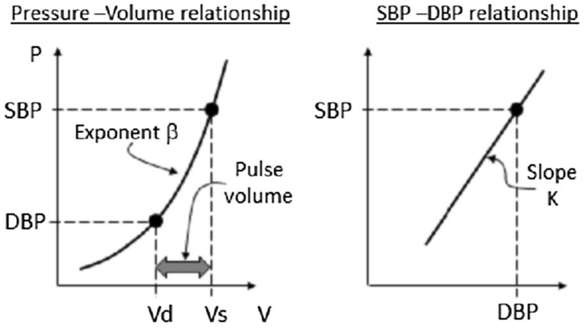

### P.19

### Comparison between invasive and noninvasive methods to determine subendocardial oxygen supply and demand imbalance from aortic pressure waveform

**Dr. Andrea Grillo**[1,8], Dr. Filippo Scalise[3], Dr. Lucia Salvi[4], Dr. Isabella Tan[5], Dr. Lorenzo De Censi[6], Dr. Antonio Sorropago[3], Dr. Giovanni Sorropago[6], Dr. Francesco Moretti[7], Dr. Matteo Rovina[1], Prof. Bruno Fabris[8], Prof. Renzo Carretta[8], Prof. Alberto Avolio[5], Prof. Gianfranco Parati[2,6], Prof. Paolo Salvi[2]
[1]Medicina Clinica, Azienda Sanitaria Universitaria Giuliano Isontina, Trieste, Italy, [2]IRCCS Istituto Auxologico Italiano, Milano, Italy, [3]Department of Interventional Cardiology, Policlinico di Monza, Monza, Italy, [4]Arcispedale S. Maria Nuova, Cardiovascular Medicine, Reggio Emilia, Italy, [5]Macquarie University, Department of Biomedical Sciences, Faculty of Medicine and Health Science, Sydney, Australia, [6]Department of Medicine and Surgery, University of Milano-Bicocca, Milano, Italy, [7]Policlinico San Matteo Foundation, University of Pavia, Department of Molecular Medicine, Pavia, Italy,[8]Department of Medical, Surgical and Health Sciences, University of Trieste, Trieste, Italy
**Background:** Evaluation of the balance between subendocardial oxygen supply and demand could be a useful parameter to assess the risk of myocardial ischemia. The subendocardial viability ratio (SEVR, also known as Buckberg index) determined by invasive recording of left ventricular and aortic pressure curves is a valid method to estimate the degree of myocardial perfusion relative to left ventricular workload. Arterial applanation tonometry allows a non-invasive estimation of SEVR as the ratio of the areas directly beneath the central aortic pressure curves obtained during diastole (myocardial oxygen supply) and during systole (myocardial oxygen demand). However, "traditional" non-invasive SEVR is affected by limitations that make it significantly different from the invasive SEVR.

**Methods:** We have developed a new method for evaluation of SEVR with carotid tonometry by taking into account the intra- ventricular diastolic pressure and proper allocation to systole and diastole of left ventricular isometric contraction and relaxation. SEVR values estimated with carotid tonometry (PulsePen, DiaTecne, Italy) by "traditional" and "new" method were compared with those evaluated invasively by cardiac catheterization.
**Results:** The "traditional" method provided significantly higher SEVR values than the reference invasive SEVR: average of differences $\pm$ SD $= 0.44 \pm 0.11$ (limits of agreement: 0.23–0.65). The non-invasive "new" method showed a much better agreement with the invasive determination of SEVR: average of differences $\pm$ SD $= 0.00 \pm 0.08$ (limits of agreement: $-$ 0.15– 0.16).
Conclusions: Carotid applanation tonometry provides valid non-invasive SEVR values only when all the main factors determining myocardial supply and demand flow are considered.

### P.20

### Healthy young men show a larger response in carotid artery dilation during a cold pressor test compared to age- matched females

**dr. Yvonne Hartman**[1], Daniek Dinnissen[1], Prof. dr. Dick Thijssen[1,2]
[1]Radboudumc, Nijmegen, Netherlands, [2]Liverpool John Moores University, Liverpool, United Kingdom
**Background:** The carotid artery reactivity (CAR) test was recently identified as an early marker of conduit artery vascular health. This study examined potential sex differences in CAR between healthy young men and women.

**Methods:** In this preliminary analysis on data of the ongoing Healthy Brain study (NTR7955), 245 participants (113 males) aged 30–39 were included. Ultrasound was used to measure common carotid artery diameter. After 1 min baseline measurements, a hand was immersed in ice water for 3 min. CAR% was defined as maximal diameter change during cold pressor test compared to baseline. After 4 months, measurements were repeated in a subgroup ($n = 86$, 45 males)). CAR% was compared between males and females, and whether these potential differences remain present across time.
**Results:** Our cross-sectional data revealed a larger CAR% in males compared to females ($3.1 \pm 3.3$ vs $2.2 \pm 3.3\%$, $P = 0.043$). Males reported a significantly larger baseline diameter ($6.9 \pm 0.6$ mm vs $6.3 \pm 0.6$ mm, $P < 0.001$). Correcting the CAR% for between group differences in baseline diameter reinforced the larger CAR%. Although CAR% increased from baseline to 4 months ($2.5 \pm 3.5$ to $3.4 \pm 2.7\%$, $P = 0.027$), the increase in CAR% did not differ between males and females.
**Conclusions:** Young, healthy men show a consistently higher CAR% than females. This suggests that young healthy men have better carotid artery vascular health compared to age-matched women. Future studies are required to better understand how this difference in vascular health may translate to development of cardiovascular disease.
This trial is registered at https://www.trialregister.nl/trial/7955.

### P.21

### Longitudinal clinical trajectory analysis of individuals before and after diagnosis of Type 2 Diabetes Mellitus (T2DM) indicates that vascular problems start early

**Dr Adrian Heald**[1], Professor Simon George Anderson, Professor Yonghong Peng, Professor Martin Gibson, Dr Helene Fachim, Professor Bill Ollier
[1]University of Manchester, Salford, United Kingdom

**Introduction:** Type 2 diabetes mellitus (T2DM) represents a major/increasing chronic health burden at a population level. T2DM frequently associates with increasing multi-morbidity/treatment complexity[1]. Longitudinal clinical histories of individuals both before and after diagnosis of T2DM are likely to provide additional insight into its aetiology and consequences.

**Methods:** This study utilised diabetes patients and controls enrolled in the DARE (Diabetes Alliance for Research in England). Longitudinal data of 281 individuals (T2DM n = 237 and matched non-T2DM controls n = 44) were extracted. Trajectory Analysis over a period of up to 70 years based on calculations of the proportions of most prominent clinical conditions for each year.

**Results:** For individuals who eventually had a diagnosis of T2DM made, a number of clinical phenotypes were seen to increase consistently in the years leading up to diagnosis of T2DM. Of these documented phenotypes, the most striking were diagnosed hypertension (more than in the control group) and asthma. This trajectory over time was much less dramatic in the matched control group.

Immediately prior to T2DM diagnosis a greater indication of ischaemic heart disease proportions was observed. Post-T2DM diagnosis, the proportions of T2DM patients exhibiting hypertension and infection continued to climb rapidly before plateauing. Ischaemic heart disease continued to increase in this group as well as retinopathy, impaired renal function and heart failure.

**Conclusion:** These observations provide an intriguing and novel insight into the onset and natural progression of T2DM. They suggest an early phase of potentially-related disease activity well before any clinical diagnosis of diabetes is made.

### P.22

### A longitudinal pilot study of pulse wave velocity in female adolescents with severe anorexia nervosa

**Dr Lee Hudson**[1], Mr Daniel Jacobs[1], Dr Hind Al-Khairulla[1], Ms. Alicia Rapala[1], Professor Russell Viner[1], Dr Dasha Nicholls[1], Professor Alun D Hughes[1]
[1]Gos Ucl Institute of Child Health, London, United Kingdom

**Background:** Anorexia Nervosa (AN) is a mental disorder with well-established acute cardiovascular complications. Less is known about the impact on arterial stiffening and there are no published longitudinal studies. Here we present interim data from a pilot longitudinal study of Pulse Wave Velocity (PWV) in underweight young adolescents with AN admitted to an eating disorder unit in the United Kingdom.

**Methods:** We measured carotid-femoral PWV in all new admissions to a single eating disorder unit from December 2020 who met inclusion criteria: 1) diagnosis of AN; 2) aged 12–18 years; 3) underweight (< 85% of average BMI for age and sex). PWV was measured using Vicorder by a single operator at admission and weekly for 12 weeks. Ethics approval was provided by a London ethics committee. Standardised PWV Z-score for age (PWVz) was derived from published data.

**Results:** For 7 participants, baseline mean PWV was 7.19 (SD 0.40) m/s. Mean PWVz was 3.81 (SD 0.71, one sample t-test $p < 0.01$). Mean BMI was 15.62 (0.65 SD) $kg/m^2$. Baseline PWV and PWVz were not associated with baseline BMI. In multi-level, mixed effects models PWV and PWVz decreased over time in weeks (coefficient − 0.05, 95% CI − 0.07 to 0.03; coefficient −0.10, 95% CI − 0.5 to − 0.05 respectively).

**Conclusions:** We have demonstrated feasibility in collecting repeated PWV measures in this patient group. Interim findings suggest high standardised scores of PWV compared to population normative data, and temporal decreases in PWV and PWV z-score during admission. Further research is needed on long term patterns of PWV in AN patients, and to understand possible underlying mechanisms.

### P.23

### Dynamic time warping for measuring incremental pulse wave velocity: demonstration on a porcine model

Mr. V Raj[1], Dr. P M Nabeel[2], **Dr. Jayaraj Joseph**[1]
[1]Indian Institute Of Technology Madras, Chennai, India, [2]Healthcare Technology Innovation Centre, Indian Institute of Technology Madras, Chennai, India

**Background:** While outlining the clinical importance of incremental pulse wave velocity (PWV) (1), attempts were made to measure it by simultaneously acquiring pressure and diameter from a single arterial site. Owing to the methodological limitations of such methods (1,2), especially when applied to central arteries, we present a novel dynamic time-warping- based technique to measure incremental PWV from a pair of identical blood pulse signals captured from two proximal sites.

**Methods:** Its functionality was demonstrated on an anesthetized swine during baseline condition and infusion of vasoconstritive drugs (elevating BP). A pair of pressure waveforms (sampling rate = 20 kHz) was captured from the left common carotid artery using a dual-element pressure catheter (5F, sensor spacing = 30 mm, Millar SPR-751, ADInstruments, India), from which beat-to-beat end-diastolic ($C_D$) and peak-systolic PWVs ($C_S$) were measured.

**Results:** Baseline $C_D$ and $C_S$ evaluated from the captured high-resolution pressure waveforms were repeatable (variability < 4.5%). Both measures were strongly associated ($r > 0.88$, $p < 0.01$) with pressure and increased (> 2.5 times) with the increment in mean pressure (~ 3 times) induced by the drug. $C_S$ measured significantly greater ($p < 0.0001$) than $C_D$, 6.1 m/s versus 5.4 m/s during baseline (82/55 mmHg) and 18.9 m/s versus 12.8 m/s during peak-drug-effect (226/184 mmHg).

**Conclusions:** The method reliably measured the incremental nature of PWV and its inter-beat dependence on blood pressure, invasively. Development of an ultrafast-fast ultrasound employing this method is underway, catering the need for a non-invasive technology that's amenable for clinical settings.

### P.24

### Changes of fingertip photopletysmography derived parameters during acute SARS-CoV-19 infection in two patients with daily monitoring

Dr. Kulin Dániel[1,2], Dr. Zsuzsanna Miklós[1], Dr. Sandor Kulin[1]
[1]Institute of Translational Medicine, Semmelweis University, Budapest, Hungary, [2]E-Med4All Europe Ltd., Budapest, Hungary

**Purpose/background/objectives:** SARS-CoV-2 infection has various effect on the cardiovascular system above respiratory symptoms. (1,2) Peripheral pulsewave morphology alterations might reflect these changes. Our aim is to showcase two patients to demonstrate how these effects can be captured with a remote patient monitor system with daily recordings and to compare the changes of the different parameters to start a scientific discussion about the possible clinical utility of such monitoring in COVID-19 patients.

**Methods:** Signals were recorded with a remote patient monitor system. (SCN4ALL, E-Med4All Europe Ltd., Hungary) (3) Patients were conducting measurements at rest, placing the device at one of their index fingers.

All the parameters collected in the different clinical conditions (pre-;in-;post-COVID) were displayed with a box-plot method.

**Results:** Both subjects had elevated heart rate and decreased oxygen saturation during the acute phase of the disease. There are parameters showing the same patterns during the acute phase (systolic slope inclination, b/a, early left ventricular ejection time1 and 2, ejection time and crest-time). However, there are certain other parameters with discrepancies between subjects. (Stiffness index, reflection index, d/a, dicrotic notch index, Ageing index, Left ventricular ejection time index).

Pulse rate variability time domain parameters (SDNN and RMSSD) both exhibited decreased values, as sign of ongoing infection (4).

**Conclusions:** This case presentation demonstrates certain pulsewave analysis based cardiovascular parameter changes during COVID- 19 disease. Well-designed studies are needed to unfold the potential of daily pulsewave monitoring in the outcome prediction, phenotyping and the personalized therapy of COVID-19 and other diseases**.**

### P.25

### The relationship between intima-media thickness and global longitudinal strain value measured by 2D-strain ultrasound in obese patients

**Angela Cozma**[1,2], Andrada-Luciana Lazar[1,3], Benjamin Guilherme Rodrigues[1], Gaétan Masson[1], Adela Sitar[1,2], Olga Orasan[1,2], Adriana Fodor[1,4], Vasile Negrean[1,2]

[1]"Iuliu Haţieganu" University Of Medicine And Pharmacy, Cluj-Napoca, Romania, [2]4th Medical Department, Cluj-Napoca, Romania, [3]Dermatology Department, Cluj-Napoca, Romania, [4]Department of Diabetes and Nutrtion, Cluj-Napoca, Romania

**Background:** There is a strong correlation between intima-media thickness **(**IMT) and several cardiovascular risk factors, and more importantly with clinical and subclinical atherosclerosis (1). Additionally, IMT assessment represents a valuable tool in order to anticipate the individual risk of coronary artery disease (2) and impaired left heart function (3). The aim of our study was to evaluate the IMT and global longitudinal strain (GLS) values in obese and overweight patients, compared to normal-weight patients, and to determine a potential relationship between these measurements and the patient's body mass index (BMI).

**Methods:** A total of 31 patients were included in our study. The subjects were assigned to 3 groups as it follows: "obesity" group, "overweight" group and "normal weight" group. The assessment of left ventricle systolic function was performed by 2D- strain echocardiography. IMT measurements were also performed.

**Results:** 31 patients were included in our study, 8 (25.81%) had normal weight, 12 (38.71%) were overweight and 11 (35.48%) suffered from obesity. Regarding the relationship between IMT and GLS value in the global population the correlation coefficient was 0.5758, while in the "overweight" group and "obesity" group the R value was equal to 0.6243 and 0.735, respectively. Our main results are illustrated in the following figures.

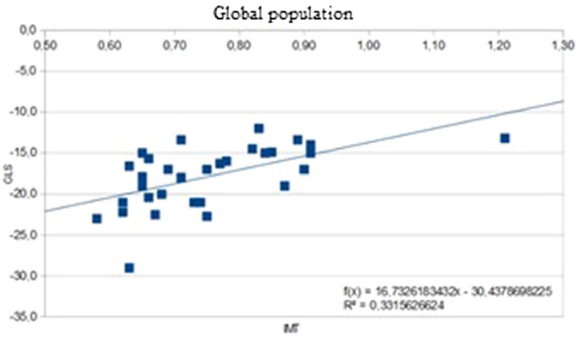

**Fig. 1** Linear regression of GLS and IMT in the global population.

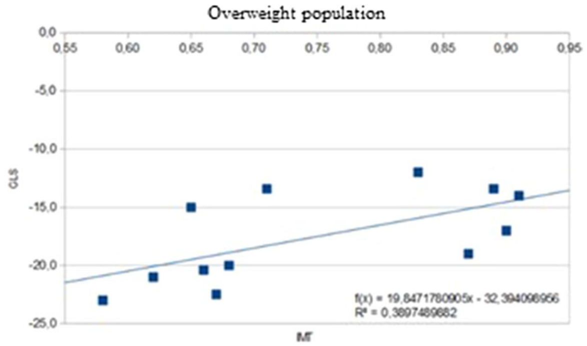

**Fig. 2** Linear regression of GLS and IMT in the overweight population.

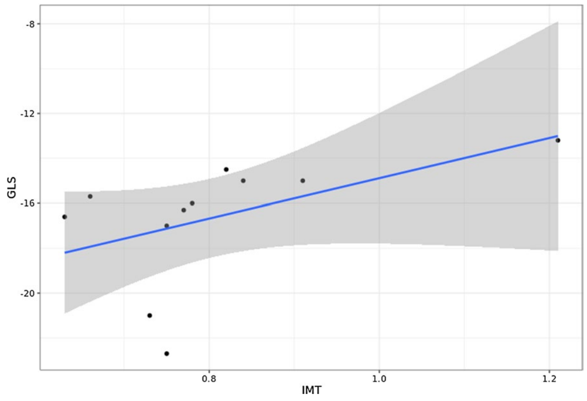

**Fig. 3** Spearman correlation of GLS and IMT in the obese population p = 0.00994.

**Conclusions:** Overall, our study revealed that the absolute value of GLS decreases while the measured IMT appears to increase with BMI values.

### P.26

### Back to the future. Cuffless blood pressure estimation in the 1990's

**Mr Kyrollos Louka**[1], Mr James Cox[1], Dr Isabella Tan[1], Dr Alberto Avolio[1], Mr Michael O'Rourke[2], Dr Mark Butlin[1]

[1]Macquarie University, Macquarie Park, Australia, [2]University of New South Wales, Sydney, Australia

**Background:** The new frontier of cuffless blood pressure (BP) may enable widespread, high-frequency BP estimation in the community. The technique, however, is not new. This study investigates the first commercial cuffless BP device, the Casio BP-100 digital watch (Model No. 900), released in 1993. Finger pulse arrival time is measured using finger photoplethysmography and an electrocardiogram. Using a two-point individualised calibration method, systolic BP (SBP) and diastolic BP (DBP) is estimated.

**Methods:** Twenty participants (11 female) had seated BP measured using the Casio BP-100 and a brachial automatic oscillometric BP device at rest and during a 5-min isometric hand-grip exercise.

Results: There was no significant difference between the reference device and the BP-100, but a large bidirectional scatter of BP estimation by the Casio BP-100 (average differences 2 ± 20 and -1 ± 19 mmHg during baseline, and -1 ± 21 and 7 ± 23 mmHg during exercise for SBP and DBP respectively). There was poor correlation for both SBP (R = 0.36, p = 0.13) and DBP (R = 0.044, p = 0.37).

**Conclusions:** Given the size, low processing power, and long battery life, it is impressive that the Casio BP-100 provided some directional information on BP. It is also one of the few commercial devices to this day using two-point individualisation calibration. Likely in part due to limitations in the algorithms for finding fiducial waveform points, BP estimation was poor. As we look to the future of cuffless BP, we should emulate the positive aspects of the Casio BP-100 whilst looking at ways to improve accuracy.

### P.27

### Comparison of Quantitative Reflection Indices of Forward–Backward Pulse Wave Decomposition Techniques: A Virtual Subject Study

**Mr. Rahul Manoj**[1], Mr. Kiran V Raj[1], Dr. P M Nabeel[2], Dr. Jayaraj Joseph[1]
[1]Department of Electrical Engineering, Indian Institute Of Technology Madras, Chennai, India, [2]Healthcare Technology Innovation Centre-IIT Madras, Chennai, India

**Background:** Studies of the forward–backward propagating pulses in arterial bed provide novel insights into vascular dynamics and clinical conditions. The conventional pulse wave separation techniques—flow-based Wave Separation Analysis (WSAMF) and Wave Intensity Analysis (WIA)—require measured pressure-flow waveforms (1). As alternatives, simplified methods that require pressure wave alone (modelled flow wave (2) or approximated to a triangular wave (WSATF) (3) have gained acceptance. This work compares performance of WIA, WSAMF, and WSATF using established wave reflection indices.

**Methods:** Methods' performance was evaluated on virtual subjects' data (4) (N = 500, age: 25–75 years). The pressure and flow waveform were extracted for the left carotid artery. Reflection Magnitude (RM), Reflection Index (RI), Pulse Pressure Backward (ΔPb) and Pulse Pressure Forward (ΔPf) were obtained and compared for said three methods.

**Results:** Samples of forward–backward pressure waves obtained from all the three methods are illustrated in Fig. 1. The comparative analysis is presented in Table I. Largest deviation in RM and RI was observed between WSATF and WSAMF (14.7% and 8.71%, respectively), and the minimum deviation was between WSAMF and WIA (6.66% and 3.71%, respectively). Deviation for ΔPb and ΔPf among methods ranged between 1.2% – 8.34%, with highest deviations against WSATF.

TABLE I: Comparison of reflection parameters for $WSA_{MF}$, $WSA_{TF}$, WIA

| Reflection Parameter | Method A - Method B | Bland-Altman Analysis | | Regression Annalysis | |
|---|---|---|---|---|---|
| | | Bias | 98% CI | R-Value | P-Value |
| RM | $WSA_{MF}$ & WIA | -0.02 | (-0.15 ,0.11) | 0.65 | <0.001 |
| | $WSA_{MF}$ & $WSA_{TF}$ | -0.089 | (-0.19, 0.02) | 0.72 | <0.001 |
| | WIA & $WSA_{TF}$ | 0.068 | (-0.03, 0.17) | 0.6 | <0.001 |
| | $WSA_{MF}$ & WIA | 1.56 | (-0.15, 0.11) | 0.64 | <0.001 |

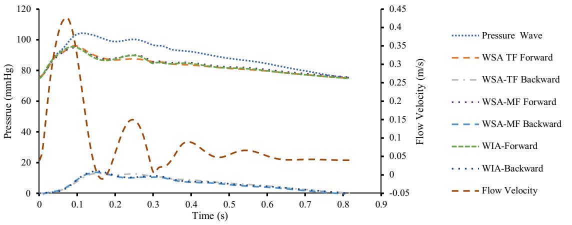

**Fig. 1** Comparison of wave separation in all the methods for a sample subject from database, along with pressure and flow velocity waveform extracted from left carotid artery.

### P.28

### Evaluation of Arterial Pulse Reflection Parameters using Multi-Gaussian Decomposition Model: Association with Stiffness Markers

Mr. Rahul Manoj[1], Mr. V Kiran Raj[1], Dr. P M Nabeel[2], Dr. Jayaraj Joseph[1]
[1]Department of Electrical Engineering, Indian Institute Of Technology Madras, Chennai, India, [2]Healthcare Technology Innovation Centre—IIT Madras, Chennai, India

**Background:** Recent methods to quantify arterial wave reflections perform wave separation analysis (WSA) based on single-site pressure and flow information to evaluate reliable metrics: Reflection Magnitude (RM) and Reflection Index (RI), contrary to conventional augmentation index (1). Addressing challenges associated with such methods, we have developed a new WSA technique using Multi-Gaussian Decomposition (MGD).

**Methods:** The MGD model decomposes the diameter-scaled pressure waveform into multiple Gaussians for WSA without requiring flow information. The method's functionality was investigated on 100 participants (35 ± 10 years, 50 hypertensives) where diameter measured using ARTSENS (2) were used to evaluate RM and RI. RM and RI are validated by their associations with stiffness markers and screening ability.

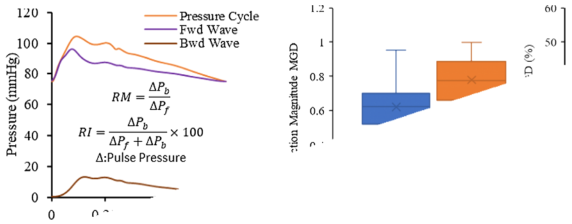

**Fig. 1**(a). Separated waves using MGD, (b) Box and Whisker plots for RM and RI comparing normotensives versus hypertensives.

**Results:** Adequately high-quality diameter waveforms were captured. The group averages of RM (= 0.69 ± 0.16) and RI (= 40.73 ± 6.1) % were comparable with earlier reported WSA studies (3–4). They exhibited significant correlation ($r > 0.5$, $p < 0.0001$) with the stiffness markers: β, elastic modulus, compliance, pulse wave velocity and AIx. Both RM and RI were significantly ($p < 0.05$) higher for hypertensives than normotensives, by 25.20% and 15.4%, respectively.

**Conclusion:** The study demonstrated the method's functionality in estimating reliable RM and RI that evidently associated with other clinically popular stiffness markers and discriminated between hypertensives and normotensives. Given the advantage that the method requires strictly one pulse waveform alone, its potential clinical and research applications are further being explored.

### P.29 Vascular function is unaltered after aerobic acute exercise in physically active young and older male adults

**Mr. João Luís Marôco**[1,2], Mr Marco Pinto[3], Dr. Helena Santa-Clara[4], Dr. Bo Fernhall[5], Dr. Xavier Melo[1,2]
[1]Faculdade de Motricidade Humana-Universidade de Lisboa, Oeiras,, Portugal, [2]Ginásio Clube Português, Research & Development Department, GCP Lab, Lisboa, Portugal, [3]Faculdade de Medicina da Universidade de Lisboa, Lisboa, Portugal, [4]Centro Interdisciplinar de Estudo da Performance Humana, Faculdade de Motricidade Humana – Universidade de Lisboa, Oeiras, Portugal, [5]College of Applied Health Sciences—University of Illinois at Chicago, Chicago, USA

**Purpose:** Vascular acute responses to successive bouts of exercise may cumulatively induce exercise-related adaptations in an intensity-dependent manner. However, these responses are inconsistent across age groups, and whether there are age or physical activity associated response patterns on brachial artery flow-mediated dilation (FMD) and arterial stiffness indices to high-intensity interval exercise (HIIE) and moderate-intensity continuous exercise (MICE) remains unknown. We compared the response pattern of FMD and arterial stiffness indices,

10 and 60 min following an acute bout of HIIE and MICE in physically active young and older adults.

**Methods:** Twenty four young (20–40 years; n = 12) and older (57–76 years; n = 12) healthy and active male adults performed an isocaloric acute bout of HIIE and MICE, or a non-exercise condition, in a randomized order. Pre-and-post condition changes in FMD, pulse wave velocity (PWV), and augmentation index (Aix) were analyzed with linear mixed models.

**Results:** Relative FMD was similar between young and older adults but time-to-peak was higher in older adults (d = 14; 95% CI: 4 to 23 s; $p < 0.01$, h2 = 0.28). Carotid-femoral PWV (cfPWV) (d = 2.16; 95% CI: 1.13 to 3.19 m/s; $p < 0.001$, h2 = 0.46), and Aix (d = 27.90; 95% CI: 21.27 to 34.50%; $p < 0.001$, h2 = 0.78) were higher in older compared to young adults. FMD (p = 0.84) and cfPWV (p = 0.85) remained unchanged following HIIE and MICE in both groups (Figure).

**Conclusions:** We found no evidence of age-associated response patterns on FMD and cfPWV to a single bout of HIIE or MICE in physically active young and older adults.

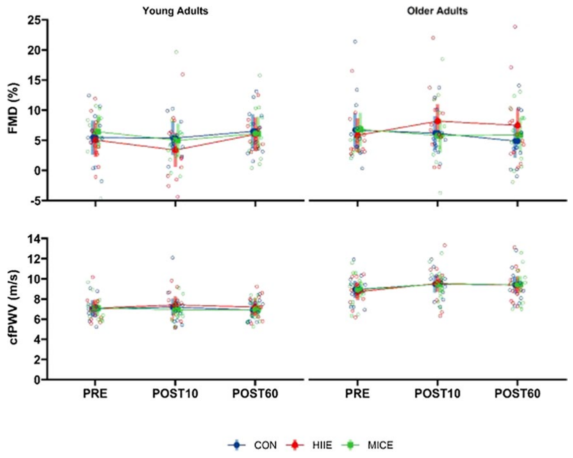

**Figure** Flow-mediated dilation (FMD) and carotid-femoral pulse wave velocity (cfPWV) post-exercise response in young and older adults. Vertical bars correspond to the 95% CI. Open circles correspond to individual responses. There were no significant main effects for FMD (time, condition, group) or interaction effects between factors. A significant group effect for cfPWV was observed ($p < 0.001$).

### P.30

### Portable ultrasound-based system for the assessment of carotid characteristics: a pilot study

MSc Martina Francesconi[1], **MSc, PhD Maria Raffaella Martina**[2], MSc Silvia Armenia[1], MSc Andrea Buzzelli[2], MD, PhD Gregorio Di Franco[3], MSc Vincenzo Gemignani[2], MSc, PhD Elisabetta Bianchini[2], MD, PhD Rosa Maria Bruno[4]

[1]Department of Clinical and Experimental Medicine, University of Pisa, Pisa, Italy, [2]Institute of Clinical Physiology (IFC), National Research Council (CNR), Pisa, Italy, [3]General Surgery Unit, Department of Translational Research and New Technologies in Medicine and Surgery, University of Pisa, Pisa, Italy, [4]INSERM U970 Paris Cardiovascular Research Centre–PARCC, Université de Paris, and AP-HP, Pharmacology Unit, Hôpital Européen Georges Pompidou, Paris, France

**Abstract**

**Purpose/background/objectives:** Effective strategies to prevent or minimize the burden of cardiovascular events [1] are based on early diagnosis and monitoring of cardiovascular risk. Early detection of vascular alterations in apparently healthy individuals can be implemented in large arteries, such as the carotid [2] and usable systems could improve the translation of this approach into clinical practice. Technical validation and usability of an innovative, easy-to-use, and portable system to assess carotid function and structure by ultrasound was investigated [3].

**Methods:** The new integrated system is composed of hardware (the Interson SP-L01 embedded ultrasound probe) and software measuring the instantaneous diameter of the carotid artery in real-time from B-mode ultrasound image sequences (Carotid Studio, by Quipu Srl).

12 healthy volunteers were recruited for the pilot technical validation: intra-operator reproducibility of two acquisitions by an expert operator and agreement with state-of-the-art technique (Mylab25 Esaote and Carotid Studio 4.3) were evaluated. Questionnaires were administered to operators to assess the usability of the new portable integrated system.

**Results:** Agreement with state-of-the-art technique was satisfactory, with no significant bias for the 12 subjects recruited (5 men, 44.5 ± 13.6 years). Coefficient of variation was 3.2% (2.5% SD) for Intima-Media Thickness, 0.9% (0.7% SD) for diameter, and 2.5% (2.2% SD) for distension.

An average score greater than 4 on a Likert five-point scale positively reported the usability of our system.

**Conclusions:** The innovative prototype for easier assessment of ultrasound carotid parameters of vascular ageing was successfully designed and developed. Usability in this pilot study was also satisfactory.

### P.31

### Identification of vascular damage in systemic sclerosis: results from a single centre cross-sectional study

**Dr. Carolina Mazeda**[1], Dr. Susana Silva[1], Dr. Renata Aguiar[1], Dr. Anabela Barcelos[1], Dr. José Mesquita Bastos[1]

[1]Centro Hospitalar Baixo Vouga Aveiro, Portugal

**Introduction:** Systemic Sclerosis (SSc) is a rare connective tissue disease characterized by fibrosis, imune and vascular dysfunction. Classical manifestations include changes in microcirculation, but macrovascular involvement hasn't fully understood. Arterial stiffness, measured using pulse wave velocity (PWV), is dynamic property determined by arterial wall structure, endothelial and vascular smooth muscle function, and arterial pressure. This measure is an independent predictor of cardiovascular events. The aim of study is comparison of arterial stiffness in SSc patients and healthy controls.

**Methods:** Patients with SSc fulfilled ACR/EULAR 2013 classification criteria. Socio-demographic and clinical data were collected. Arterial stiffness studies was performed (PWV and augmentation index (AI)). In descriptive analysis, ANOVA and Kruskal–Wallis test were used to compare continuous variables and Fisher's exact test for categorical variables. p-value ≤ 0.05 was statistically significant.

**Results:** Twenty-two patients were included (17 female and 5 men; mean age 58.95 ± 8.75) and 11 controls (7 female and 6 men; mean age 50.01 ± 10.09). PWV were significantly increased in SSc patients compared with controls (PWV: 8.08 ± 1.66 vs 6.85 ± 1.84), with significant differences in two groups (p = 0.01). Values of AI as also higher (8.57 ± 7.27 vs 6.26 ± 5.94), however no statistically significant differences was found (p = 0.35).

**Conclusion:** Although we were able to demonstrate significant difference in PWV between two groups, but it was insufficient to detect difference in AI. This suggests that patients with SSc may have an increased prevalence of subclinical atherosclerosis, however more studies with larger sample size are warranted and may be beneficial to assess its evolution over time in order to understand its impact on the clinical outcome.

### P.32

### Correlation between arterial stiffness and nailfold capillary microscopic abnormalities in systemic sclerosis: results from a single centre cross-sectional study

**Dr. Carolina Mazeda**[1], Dr. Susana Silva[1], Dr. Renata Aguiar[1], Dr. Anabela Barcelos[1], Dr. José Mesquita Bastos[1]
[1]Centro Hospitalar Baixo Vouga, Aveiro, Portugal

**Introduction:** Systemic Sclerosis (SSc) is a rare connective tissue disease with several systemic manifestations characterized by immune dysfunction, vascular abnormalities and fibrosis. Microvascular damage represents the earliest morphological and is a prominent feature of SSc easily accessible by nailfold video-capillaroscopy (NVC). While microangiopathy is well-documented, the macrovascular involvement has not been completely clarified. Arterial stiffness is an independent predictor of cardiovascular events and the carotid-femoral pulse wave velocity (PWV) are considered the gold standard for your measurement. The aim of this study is to assess changes in arterial stiffness and its relationship with NVC abnormalities in SSc patients.

**Methods:** All patients included fulfilled ACR/EULAR 2013 classification criteria for SSc. Each participant underwent arterial stiffness studies (PWV and augmentation index(AI)) and NVC (nonspecific, early, active or late). To assess differences of macrovascular indices between NVC patterns, Kruskal–Wallis test was used. The Fisher's exact test was used for categorical variables. p-value $\leq$ 0.05 was statistically significant.

**Results:** Twenty-two patients were included and clinical characteristics are listed in Table 1. Mean PWV was 8.08 ± 1.66 m/s and AI was 6.85 ± 1.84%. No significant differences between PWV and AI with NVC patterns ($p = 0.36$ and $p = 0.39$). Digital ulceration, disease subtype, pulmonary involvement and anti-topoisomerase-I antibodies were found statistically correlated with microangiopathic severity identified in NVC ($p < 0.05$), but no association was found between these findings and macrovascular indices.

**Conclusion:** Despite small sample size, wall stiffness parameters measurements don't seem to correlate to microangiopathic features. More studies with larger samples are needed, as well as understanding the evolution of stiffness parameters over time.

Table 1

| | |
|---|---|
| Age, years (mean±SD) | 58.95±8,75 |
| Sex (M/F), n | 5/17 |
| Duration disease (mean±SD) | 51.59±44.59 |
| Diffuse cutaneous, n (%) | 7 (31.8) |
| Modified Rodnan skin score (mean±SD) | 10.82±10.55 |
| Digital ulcers, n (%) | 11 (50.0) |
| Anti-Scl70 antibodies, n (%) | 7 (31.8) |
| Anticentromere antibodies, n (%) | 11 (50.0) |
| Anti-RNA polymerase III antibodies, n (%) | 3 (13.6) |
| Pulmonary involvement, n (%) | 8 (36.4) |
| Gastrointestinal involvement, n (%) | 9 (40.9) |
| Articular involvement, n (%) | 2 (9.1) |
| Nailfold video-capillaroscopy pattern | |
| Nonspecific | 1 (4.5) |
| Early | 8 (36.4) |
| Active | 7 (31.8) |
| Late | 6 (27.3) |

### P.33 Suitability of a representative aortic flow waveform for pressure-only wave separation in children and adolescents

**A/Prof Jonathan Mynard**[1,2,3], Hilary A Harrington[1], Dr Remi Kowalski[1,2,4], Jonathan Glenning[1,2], Avinash Kondiboyina[1,2], A/Prof Joseph Smolich[1,2], Prof Michael Cheung[1,2,4]
[1]Heart Research, Murdoch Children's Research Institute, Parkville, Australia, [2]Department of Paediatrics, University of Melbourne, Parkville, Australia, [3]Department of Biomedical Engineering, University of Melbourne, Parkville, Australia,[4]Department of Cardiology, Royal Children's Hospital, Parkville, Australia

**Background:** Wave separation analysis (WSA) reveals the contribution of forward and backward waves to the arterial pulse. Although WSA requires both pressure and flow waveforms, it is generally the pressure waveform that varies most between individuals. Therefore, a synthesized (e.g. triangular) waveform is now commonly used in settings where the flow waveform cannot be obtained.[1] However, the most appropriate flow waveform for use in children and adolescent is unknown.

**Methods:** Twenty-three children and adolescents (15 years old, 48% male, height 168 15 cm, weight 61 20 kg) attending the Royal Children's Hospital (Melbourne) for a cardiac MRI were recruited. Only patients with a normal left ventricle and aorta were included. Images from phase contrast MRI of the ascending aorta were segmented to obtain flow and cross-sectional area waveforms (the latter as a surrogate of pressure). Wave separation was performed with a) patient-specific flow, b) triangular flow waveforms with peak at 25% (Tri25) or 30% (Tri30) of ejection time, and c) a representative waveform obtained by averaging individual waveforms after amplitude and time normalisation and forcing late-diastolic flow to zero with a weighting function (Fig. 1).

**Results:** Normalised flow waveforms were highly consistent (Fig. 1). Compared with reflection magnitude (ratio of backward/forward wave amplitude) using patient-specific flows (0.58 0.17), Tri25 and Tri30 differed by –13.2 7.5% ($P < 0.001$) and –6.1 9.8% ($P = 0.003$), but the representative waveform was no different (0.6 5.3%, $P = 0.7$).

**Conclusions:** A representative population flow waveform is suitable for wave separation in children and adolescents, and provides superior accuracy compared with the triangulation approach.

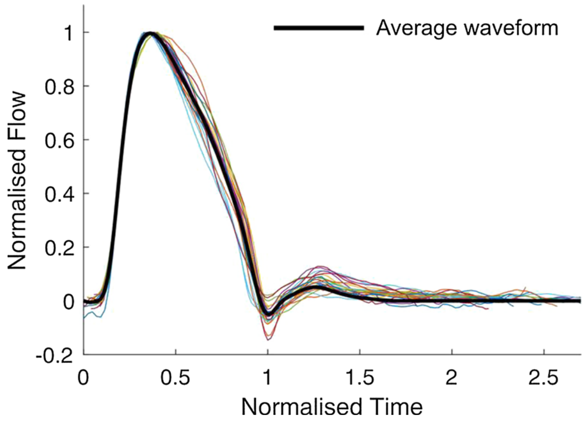

### P.34 Cross-sectional comparison of office and ambulatory pulse wave velocity by two methods, and their changes after lifestyle or medical interventions in hypertension

**MD, Phd János Nemcsik**[1], MD Dóra Batta[1], MD Beáta Zita Kőrösi[1], Helga Gyöngyösi[1], Zsófia Nemcsik-Bencze[1], MD, PhD Andrea László[2], MD, PhD Orsolya Cseprekál[1], MD, PhD András Tislér[1]
[1]Semmelweis University, Budapest, Hungary, [2]Jula/Schindler praxis, Nurenberg, Germany

**Objective:** Pulse wave velocity (PWV), the most accepted biomarker of arterial stiffening can be measured by different methods and in the past decade its 24-h monitoring has also become available. The aim of our study was to compare office and ambulatory PWVs and in a proportion of patients to compare the changes of PWVs after the initiation of lifestyle modifications or antihypertensive medication.

**Methods:** Office carotid-femoral PWV was measured with the tonometric PulsePen device (PP PWV), office and 24-h ambulatory oscillometric PWVs were evaluated with Mobil-O-Graph (MOB office PWV and MOB 24 h PWV, respectively). In new hypertensive patients the measurements were repeated 3 months after the initiation of antihypertensive medication. In white-coat hypertensive patients after lifestyle modifications the measurements were repeated at 12 months.

**Results:** 105 patients were involved with 22 new hypertensive (HT) and 22 white-coat hypertensive (WhHT) subjects. PP PWV (8.7 (7.3–9.9) m/s) differed from MOB office PWV (7.3 (6.5–8.8) m/s) and MOB 24 h PWV (7.4 (6.4–8.8) m/s) as well ($p<0.05$). PP PWV significantly decreased both in HT (by 0.9 (0.4–1.5) m/s, $p<0.05$) and WhHT patients (by 0.3 (− 0.1–1) m/s, $p<0.05$). MOB office PWV did not change significantly neither in HT, nor in WhHT. MOB 24 h PWV decreased only in HT patients (by 0.2 (0–0.6) m/s), which was less pronounced compared with PP PWV ($p<0.05$).

**Conclusions:** The significant differences observed both in the cross-sectional and in the prospective parts of our study suggests that the two methods are not interchangeable.

### P.35 Attenuation of the carotid-aortic stiffness gradient is associated with reduced microvascular perfusion in women with a history of preeclampsia

**Virginia Nuckols**[1], Amy Stroud[1], Debra Brandt[2], Lyndsey DuBose[1], Mark Santillan[2], Gary Pierce[1,3]
[1]Department of Health and Human Physiology, Iowa City, United States, [2]Department of Obstetrics and Gynecology, Iowa City, United States, [3]Department of Internal Medicine, Iowa City, United States

**Background:** Women with a history of preeclampsia (hxPE), a hypertensive pregnancy disorder, exhibit greater aortic stiffness postpartum compared with healthy pregnancy (HP). Greater relative carotid artery (CCA) compared with the aorta maintains a stiffness gradient that attenuates propagation of deleterious pulsatile pressure into the microcirculation.1 However, the relation between large artery stiffness, pulsatile hemodynamics and microvascular function among women with hxPE is unknown.

**Methods:** Women with hxPE ($N=33$) and HP controls ($N=46$) were assessed $18\pm6$ months postpartum. Aortic stiffness was measured as carotid-femoral pulse wave velocity (cfPWV) by applanation tonometry. CCA stiffness was assessed as characteristic impedance (Zc), the CCA pressure/flow ratio in early systole without the influence of wave reflection, using Doppler ultrasound.1 A single-point carotid-PWV was derived from stiffness parameters (Bramwell-Hill eq)2 for direct comparison to aortic (cfPWV). Sublingual microvessel perfusion (red blood cell filling) was assessed by sidestream dark- field imaging.

**Results:** Women with hxPE had greater CCA-Zc ($3562\pm202$ vs $2850\pm142$ DSC) compared with controls but cfPWV did not differ independent of mean arterial pressure ($6.1\pm0.2$ vs $5.5\pm0.1$ m/s, $P=0.55$). In HP, carotid-PWV exceeded cfPWV ($=0.4$ m/s, $P=0.005$) whereas in women with hxPE, carotid-PWV did not differ from cfPWV ($=-0.1$ m/s, $P=0.60$). CCA-Zc was associated with augmented carotid pressure pulsatility index ($r=0.36$, $P=0.005$) and reduced microvascular perfusion ($r=-0.26$, $P=0.03$).

**Conclusions:** HxPE is associated with greater large artery stiffness and attenuation of the CCA/aortic stiffness gradient. This may contribute in part to distal transmission of pulsatile pressure, reduced microvascular perfusion, and target-organ damage in women with hxPE.

### P.36 Numerical assessment of carotid-femoral pulse wave velocity in end-stage renal disease setting

**Dr Hasan OBEID**[1,2], Mrs Vasiliki BIKIA[4], Mrs Catherine FORTIER[2,3], Mrs Mathilde PARE[1,2], Mrs Karine DUVAL[1,2], Pr. Nikos STERGIOPULOS[4], Dr. Mohsen AGHARAZII[1,2]
[1]University Laval, Quebec, Canada, [2]CHU de Québec Research Center-L'Hôtel-Dieu de Québec Hospital, Quebec, Canada,[3]INSERM, UMR-970, Paris Cardiovascular research Center, PARIS 15, France, [4]Laboratory of Hemodynamics and Cardiovascular Technology, Swiss Federal Institute of Technology, Lausanne, Switzerland

**Background:** Through deregulation of various biological mechanisms, the uremic milieu plays a significant role in the cumulative vascular damage that results in aortic stiffness. Kidneys are high flow and low resistant organs, and their "absence" could significantly alter cardiovascular physiology in a way that could affect aortic stiffness based purely on a biomechanical analysis of the cardiovascular system.

**Methods:** We used a detailed 1D arterial network model (143 arterial segments) coupled with heart model. The cfPWV was determined by measuring the foot-to-foot pulse transit time (PTT) between the pressure signals at the carotid and the femoral arteries (cfPTT). We calculated cfPWV in four different settings. Setting 1: the right and left renal arteries were present in the 1D model (healthy subject), setting 2: the left renal arteries were removed from the model (kidney -donor subject), setting 3: both right and left renal arteries were removed from the model (end stage kidney failure), setting 4: both right and left renal arteries were removed, right renal arteries were attached on the external iliac artery (transplanted subject).

**Results:** In this numerical model, output cfPTT's were 110, 102, 99 and 101 ms respectively for setting 1, 2, 3 and 4. The cfPWV's were 4.82, 5.19, 5.35 and 5.25 m/s respectively for setting 1, 2, 3 and 4. This shows that numerical cfPWV increases in case of kidney disease and decreases for transplanted subjects.

**Conclusions:** The numerical assessment of cfPWV in ESRD setting is feasible using a 1D model of the arterial network. Further analyses are needed to mimic more realistic setting for the ESRD patients.

### P.37 The systolic rise time measured wih ppg to screening peripheral artery disease: application to the pOpmètre®

**Mrs Anissa Benbia**[1], Dr Magid Hallab[2], Mrs Kornelia Eveilleau[2], Dr Hasan OBEID[2], Dr Imad Abi-Nasr[2], Dr. Majid Tayyarah[3], Pr. Georges Leftheriotis[1]
[1]Service de Médecine Vasculaire, CHU de Nice-France, Nice, France, [2]Service de Cardiologie, Clinique Bizet, Paris-France, Paris, France, [3]Vascular Surgeon, Southern California Permanente Medical Group and Assistant Professor of Clinical Surgery, Loma Linda University School of Medicine, Southern California, USA, California, USA

**Introduction:** Screening for Peripheral Artery Disease (PAD) remains a challenge in the prevention and care of patients with arteriosclerosis. The Ankle Brachial Index (ABI) is currently the gold standard. However, ABI is time consuming and requires some expertise to perform which is a limiting factor for global screening. The measurement of the Systolic Rise Time (SRT) of the pulse wave of the lower limb may provide an easier alternative to detect PAD. In a retrospective pilot study, we analyzed the possibility of detecting PAD using the SRT of the toe waveform using the Photo-PlethysmoGraphic signal (PPG).

**Methods:** We measured 79 subjects (41 patients diagnosed with different stages of PAD and 38 healthy volunteers without known PAD). In each subject, at least one lower limb was assessed with classical ABI (minimum of 2 tibial arteries using a Doppler probe). All subjects underwent a PPG assessment on the finger and the toe simultaneously using pOpmètre® (Axelife—France). In-house software (JAVA) was used to calculate the SRT of all recorded signals.

**Results:** From the 154 lower limbs recorded, 8 were excluded for technical reasons: problematic cuff measurement of the ABI (in very severe PAD, medial calcification & amputation) or bad quality of the PPG signal with very low amplitude. Finally, 146 lower limbs were analyzed including 72 healthy and 74 subjects with PAD. The mean age of the population was 69 ± 12 years with 75% men, 28% diabetics, 47% hypertensive, 49% without clinical PAD defined as stage 0 in this study; 16% in stage 1; 32% in stage 2; 2% in stage 3 and 1% in stage 4 according to the Leriche classification. The SRT cut-off value of 160 ms identifies PAD according to Leriche starting at stage 1 with a sensitivity of 78% vs 73% for ABI < 0.9 and a specificity of 86% vs 87%; a positive predictive value (PPV) of 85% vs 86% and a negative predictive value (NPV) of 79% vs 75%. In addition, the ratio between toe-SRT and finger-SRT cut-off value of 1 identifies PAD with a sensitivity of 76% and a specificity of 74%.

**Conclusion:** SRT measured using a PPG pulse signal of the toe is promising as a facile non-invasive method to diagnose PAD (duration < 14 s) and determine PAD stages 1 to 4 with similar specificity and sensitivity as the reference method. These results need to be confirmed through a prospective study.

### P.38

### Effect of long term calorie restriction on transglutaminase-2 protein levels and microRNA expression of mice

**Elif Oztemiz**[1], Prof. Dr. Soner Dogan[2], Atakan Ayden[2], Assoc. Prof. Bilge Guvenc Tuna[1]

[1]Yeditepe University, Medicine Faculty, Biophysics Department, Istanbul/TURKEY, Istanbul, Turkey, [2] Yeditepe University, Medicine Faculty, Medicinal Biology Department, Istanbul, Turkey

**Background:** Transglutaminase-2 (TG2) is one of the important modulator of vascular health (1–3). This study aims to evaluate the protective effect of calorie restriction (CR) on vascular health through TG2 protein levels and microRNA (miRNA) expression targeting TG2 gene.

**Methods:** MMTV-TGF-α mice fed ad libitum (AL) divided into 3 groups; ad libitum(AL), chronic CR fed with %15 restriction of AL group, intermittent CR group fed AL for 3 weeks (ICR-ReFeed) which followed by %60 restriction for 1 week (ICR- Restricted). Mice euthanized at 10, 17/18 and 49/50 weeks and blood and aorta were collected respectively. Blood miRNA profile obtained by microarray (n = 3). Differentially expressed miRNAs were analyzed and miRNAtap package was used to obtain miRNAs targeting TG2 gene. TG2 levels of aorta were determined by western blot (n = 4).

**Results:** Compared to AL group at week 10, TG2 targeting miRNAs: miR-484, miR-700-5p and miR-423-5p were expressed statistically significant increase in ICR-R group at week 49/50. 17 weeks AL mice had increased TG2 protein levels compared to 10 weeks. However, the levels were decreased at 50 weeks group compared to both time points. CCR group had increased TG2 levels with ageing. ICR-R groups remained lower than CCR at both 17/18 and 49/50 week.

**Conclusion:** These results may provide a translational insight for understanding the roles of TG2 in vascular ageing and mechanism of epigenetic regulations by CR on providing better vascular health. In future studies, miRNAs that evaluated in this study will be validated in aorta samples to have more perception.

### P.39 Carotid stiffness and cerebral pulsatility index

**Ms Mathilde Paré**[1,3,4], Mr. Marc-Antoine Roy[3,4], Dre Catherine Fortier[5,6], Ms Audrey Drapeau[3,4], Ms Lawrence Labrecque[3,4], Ms Karine Duval[1], Dr. Patrice Brassard[3,4], Dr. Mohsen Agharazii[1,2]

[1]Division of Nephrology, Faculty of Medicine, Université Laval, Québec, QC, Canada, Québec, Canada, [2]CHU de Québec Research Center, L'Hôtel-Dieu de Québec Hospital, Quebec, Canada, [3]Research Center of the Institut Universitaire de Cardiologie et de Pneumologie de Québec, Québec, Canada, [4]Department of kinesiology, Faculty of Medicine, Université Laval, Québec, Canada, [5]INSERM, UMR-970, Paris Cardiovascular Research Center, 75,015, Paris, France, [6]AP-HP, Pharmacology Unit, Hôpital Européen Georges Pompidou, Université de Paris, Paris, France

**Purpose/background/objectives:** Aortic stiffness (AS) has been associated with accelerated cognitive decline, possibly due to increased cerebral blood flow (CBF) pulsatility and resulting microcirculatory damage. Hence, this study aimed to examine the association between aortic and carotid stiffness and their impact on the CBF pulsatility in a group of subjects composed of healthy controls and patients with chronic kidney disease (CKD), covering a wide range arterial stiffness measures.

**Methods:** In 19 participants aged 61 19 years (12 male, 13 CKD), we evaluated AS by carotid-femoral pulse wave velocity (CF-PWV). Common carotid diameter and distention (echotracking) and local pulse pressure (tonometry) were used to derive carotid pulse wave velocity (C-PWV) based on the Bramwell-Hill equation. Middle cerebral artery blood flow velocity (MCAv) was determined using transcranial Doppler ultrasound. MCAv pulsatility index (PI) was computed as (systolic MCAv—diastolic MCAv/mean MCAv. Spearman-rho correlation coefficients were examined to assess the extent of associations between stiffness parameters and MCAv PI.

**Results:** The CF-PWV and C-PWV were respectively 11.1 [9.4–12.8] and 7.8–2.9 m/s. Mean MCAv was 67.3–18.8 cm/s, systolic MCAv 96.1–25.3, diastolic MCAv 47.2–15.3 and MCAv PI was 0.75–0.21 cm/s. MCAv PI was significantly associated with C-PWV (r = 0.645, p = 0.004), however the association with CF-PWV did not reach significance (r = 0.447, p = 0.055). There was no association between MCAv PI and aortic-to-carotid PWV ratio (r = − 0.257, p = 0.303). Conclusions: This preliminary data shows a strong correlation between carotid stiffness and CBF pulsatility.

Table 1. Spearman-Rho correlation coefficients for carotid and aortic stiffness to cerebral flow velocities and pulsatility

| | MCAv mean (cm/s) | sMCAv (cm/s) | dMCAv (cm/s) | MCAv PI |
|---|---|---|---|---|
| C-PWV (m/s) | -0.329 (p=0.182) | -0.079 (p=0.754) | -0.430 (p=0.075) | 0.645 (p=0.004) |
| CF-PWV (m/s) | -0.480 (p=0.038) | -0.323 (p=0.177) | -0.584 (p=0.009) | 0.447 (p=0.055) |

Data is presented as correlation coefficient r (p-value)

Legend: MCAv: middle cerebral artery velocity; sMCAv: systolic MCAv; dMCAv: diastolic MCAv; MCAv PI: pulsatility index; C-PWV: carotid pulse wave velocity; CF-PWV: carotid-femoral pulse wave velocity

### P.40 Smooth muscle cells express stronger traction forces in aortic thoracic aneurysms

**Ms Claudie Petit**[1], Mr Ali-Akbar Karkhaneh Yousefi[1], Ms Olfa Ben Moussa[1], Mr Jean-Baptiste Michel[3], Mr Alain Guignandon[2], Mr Stéphane Avril[1]

[1]Mines Saint-Etienne, Université de Lyon, INSERM, U 1059 SAINBIOSE, F—42,023 Saint-Etienne, France, [2]Université Jean Monnet, Université de Lyon, INSERM, U 1059 SAINBIOSE, F—42,023 Saint-Etienne, France, [3]Laboratory for Translational Vascular Science, and Paris 7- Denis Diderot University, Xavier Bichat HospitalInserm UMR 1148, 75,018 Paris, FRANCE

**Introduction:** SMC have the ability to modulate their phenotype in response to pathological conditions, as occurs in ascending thoracic aortic aneurysms (ATAA) (1–3). In fact, it was previously shown that mis-sensing of mechanical stimuli play a major role in ATAA (2,4). Accordingly, there is a pressing need to quantify the mechanobiological effects of the changes operating at single cell level.

**Methods:** To address this need, we applied a previously developed Traction Force Microscopy (TFM) technique (5,6) on primary human aortic SMC, extracted from three healthy and three aneurysmal donors with matching age and gender. We measured the cell basal tone by measuring the traction forces applied by each SMC onto compliant hydrogels of different stiffness (4, 8, 12, 25 kPa), with embedded fluorescent microbeads (Matrigen, Softwell™ 24, collagen pre-coated, Softrack 0.2 μm Y/G).

**Results:** Although the range of measured force suggested some heterogeneity, we observed that: 1. the traction forces increased with the substrate stiffness; 2. For aneurysmal SMC, traction forces were significantly higher than for healthy ones. We also found the existence of larger traction forces in the aneurysmal SMC were related to the larger size of these cells (6).
**Conclusion and perspectives:** We conclude that there is a reduced expression of elongated and contractile SMCs in ATAA. This tends to promote stronger SMCs, which are less responsive to vasoactive agents. Future work aims at understanding further these alterations in aortic aneurysms.

### P.41 Roughness analysis of coronary artery stents and bypass grafts for diabetes mellitus patients

**Dr. Senol Piskin**[1]
[1]Department of Mechanical Engineering, Istinye University, Zeytinburnu, Turkey
**Purpose/background/objectives:** Atherosclerosis in coronary artery disease (CAD) or carotid artery disease is treated by implementing a stent through interventional cardiology or by deploying a bypass graft through open-heart surgery. Diabetes mellitus (DM) is one of the most common comorbid conditions in patients with coronary artery disease, which is important in determining the severity of the disease, treatment strategy, and the prognosis of patients [1].

Here, we aim to analyze and compare the DM patients' flow dynamics to healthy control using a computational model. We also aim to find out the effects of wall roughness on the WSS for the DM patient model.
**Methods:** Two different in silico models have been generated and simulated using computational fluid dynamics (CFD). For simplicity and isolate the geometrical contribution into the hemodynamics, idealized coronary artery models have been designed.
**Results:** WSS is higher at the proximity of the wall compared to the center of the artery for all cases: healthy control, DM patient and rough and smooth wall. Preliminary results of our study show that the natural wall roughness does not cause any SAWSS change for neither DM patients nor healthy subjects. But when the wall roughness is around $3{\cdot}10^{-4}$ m, the SAWSS decreases moderately.
**Discussion:** After deployment, stents can have and cause roughness [2]. Actually, these roughness values can be changed by designing novel stents or bypass grafts. Decreasing the WSS values around the stenosis region could help to improve the outcome of interventions or surgical operations.

### P.42 Effect of COVID-19 disease on vascular aging: a pilot study with before-and-after comparison in persons who have had COVID19.

Podrug M[1], **Koren P**[2], Šunjić B[1], Mudnic I[2], Boban M[2], Jerončić A[1]
[2]University of Split School of Medicine, [1]University of Split Department of Health Studies
**Background:** While respiratory symptoms dominate clinical manifestations of COVID-19 disease, the disease also affects the cardiovascular system at multiple levels. The aim of this study was to evaluate individual before-and-after COVID19-induced changes in vascular-aging biomarkers.

**Methods:** Previously, we collected data on baseline values of arterial stiffness and central and peripheral hemodynamic measures; in participants who were afterwards infected with SARS-CoV-2. Fifteen (7 men) agreed to an additional visit that was scheduled at median 2.4 months (range 1.8–3.1) after person acquired infection (determined by the positive PCR test). SphygmoCor-CvMS and Arteriograph were used to acquire the data in duplicate.
**Results:** The median age of participants was 35 years (range 24–61), and median time-span between the visits was 5 months (3–16). Regardless of limited sample size, we showed that after COVID-19 infection there was a significant mean increase of diastolic (5 mmHg, 95%CI 0.6–9) and mean arterial pressure (4 mmHg, 95%CI 0.1–8) recorded with Arteriograph; and median increase in aortic AIx (4%, 95%CI -2%_to_6%) recorded with SphygmoCor ($p<0.05$ for all). We also found a strong association between the time from infection, and variability of pulse wave velocity or end-systolic pressure (Kendall's_$\tau \geq 0.418$, $p \leq 0.035$). For several measures, the intensity of changes depended on age.
**Conclusions:** The results suggest widespread, complex changes in vascular biomarkers after the COVID-19 infection. While small sample size limits the certainty of our findings, overall the study prompts for implementation of powered, larger studies with adequate follow up to fully assess the effect of COVID-19 disease on vascular aging.

### P.43 Prevalence of fibromuscular dysplasia in radial arteries of cerebral aneurysms through ultra-high frequency ultrasound: a radiomic approach

**Biomedical engineer Federica poli**[1], Francesco Faita[2], Silvia Armenia[6], Michelangelo Mancuso[5], Paolo Perrini[5,6], Lorenzo Ghiadoni[5], Mirco Cosottini[4], Rosa Maria Bruno[1,3]
[1]Paris Cardiovascular Research Center (PARCC)-INSERM UMR-970, Paris, France, [2]Institute of Clinical Physiology, National Research Council, Pisa, Italy, [3]AP-HP, Hôpital Européen Georges Pompidou, Université de Paris, Paris, France, [4]Department of Translational Research and New Technologies in Medicine and Surgery, University of Pisa, Pisa, Italy,[5]University of Pisa, Pisa, Italy, [6]Azienda Ospedaliero Universitaria Pisana, Pisa, Italy
**Objectives:** Cerebral aneurisms are present in up to 12.9% patients with fibromuscular dysplasia (FMD) (1). We recently identified a pattern of vascular wall disarray and remodeling in radial arteries strongly and specifically associated with FMD. The aim of this study was to determine the prevalence of this pattern in radial arteries of patients with cerebral aneurysms.

**Methods:** 10 end-diastolic frames (5 left, 5 right) of the radial arteries of 30 patients with aneurisms and 24 healthy controls were obtained by VevoMD (70 MHz probe, FUJIFILM, VisualSonics, Toronto, Canada). 74 radiomic features and 4 engineered parameters were extracted: inner and outer layer thickness, and presence of adjunctive acoustic interfaces (triple signal). The same logistic regression (LR) model developed to discriminate FMD patients was used in this cohort. The possible association between this classification and the presence of ruptured aneurysm, multiple aneurysm and aneurysm size were investigated.
**Results:** Inner layer ($196 \pm 51$ vs $160 \pm 39$ mm, p=) and outer layer thickness ($115 \pm 45$ mm vs $101 \pm 41$ mm, p=) were significantly higher in aneurysms than in controls. Triple signal was also more frequent in aneurysm than in control images (p=). 17 patients with aneurysms (56.6%) were classified as FMD by the LR model. There was no significant association between classification as FMD and ruptured, multiple aneurysms, aneurysm size.
**Conclusions:** More than half patients with cerebral aneurysm showed a radial artery pattern associated with FMD; this finding suggest that a non-negligible proportion of patients with cerebral aneurysms may be part of the socalled FMD spectrum.

### P.44 Validation and feasibility of an automated system for the assessment of vascular structure and mechanical properties in the digital arteries through ultra-high frequency ultrasound

**Biomedical engineer Federica Poli**[1], Catherine Fortier[1], Hakim Khettab[3], Francesco Faita[2], Saverio Vitali[4], Giacomo Aringhieri[4], Lorenzo Ghiadoni[5], Stefano Taddei[5], Laurence Amar[3], Aurelien Lorthioir[3], Pierre Boutouyrie[1,3], Rosa Maria Bruno[1,3]
[1]Paris Cardiovascular Research Center (PARCC)-INSERM UMR-970 and Université de Paris, 75,015, Paris, France, Paris, France, [2]Institute of Clinical Physiology, National Research Council, Pisa, Italy, Pisa, Italy, [3]AP-HP, Hôpital Européen Georges Pompidou, Université de Paris, Paris,

France, Paris, France, [4]Diagnostic and Interventional Radiology, University Hospital of Pisa, Pisa, Italy, Pisa, Italy, [5]University of Pisa, Pisa, Italy, Pisa, Italy

**Objective:** The validation of a semi-automatic software to quantify vascular structure and mechanical properties of digital arteries acquired using ultra high frequency ultrasound (UHFUS).

**Methods:** UHFUS 5-s longitudinal scans of digital arteries of the hand of 15 patients with vascular diseases and 15 healthy controls were obtained by VevoMD (70 MHz probe, FUJIFILM, VisualSonics, Toronto, Canada) and analyzed using the semi-automatic Carotid Studio software (Quipu Srl, Pisa, Italy), using as reference technique a manual measurement in a Matlab interface (MathWorks, R2019b). Evaluation of agreement between the two techniques for measures of diameters (systolic,diastolic), distension and intima-media thickness (IMT) were made using Bland–Altman analyses; inter- and intra-operator reproducibility was carried out using coefficients of variation (CV).

**Results:** No trend or significant bias were observed between Carotid Studio and Matlab manual analysis for diastolic diameter, distension, and IMT. All limits of agreement were acceptable. Intra-observer CV of diastolic diameter and IMT were 4.1%, and 4.2% respectively. Inter-observer CV for diastolic diameter, and IMT were 7.3% and 5.4% respectively. Intra- and inter- observer CV for distension were higher (25.7% and 26.7% respectively).

**Conclusions:** Carotid Studio software is a valid and reproducible tool for the assessment of vascular structure and mechanical properties in UHFUS scans of digital arteries.

### P.45 Simulating the impact of parameter changes on the reservoir model

**Anna Pölz** [1,2], Stefan Orter[1,3], Siegfried Wassertheurer[1], Bernhard Hametner[1]

[1] AIT Austrian Institute of Technology GmbH, Center for Health & Bioresources, Giefinggasse 4, 1210 Vienna, Austria

[2] TU Wien, Institute of Engineering Hydrology and Water Resources Management, Karlsplatz 13, 1040 Vienna, Austria

[3] TU Wien, Institute of Electrodynamics, Microwave and Circuit Engineering, Gußhausstraße 24, 1040 Vienna, Austria

**Introduction:** The impact of alterations in arterial flow and pressure on the derived reservoir and excess pressure are not fully understood yet. The aim of this work was to generate pressure waveforms using a 3-element Windkessel model and apply the reservoir algorithm to analyze the changes in the output by varying the input parameters of flow and Windkessel.

**Methods:** An artificial flow curve (aF) was built, depending on the height (Fmax), time (tFmax) of its peak, and the ejection duration (ED). Pressure curves were afterwards simulated using aF and a 3-element Windkessel (peripheral resistance (R), compliance (C), aortic resistance (Z) and asymptotic pressure ($P_\infty$)). The reservoir algorithm then was applied to the simulated pressure waves (1). By parameter variation, a sensitivity analysis on reservoir pressure ($P_{res}$) and excess pressure ($P_{ex}$) was conducted. If the difference between every respective pair of output curves was below a certain threshold, the curves were counted as not being affected by the parameter variation.

**Results:** The sensitivity analysis of parameter variations (Fig. 1) shows that $P_{ex}$ is mostly sensitive towards $t_{Fmax}$, ED, and Z, while $P_{res}$ is sensitive towards R, C and $P_\infty$. When varying several parameters at once, cancelling effects of certain combinations were found.

**Discussion/conclusion:** The sensitivity analysis revealed that $P_{res}$ is mostly affected by Windkessel parameters, except for Z, while $P_{ex}$ is mostly affected by Z and flow parameters. However, $P_{res}$ and $P_{ex}$ also partly depend on the remaining parameters. The variation of parameters also revealed cancelling effects of certain combinations.

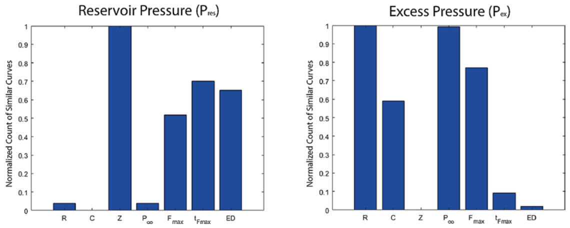

**Fig. 1** Results from sensitivity analysis: left $P_{res}$, right $P_{ex}$. The y-axis shows the normalized count of pairs of curves that remained similar (mean square error < 1 for $P_{res}$, mean square error < 0.25 for $P_{ex}$), despite changes in the input parameter that are shown on the x-axis.

### P.46 The Role of Blood Pooling during Prolonged Sitting on Cerebral Arterial Stiffness

**Alexander Pomeroy**[1], Katie Stanford[1], Dr. Lee Stoner[1]

[1]University of North Carolina At Chapel Hill, Chapel Hill, United States

**Purpose:** Vascular dementia (VaD) affects 15% of people 80 + years old. A primary risk factor for VaD is atherosclerosis of the cerebral arteries. Prolonged sitting has been associated with precursors of atherosclerosis, including arterial stiffening (AS) and acute reductions in cerebral blood flow (CBF), but the mechanism for cerebral hemodynamic changes is unclear. Venous blood pooling (VBP) in the calves due to gravity has been suggested as a potential cause for acute hemodynamic changes in the central vasculature, but the claim hasn't been evaluated in the cerebral vasculature.

**Methods:** 5 participants (n = 5, 23.6 [5.3] y, 40% F, 23.1 [3.2] kg/m$^2$) underwent two conditions, both with a two-hour sitting bout: CUFF, where bilateral occlusive cuffs were applied to the legs to induce venous pooling and NON-CUFF, which was a control condition. Cerebral AS was measured with Heart-MCA pulse wave velocity (hmPWV), and CBF was measured with mean volumetric blood flow through the common carotid artery. Results were analyzed using a random-effects mixed model and Cohen's d for effect size.

**Results:** hmPWV had a significant effect for time (ß = − 97.17, ES = 1.55), but not for condition (p = 0.636). The interaction between time and condition was significant (ß = 35.91, ES = 1.00) for CBF.

**Conclusions:** CBF decreased in the CUFF condition more than NON-CUFF over time, so VBP may be a driver of hemodynamic changes in the cerebral vasculature.

**Source of funding:** NONE.

### P.47 Fabricated data, manufacturer's tricks, and more: a couple of suggestions concerning guidelines for validation of pulse wave velocity measurement devices

**Prof Igor Posokhov**[1]

[1]Federal Medical Biophysical Center of Federal Medical Biological Agency, Moscow, Russian Federation

**Background:** Inconsistencies, such as increased PWV in healthy young people, became apparent in research using BPLab/Vasotens. This raised public health concerns. Raw data from validation study (as stated, according to 2010 ARTERY guidelines) [1] were examined. The raw data were published by me due to their problematic nature [2].

**Methods:** Dataset [2] contains:—files representing BPLab cuff BP oscillations;—several software versions (1999 – 2021) that calculate "vascular parameters" from them;—SphygmoCor measurements;—BPLab website screenshots;—fabricated data illustrations.

**Results:** There were only 2 age bands '< 30' and '> 60 years' here. There were signs of hidden editing: height of volunteers and path length distances went beyond normal human body ratios. For example

"proximal distance" box was filled with 90 in a volunteer 150 cm tall, or "jugulum-symphysis" with 32 in a volunteer 180 cm tall.
Screenshots show that one author is a service engineer of BPLab company.
The age dependence (key needed) was the same for software versions from 05.00.03 to 06.04.01. It was very different in the Italian study where version 06.02.?? was used [3]. It seems that 06.02 version was specifically designed to only publish the results.
**Conclusion:** I believe that guidelines should contain requirements for the mandatory publication of raw data as supplement. Researchers must have an adequate social responsibility, and confirming documents should be published as well. Each new version of software (e.g. 06.04.02, 06.04.03) must be validated in a new study, with new sets of raw data from new institutions.

### P.48 Development of carotid shear wave elastography for plaque characterization in transverse imaging planes.

Mrs. Judith Pruijssen[1], Dr. Stein Fekkes[1], Prof. Chris de Korte[1,2], **Dr. Hendrik Hansen**[1]
[1]Radboud university medical center, [2]University Twente

**Background:** Rupture of carotid artery plaques may lead to stroke. Plaques with a large lipid-rich core are more prone to rupture than predominantly fibrous plaques. Noninvasive detection of lipid-rich cores is challenging, but promising results have been reported using ultrasound shear wave elastography (SWE)(1,2). However, no available SWE method enables stiffness estimation, and thus, lipid-core detection, for the entire circumference. We introduce a SWE method designed for 360°-stiffness mapping and evaluate its performance in a carotid artery phantom.

**Methods:** A vessel-mimicking phantom with a stiff outer and soft inner layer (lipid-core) was created by freeze-thawing polyvinyl-alcohol solutions. An Aixplorer Ultimate system (Supersonic Imagine, France) with an SL18-5 transducer and research interface was used to perform SWE acquisitions in transverse cross-sections of the phantom. The 360° circumference was divided in segments. Custom-made software was developed to track the wave circumferentially in every segment, instead of along straight horizontal paths (current SWE approaches). Additionally, we investigated if segment-wise electronic ultrasound beam-steering improved tracking. Obtained 360° shear wave velocity (SWV) maps were compared to a reference map constructed from SWE data acquired by physical phantom rotation.
**Results:** Without steering, the new method provided SWV estimates with a median absolute error of ~ 0.5 m/s for ~ 60% of the circumference. With steering, this area increased to 80%. The soft layer could clearly be identified.
**Conclusions:** A novel SWE method was introduced that enabled accurate SWV estimation in ~ 80% of the cross-section of a vessel-mimicking phantom. Next the method will be tested in real carotid arteries.

### P.49 Comparison of artery wall motion-based vascular index with conventional carotid stiffness markers for detection of vascular ageing

**Mrs R Arathy**[1], Mr V Raj[2], Dr P M Nabeel[1], Dr Jayaraj Joseph[2]
[1]Healthcare Technology Innovation Centre-IIT Madras, Chennai, India,
[2]Dept. of Electrical Engineering, Indian Institute of Technology Madras, Chennai, India

**Background:** Large artery stiffness is a proxy for age-related vascular degradation and related events. Vascular ageing index (VAI), a metric of stiffness evaluated by pulse contour analysis of acceleration plethysmogram, was introduced to assess vascular ageing(1), but commonly used photoplethysmogram do not precisely capture artery wall dynamics. This study explores VAI assessment using precise carotid diameter recorded using an image-free ultrasound device (ARTSENS®) (2).

**Methods:** A cohort of 445 subjects (20–79 years) were recruited. Left common carotid diameter and blood pressure were recorded. VAI was estimated from carotid diameter adopting established methods (3). Age-trends of VAI were compared with conventional stiffness markers (2) for normotensives/hypertensives and males/females.
**Results:** Group average VAI (− 0.64 ± 0.48) measured were within the expected range (1),(4). Carotid stiffness markers ($r > 0.82$, $p < 0.01$) and VAI ($r = 0.6$, $p < 0.01$) were significantly correlated with subjects' age. Concomitant with stiffness markers, VAI manifested an increasing age trend (Fig. 1) with more negative values for younger populations. There was no significant difference for VAI ($p > 0.05$) between normotensive/hypertensive and male/female except for age ≥ 60 and 40–49 year's group ($p < 0.05$), respectively. Males showed higher VAI than females across age groups, whereas the trend was inverted for stiffness markers.

Fig 1. Carotid stiffness markers and VAI for normotensives/hypertensives and males/females.

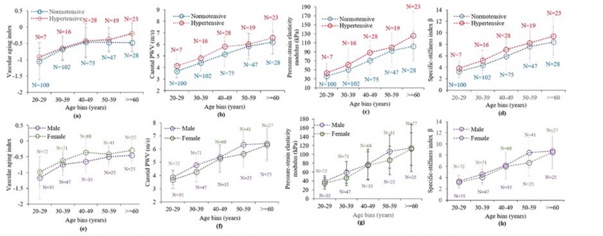

**Conclusion:** Reliably evaluated VAI from ARTSENS® carotid diameter demonstrated an age-related trend similar to stiffness markers. This suggests that the index obtained from diameter alone yields age-related modifications of large arteries. However, unlike the stiffness markers, the rate of increase in VAI was attenuated for the older population. Further studies are in progress to assess clinical utility and evaluate the predictive capability of VAI for monitoring vascular ageing.

### P.50 Effect of the pharmacological reduction of heart rate by ivabradine on arterial wall viscosity in young and middle-aged healthy subjects

**Dr Frédéric Roca**[1], Dr Michèle Iacob[1], Mrs Caroline Thill[1], Pr Jeremy Bellien[1], Dr Robinson Joannides[1]
[1]Rouen University Hospital, Rouen, France

**Purpose/background/objectives:** Changes in arterial wall viscosity (AWV), which dissipates the energy stored within the arterial wall, may contribute to the higher cardiovascular risk and arterial stiffening associated with high heart rate (HR) and to the controversial benefit of HR reduction during aging. We evaluated the effect of ivabradine, a selective HR- lowering agent, on carotid AWV considering changes in arterial mechanics and hemodynamics, and aging impact on this effect.

**Methods:** This randomized, placebo-controlled, double-blind, crossover study, performed in 19 healthy volunteers (8 young and 11 middle-aged), evaluates the effect of ivabradine (5 mg b.i.d, one week) on carotid AWV, mechanics and hemodynamics and cardiovascular coupling. AWV was evaluated by the area of the hysteresis loop of the pressure-cross sectional area relationship, representing the energy dissipated ($W_V$), and by the relative viscosity ($W_V/W_E$), with $W_E$ representing the elastic energy stored.
**Results:** Ivabradine increased stroke and end-diastolic volumes, augmentation pressure, augmentation index, Buckberg index and carotid distensibility compared to placebo. In parallel, $W_V$ and $W_E$ increased, and $W_V/W_E$ remained stable. In middle-aged, baseline arterial stiffness and cardiovascular coupling were less favorable, $W_E$ was similar but $W_V$ and thus $W_V/W_E$ were lower than in young subjects. Ivabradine induced an increase in $W_V/W_E$ in middle-aged subjects but not in young, due to a higher increase in $W_V$, despite similar increase in $W_E$.
**Conclusions:** Ivabradine-induced HR reduction increases arterial wall energy dissipation proportionally to the increase in elastic energy stored. Aging results in a larger than expected relative energy dissipation, the impact of which should be assessed.
Registration: URL: https://clinicaltrials.gov/ct2/show/NCT02584439, Identifier: 2015/077/HP.

### P.51 Evaluation of vascular and hemodynamic responses after a continuous exercise session of moderate intensity and high intensity intervals in individuals with normal blood pressure and pre-hypertension

**Miss Sara Rodrigues**[1], Miss Renata Verardino[1], Mr Marcel Costa[1], Miss Valéria Costa-Hong[1], Miss Maria Alves[1], Mr Luiz Bortolotto[1]
[1]InCor HC FM USP, São Paulo, Brazil

**Purpose:** Physical exercise (PE) prevents cardiovascular diseases 1. There is no consensus if different intensities of PE changes arterial stiffness—by pulse wave velocity (VOP) and augmentation index (AIx), a marker of cardiovascular risk 2, related to blood pressure (BP). Objective: In normotensive—normal BP (120–129/80–84 mmHg) and high normal BP (130–139/85–89 mmHg), compare: 1st) arterial stiffness after one session of moderate-intensity continuous PE (MICPE) and high-intensity interval PE (HIIPE). 2nd) BP after MICPE and HIIPE. **Methods:** PE intensity and equalized energy expenditure defined by cardiopulmonary exercise test. Individuals randomized to PE sessions, performed in cross-over fashion. PWV and AIx were measured at rest, immediately after and 24 h after HIIPE and MICPE, compared among all moments (baseline, immediately and 24 h after each session). Ambulatory BP monitoring-24 h (ABPM-24 h) was performed after rest and each session, using the first two hours for comparison. **Results:** Individuals (N = 29; 76%women; age = 48 ± 7y; BMI = 28.3 ± 4 kg/m2; SBP = 126 ± 9; DBP = 84 ± 4 mmHg) had lower ($p < 0.01$) AIx after MICPE (27.1 ± 2.0) and HIIPE (22.7 ± 2.2), than baseline (33.0 ± 1.8). AIx after MICPE (27.1 ± 2.0) was lower ($p < 0.05$) than MICPE24h (30.6 ± 2.3). AIx after HIIPE (22.7 ± 2.2) was lower ($p = 0.01$) than MICPE (27.1 ± 2.0), and than HIIPE24h (32.2 ± 1.9). SystolicBP (2 h) reduced after both sessions –MICPE (128 ± 2) and HIIPE (127 ± 2 mmHg), compared to baseline (131 ± 2 mmHg; $p = 0.02$). There was no difference in PWV among times, and between individuals with normal BP and high normal BP.

**Conclusion:** In normotensive, one PE session, regardless of intensity, reduces systolicBP during the first 2 h and AIx immediately after, returning to baseline values 24 h after PE session.

### P.52 Comparison of hemodynamic and vascular responses between a session of continuous moderate-intensity and high-intensity interval physical exercise in normotensive subjects

**Miss Sara Rodrigues**[1], Miss Renata Verardino[1], Mr Marcel Costa[1], Miss Valéria Costa-Hong[1], Miss Maria Alves[1], Mr Luiz Bortolotto[1]
[1]InCor HC FM USP, São Paulo, Brazil

**Purpose:** Physical exercise (PE) prevents cardiovascular diseases1. There is no consensus if different intensities of PE changes arterial stiffness, by pulse wave velocity (PWV), a marker of cardiovascular risk2, related to blood pressure (BP).

**Objective:** In normotensive (BP < 140/90 mmHg), to compare a) PWV responses to an moderate-intensity continuous aerobic physical exercise (MICPE) and high-intensity interval physical exercise (HIIPE) session; b) BP profile between MICPE and HIIPE session.
**Methods:** PE-intensity and equalized energy expenditure were defined according to the cardiopulmonary exercise test. Individuals were randomized to the sequence of PE sessions, performed in a cross-over approach. PWV measurements were analyzed at rest, immediately, and 24 h after a session of HIIPE and MICPE, results were compared among all moments (baseline, immediately after and 24 h after each session). Ambulatory BP monitoring (ABPM-24 h) was performed after baseline and each PE session, using the first two hours BP values for comparison analyses.
**Results:** Normotensive subjects (N = 31; 74% women; age = 48 ± 7 years; BMI = 28.4 ± 4 m/kg$^2$; SBP = 124 ± 10 and DBP = 83 ± 5 mmHg) had lower PWV ($p = 0.035$) 24 h after MICPE session (7.0 (6–10) m/s), compared to baseline (8.0 (6–9) m/s) and 24 h after HIIPE; (8.0 (5–10) m/s). PWV immediately after HIIPE was lower than baseline (8.0(5–9) vs. 8.0 (6–9) m/s; $p = 0.007$). SystolicBP (first 2 h) reduced ($p = 0.02$) after MICPE (129 ± 2 mmHg) and HIIPE (127 ± 2 mmHg), compared to baseline (131 ± 2 mmHg). The heart rate was similar after HIIPE (83 ± 2 bpm) and MICPE (83 ± 2 bpm) and higher ($p = 0.015$) than baseline (79 ± 2 bpm). Conclusion: In normotensive individuals, an MICPE session reduces PWV after 24 h and SystolicBP decreases for 2 h after an exercise session at both intensities.

### P.53 Comparison of vascular and hemodynamic responses between a continuous exercise session of moderate intensity and high intensity interval exercise in normotensive individuals.

Miss Sara Rodrigues[1], Miss Renata Verardino[1], Mr Marcel Costa[1], Miss Valéria Costa-Hong[1], Miss Maria Alves[1], Mr Luiz Bortolotto[1]
[1]InCor HC FM USP, Sao Paulo, Brazil

**Introduction:** Moderate-intensity continuous aerobic physical exercise (MICPE) is known to help prevent hypertension, due to its post-exercise hypotensive effect 1. High-intensity interval physical exercise (HIIPE) has shown positive results in preventing cardiovascular disease. Arterial stiffness, measured by pulse wave velocity (PWV) is a marker of cardiovascular risk 2, related to blood pressure (BP). There is no consensus on the possible changes in PWV after a physical exercise session.

**Objective:** In normotensive individuals (BP ≤ 140/90 mmHg), compare 1st: PWV responses to an MICPE and HIIPE session, 2nd: systolic BP (SBP) and diastolic BP (DBP) between an MICPE and HIIPE session.
**Methods:** Exercise intensity and calculation for equalizing energy expenditure were defined according to the cardiopulmonary exercise test. Individuals were randomized to the sequence of exercise sessions, performed in a cross-over fashion. PWV measurements were analyzed at rest, immediately after and 24 h after a session of HIIPE and MICPE in normotensive individuals, and results were compared between all moments (baseline, immediately after and 24 h after each session). Ambulatory BP monitoring 24-h (ABPM-24 h) was performed after the baseline session and after each exercise session. For comparison analyses, the first two hours measured in 24-h ABPM were used.
**Results:** Normotensive individuals (N = 31; 74% women; age = 48 ± 7 years; BMI = 28.4 ± 4 m/kg$^2$; SBP = 124 ± 10 and DBP = 83 ± 5 mmHg) had lower PWV ($p = 0.035$) 24 h after the MICPE session (7.0 (6–10) m/s), compared to baseline (8.0 (6–9) m/s) and 24 h after HIIPE; (8.0 (5–10) m/s). PWV immediately after HIIPE was lower than baseline (8.0 (5–9) vs. 8.0 (6–9) m/s; $p = 0.007$). There was a significant reduction ($p = 0.02$) in SBP (first 2 h) with both exercises—MICPE (129 ± 2 mmHg) and HIIPE (127 ± 2 mmHg), compared to baseline (131 ± 2 mmHg). The HR was similar after the HIIPE (83 ± 2 bpm) and MICPE (83 ± 2 bpm) exercise sessions and higher ($p = 0.015$) than the baseline (79 ± 2 bpm). Conclusion: In normotensive individuals, an MICPE session reduces PWV after 24 h and SBP decreases for 2 h after an exercise session at both intensities.

### P.54 The aortic-femoral arterial stiffness gradient demonstrates good between-day reliability

**Ms Jacklyn Rojas**[1], Mr. Keeron Stone[5], Dr. Simon Fryer[5], Dr. James Faulkner[2], Dr. Michelle Meyer[1], Dr. Kevin Heffernan[3], Gabriel Zieff[1], Craig Peterson[2], Dr Danielle Lambrick[4], Dr Lee Stoner[1]
[1]The University of North Carolina at Chapel Hill, Chapel Hill, United States, [2]University of Winchester, Winchester, United Kingdom, [3]Syracuse University, Syracuse, USA, [4]University of Southampton, Southampton, UK, [5]University of Gloucestershire, Gloucestershire, UK

**Background and aims:** The aortic-femoral arterial stiffness gradient (af-SG), defined as the ratio of femoral-ankle pulse-wave velocity (faPWV) to carotid-femoral pulse-wave velocity (cfPWV), is a promising marker of cardiovascular disease (CVD) risk. Yet to be of value in clinical and research settings, an arterial health assessment tool must be reliable (precise). This study sought to determine the between-day reliability of the af-SG.

**Methods:** Twenty-five, non-smoking, young healthy adults (40% female, age 22.6 ± 2.7 years, body mass index 23.9 ± 2.8 kg/m$^2$) were tested under standardized conditions on three different mornings in a fasted state, separated by a maximum of seven days. In a supine position, measures of cfPWV and faPWV were recorded in triplicate. The af-SG was calculated as faPWV divided by cfPWV. Intra-class correlation coefficient (ICC), standard error of measurement (SEM), and minimal detectable change (MDC) were calculated.

**Results:** The af-SG (ICC = 0.77, SEM = 0.08 m/s), cfPWV (ICC = 0.84, SEM = 0.29 m/s) and faPWV (ICC = 0.84, SEM = 0.38 m/s) measures all demonstrated good between-day reliability, according to accepted ICC criteria. The MDC (MDC%) between repeat measures within an individual was 0.22 (13.8%) for af-SG, 0.79 m/s (14.2%) for cfPWV, and 1.05 m/s (13.8) for faPWV.

**Conclusions:** These findings indicate that the af-SG demonstrates good reliability in young healthy adults. Further research is needed to identify if af-SG measurement variability is affected by age or disease.

**Keywords**: Central arterial stiffness, peripheral arterial stiffness, pulse wave velocity ratio, repeatability, reproducibility.

### P55. Evaluation of image-free wall tracking based measurement of low flow mediated arterial constriction in comparison to B mode imaging

Ms Sakshi Sen[1], Mr V Raj[2], Dr P M Nabeel[3], Dr Dinu S Chandran[1], Dr Jayaraj Joseph[2], Dr Kishore K Deepak[1]

[1]Department of Physiology, All India Institute of Medical Sciences, New Delhi, India, [2]Dept. of Electrical Engineering, Indian Institute of Technology Madras, Chennai, India, [3]Healthcare Technology Innovation Centre-Indian Institute of Technology Madras, Chennai, India

**Background:** Low flow mediated constriction (LFMC) has been reported as a promising non-invasive tool for assessing the endothelial functioning in resting state (1). LFMC is the constriction shown by a peripheral conduit artery in response to decreased blood flow through the lumen produced by distal circulatory arrest. LFMC is conventionally performed with B- mode ultrasound systems that involve recording of the image sequences as DICOM files or video-graphic files and processing them offline. We did a pilot evaluation to assess the correlation of imaging free wall tracking based measurements of LFMC with that of B mode imaging.

**Methods:** 29 healthy young adults (25.45 ± 2.58 years, 17 men] participated in the study. Brachial artery LFMC was measured using simultaneous and sequential protocols; imaging (M7, Mind Ray; Nanshan, Shenzhen, China) and wall tracking (ARTSENS®) based measurements were done in the same anatomical location with a gap of 30 min between the two measurements (sequential protocol) and imaging and wall tracking measurements done simultaneously at two contiguous locations (simultaneous protocol).

**Results:** LFMC responses showed strong positive correlation between ultrasonographic imaging and wall tracking based measurements when done simultaneously (r = 0.96, p < 0.001) and modest correlation when done sequentially (r = 0.59, p = 0.02) as shown in Fig. 1.

**Conclusion:** Wall tracking based measurements of brachial artery LFMC correlates strongly with imaging based measurements when recorded simultaneously. The study demonstrated that the wall tracking based ARTSENS® technology can be reliably employed for performing LFMC and assessing endothelial dysfunction.

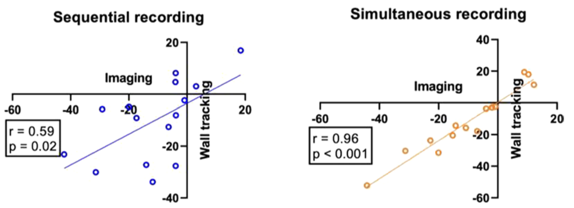

**Fig. 1** Correlation between %LFMC measured by B mode imaging and image free wall-tracking in sequential and simultaneous protocols.

### P.56 Preserved muscle extraction during maximal exercise in active breast cancer survivors.

**Sara Sherman**[1], Georgios Grigoriadis[1,2], Bo Fernhall[1], Tracy Baynard[1]

[1]Integrative Physiology Lab, University of Illinois at Chicago, Chicago, United States, [2]Department of Physical Therapy, Chicago, United States

**Introduction:** Mitochondrial capacity is pivotal to skeletal muscle function and is altered with breast cancer treatment. Physical activity may ameliorate the detrimental effects of such treatments on muscle function.

**Purpose:** To examine skeletal muscle tissue oxygenation in active breast cancer survivors (BCS) compared to inactive BCS during peak aerobic exercise.

**Methods:** Eleven active BCS (48 ± 9 years; 24.8 ± 2.6 kg/m$^2$) and 12 inactive BCS (51 ± 9 years; 26.1 ± 3.1 kg/m$^2$) performed a peak cycle ergometry test ($O_2$peak), wherein near infrared spectroscopy (NIRS) assessed muscle tissue oxygenation (tissue oxygenation index [TSI]; total [TH], oxygenated [$HbO_2$] and deoxygenated [HHb] hemoglobin) of the right vastus lateralis. Measurements were taken in the seated position on the cycle ergometer at rest and at $O_2$peak (max). Cardiac output was estimated using Doppler echocardiography and indexed to body surface area (Qi).

**Results:** Self-reported active BCS had a greater decrease in TSI from higher values at rest, despite similar peak values (p = 0.04; rest, max; active: 72 ± 3, 65 ± 5%; inactive: 67 ± 4, 63 ± 4%). Similarly, active BCS had higher TH and HbO2 overall (p < 0.05), whereas HHb was greater at max for both groups (p = 0.02; active: 45 ± 15, 58 ± 21 μM; inactive: 35 ± 13, 44 ± 16 μM). $O_2$peak was significantly higher for the active BCS (active: 32.7 ± 4.3 vs. 21.0 ± 3.1 mL/kg/min), with a greater increase in Qi (p = 0.02; active: 1.86 ± 0.4, 7.5 ± 2.0 L/min/m$^2$; inactive: 1.97 ± 0.5, 6.0 ± 1.1 L/min/m$^2$) for the active BCS during peak exercise.

**Conclusion:** Physical activity appears to attenuate impairment of skeletal muscle tissue oxygen extraction in active BCS, which may have implications for future research.

### P.57 Accuracy of cuffless blood pressure estimation using photoplethysmography and tonometry from pulse transit time alone

**Mrs Fatemeh Shirbani**[1], Dr Isabella Tan[1], Prof Alberto Avolio[1], Dr Mark Butlin[1]

[1]Macquarie University, Sydney, Australia

**Background:** Cuffless blood pressure (BP) estimation by pulse transit time (PTT) has advantages over standard cuff-based methods as it can be continuous and non-occlusive. However, the need for individual calibration between BP and PTT, and repeatability of calibrations are challenging.

**Objectives:** This pilot study investigates the repeatability and accuracy of BP estimation from PTT using contact-based photoplethysmography (PPG) and tonometry.

**Methods:** Six subjects performed one-minute seated rest and 3-min isometric handgrip exercise on two consecutive days. Continuous finger BP was recorded. A PPG sensor was placed adjacent to a tonometer on the temporal pulse and a second PPG sensor on the left index finger and tonometer on left radial pulse. Beat-to-beat PTT was calculated for tonometry and PPG as the delay between the foot of temporal and wrist/finger signals. DBP/PTT linear regression on day 1 was used as calibration for prediction of DBP from PTT on day 2, and the regression from day 2 applied retrospectively for day 1. DBP was predicted using every 1, 5, 10, 20 and 30 beats observations. Predicted DBP was compared to measured finger DBP.

**Results:** Isometric exercise successfully increased DBP (average increase 23 ± 11 mmHg). The minimum error of DBP estimation from PPG sensors was from 30-beats long observations (MAE ± ESD = 15 ± 0.5 mmHg) and from tonometers was from 20-beats long observations (MAE ± ESD = 13 ± 1 mmHg).

**Conclusion:** Cuffless BP estimated from PTT measured by PPG and tonometry provide a large error. In comparison, tonometry provides slightly better results as it requires less number of beats for estimation.

### P.58 Relationship between the parameters of aortic stiffness and nocturnal dipping status during antihypertensive therapy

**Dr. Anna Torunova**[1]
[1]Irkutsk State Medical Academy of Postgraduate Education-Branch Campus of the FSBEI FPE RMACPE MOH Russia, Irkutsk, Russian Federation

**The** aim: to study the dynamics of the relationship between aortic stiffness and nighttime BP decrease during the hypertension (HTN) treatment.

**Methods:** The 24-h BP monitoring was conducted in 189 patients (101 men and 88 women, aged 50.8 ± 8.3) with untreated HTN. Dipping status, central BP (cBP), augmentation index (AIx), carotid-femoral pulse wave velocity (PWV) were determined. Then the patients were assigned to standard antihypertensive therapy. The studies were repeated after 6 months of treatment. Spearman correlations between parameters of aortic stiffness and diurnal index (DI) were calculated twice—at baseline and at the end of follow-up period.

**Results:** During the treatment the average daily BP decreased from 143.6 ± 14.7/88.1 ± 11.1 to 123.9 ± 10.3/76.0 ± 7.2 mmHg, cBP—from 149.8 ± 19.4/98.3 ± 11.6 to 121.3 ± 14.0/83.2 ± 8.7 mmHg, AIx – from 25.9 ± 9.8 to 20.6 ± 12.2% and PWV from 9.2 ± 1.8 to 8.4 ± 1.5 m/s. Initially there were 117 (61.9%) dippers, 42 (22.2%) non-dippers, 22 (11.6%) over-dippers and 8 (4.3%) night-pickers. After 6 months of treatment, the proportion of dippers decreased from 61.9 to 48.1% while nondippers increased from 22.2 to 32.2% (p = 0.01). At baseline, systolic DI correlated with systolic cBP (r = − 0.22, p = 0.002), AIx (r = − 0.15, p = 0.046), PWV (r = − 0.14, p = 0.048). The diastolic DI correlated with diastolic cBP (r = − 0.23, p = 0.001) and AIx (r = − 0.22, p = 0.003). After 6 months of treatment there were no correlations between aortic stiffness and nocturnal dipping parameters.

**Conclusion:** In untreated HTN patients, the degree of nighttime BP decrease declines as aortic stiffness increases. Antihypertensive therapy leads to a loss of this relationship.

### P.59 The effect of renin-angiotensin system inhibitors on pulse wave velocity progression in essential hypertension patients: a 3.5-year follow-up study

**MD Myrthe van der Bruggen**[1], PhD Koen D. Reesink[1], PhD Alessandro Maloberti[2], MD, PhD Tammo Delhaas[1], PhD Casper G. Schalkwijk[3], MD, PhD Cristina Giannattasio[2], MD, PhD Rosa Maria Bruno[4], PhD Bart Spronck[1]
[1]Department of Biomedical Engineering, CARIM School For Cardiovascular Diseases, Maastricht University, Maastricht, The Netherlands, [2]School of Medicine and Surgery, Milano-Bicocca University, Milan and Cardiology 4, Niguarda Hospital, Milan, Italy, [3]Department of Internal Medicine, CARIM School For Cardiovascular Diseases, Maastricht University, Maastricht, The Netherlands, [4]French Institute of Health and Medical Research, Paris-Cardiovascular Research Center PARCC-INSERM, Paris, France

**Background:** Blood pressure (BP) control is an important treatment strategy in reducing arterial stiffness, as with lower BP the cyclical pressure-load on the arterial wall is reduced [1]. There is increasing evidence that anti-hypertensive drugs which antagonize the renin-angiotensin system (RAS) are superior in reducing arterial stiffness compared to other anti- hypertensive drugs [2]. In the current longitudinal follow-up study, we investigated if RAS-inhibitors reduced arterial stiffness more than other types of anti-hypertensive drugs.

**Methods:** Data was obtained from the Hypertensive Unit of two Italian hospitals (north Italy, $n = 447$, and Pisa $n = 101$) [3]. Carotid-femoral pulse wave velocity (cfPWV), brachial BP, and information on several confounders were obtained as part of clinical follow-up routine. Associations between ΔcfPWV and anti-hypertensive drugs class were assessed using multivariable linear regression analysis adjusting for age, sex, Δmean arterial pressure (MAP), Δheart rate, lifestyle factors, and use of other medication. Since cfPWV is intrinsically pressure dependent, we additionally performed our analysis replacing cfPWV with a pressure-corrected counterpart (carotid-femoral stiffness index $\beta_0$, [1]).

**Results:** Participants had a mean age of 54 ± 12 years, 47.1% were female, and mean follow-up time was 45 ± 8 months. A total of 431 participants used RAS-inhibitors at follow-up (32.5% RAS-inhibitor only, 67.5% combination therapy).
Neither ΔPWV nor $\Delta\beta_0$ was associated with the use of RAS-inhibitors in the regression models ($p = 0.33$, $p = 0.68$ respectively; **Table**).

**Conclusion:** RAS-inhibitors did not have a favorable effect on arterial stiffness progression compared to other types of antihypertensive drugs. Our next step is to evaluate cfPWV/$\beta_0$ changes in individuals starting RAS-inhibitors.

**Table. Associations between use of renin-angiotensin system inhibitors at follow-up visit and change in arterial stiffness**

| Model | ΔcfPWV β (95% CI) | $\Delta\beta_0$ β (95% CI) |
|---|---|---|
| 0 | 0,049(-0,037)0,135 | 0,061(-0,024)0,146 |
| 1 | 0,022(-0,066)0,111 | 0,019(-0,068)0,106 |
| 2 | 0,041(-0,044)0,126 | 0,015(-0,072)0,102 |
| 3 | 0,043(-0,043)0,129 | 0,018(-0,069)0,106 |

Model 0: crude associations. Model 1: model 0 + other types of antihypertensive drug classes. Model 2: model 1 + mean arterial pressure and mean heart rate. Model 3: model 2 + diabetes mellitus, use of statins, body mass index, high-density lipoprotein level, low-density lipoprotein level, total cholesterol level, triglyceride level, and blood glucose levels. cfPWV, carotid-femoral pulse wave velocity; $\beta_0$, carotid-femoral stiffness index $\beta_0$ (pressure-corrected counterpart of cfPWV).

### P.60 Central arterial pressure: validation of new cost-effective device against sphygmocor

**Dr. Valentina Vassilenko**[1,2,3], Andreia Serrano[1,2,3], Filipa Cardoso[1,2,3], Dr. Pedro Cunha[3,4,5]
[1]Nova School of Science and Technology, Nova University of Lisbon, Caparica, Portugal, [2]NMT, S.A., Caparica, Portugal,[3]Iberian Network on Arterial Structure, Central Hemodynamics and Neurocognition,, Portugal, [4]Internal Medicine Department, Center for the Research and Treatment of Arterial Hypertension and Cardiovascular Risk, Guimarães-Centro Hospitalar do Alto Ave/Minho University, Guimarães, Portugal, [5]Life and Health Science Research Institute (ICVS), School of Health Science, University of Minho, Braga, Portugal

**Central** aortic pressure (CAP) is an important marker to evaluate blood pressure to prevent cerebral vessels diseases as the most frequent causes of stroke and dementia.

A new small and cost-effective device, recently developed in our group, uses a novel wireless technology for non- invasive CAP estimation without special clinical training. Referred device was calibrated against invasive CAP measurements in the hemodynamic room. Based on European Society Cardiology guidelines [1], its alternative validation was performed by comparison with another clinical device as the reference standard (Sphygmocor), and calibration of both devices with the same brachial blood pressure (BP), to assess inter-device concordance.

The measurements were performed in Hospital Senhora da Oliveira de Guimarães, Portugal, in a sample of 68 adults, between 20 and 88 years old with a gender distribution of 46% female and 54% male, over a following range of BP: estimated central SBP was ≤ 100 mmHg (7% of readings), ≥ 140 mmHg (13% of readings), and ≥ 160 mmHg (3% of readings); estimated central DBP was ≤ 60 mmHg (1% of readings), ≥ 85 mmHg (31% of readings), and ≥ 100 mmHg (3% of readings).
From the analysis of obtained results was concluded that CAP measured by novel device fulfill the proposed pass criteria [1]: data for mean difference between devices was 0.93 mmHg (< 5 mmHg) and a standard deviation of the difference of 7,9 mmHg (< 8 mmHg). From another side, the present CAP measurements may contribute to the database of Guimarães/Vizela study on determining blood pressure in a Portuguese cohort [2].

### P.61 Perforator arteries identification: comparison of ultrasound doppler technology and infrared thermography

**Dr. Valentina Vassilenko**[1,2], Anna Poplavska[1], Edivaldo Junior[1,4], Dr. Diogo Casal[3,4]
[1]Nova School of Science and Technology, Nova University of Lisbon, Portugal, [2]Iberian Network on Arterial Structure, Central Hemodynamics and Neurocognition, Portugal, [3]Plastic and Reconstructive Surgery Department and Burn Unit, Centro Hospitalar de Lisboa Central, Lisbon, Portugal, [4]Anatomy Department, Nova Medical School-Nova University of Lisbon, Lisbon, Portugal
**Recently** an extensive clinical experience on flap surgery has confirmed that its success depends on correct identifying vital perforator vessels [1]. Unfortunately, the perforator vessels frequently have a variable location. So, the knowledge about perforator anatomy during preoperative planning is one of the most critical factors.

In this work the principles of two non-invasive technologies with the capability to localize the cutaneous perforators are described and analysed: i) ultrasound technology realized in hand-held nondirectional Doppler flowmeter, which is widely used in most hospitals and is an essential tool where a rapid analysis of the vascular status of a patient is routine; ii) Infrared Thermography (IRT) as an imaging technique that can provide indirect and real-time information on skin perfusion by measuring body surface temperature.
Both technics were applied in this work for the identification of forearm cutaneous perforator vessels (CPVs). The reflection of sound waves, predominantly from intravascular blood flow of the forearm, was registered by a hand-held BT-200 V® Vascular Doppler pan. The infrared images were obtained by two cameras: FLIR® E6 with temperature sensitivity < 0.06 °C and (320 × 240)-pixel display resolution, and Thermal Expert with sensitivity < 0.05 °C and array format 640 × 480 (Super High Resolution). Perforator mapping of the forearm area ware compared for accuracy, timing, and the operator's skills.
Obtained results show that IRT images provide valuable real-time information on the hemodynamic quality of perforators and their accurate location. Its potential to reveal underlying perforator vessels may also be used for postoperative monitoring of flap perfusion [2].

### P.62 Correlation of coronary artery calcium- and different cardiovascular risk score-based methods for the estimation of vascular age

**Dr. Milan Vecsey-Nagy**[1], Dr. János Nemcsik
[1]Heart and Vascular Center of Semmelweis University, Budapest, Hungary
**Abstract**

**Background**: A number of methods are available for the calculation of vascular age, including those derived from the Framingham Risk Score (FRS) and Systematic Coronary Risk Evaluation (SCORE). Recently, coronary artery calcium score (CACS) was proposed as a means of assessing biological age. Our aim was to compare these approaches for the assessment of vascular age.
**Methods**: 241consecutive patients were enrolled who underwent coronary CT angiography due to suspected coronary artery. CACS was calculated using the Agatston method. FRS-, SCORE-, and CACS-derived vascular age were defined according to previously described methods.
**Results**: The mean age of our patient cohort was 57.4 ± 11.2. The median vascular age based on FRS, SCORE, and CACS were 68.0 (55.0–82.0), 63.0 (53.0–75.0), and 47.1 (39.1–72.3) years, respectively, and all comparison combinations proved to differ significantly among the 3 methods (p < 0.001). FRS- and SCORE-derived biological age showed strong correlation (r = 0.84), while vascular age based on CACS moderately correlated with FRS and SCORE (r = 0.50, r = 0.52; respectively). Based on FRS, SCORE, and CACS 83.4%, 93.8%, and 42.3% of the subjects had increased vascular age compared with chronological age (FRS +, SCORE +, CACS +), and 53.2% of the FRS + (107/201) and 57.1% of the SCORE + (129/226) groups were classified as CACS-.
**Conclusions**: CACS can be conveniently used to calculate vascular age, however, it demonstrates a tendency of underestimation as compared to traditional risk equations. Prospective studies are warranted to further evaluate the contribution of CACS-based vascular age calculations to coronary risk assessment.

### P.63 Carotid artery reactivity to predict cardiovascular events in abdominal aortic aneurysm patients: Preliminary results

**Msc. Jenske JM Vermeulen**[1,2], PhD Suzanne Holewijn[1], MD PhD, Prof Michel MPJ Reijnen[1,3], PhD, Prof Dick TH Thijssen[2]
[1]Department of surgery, Rijnstate, Arnhem, Netherlands, [2]Department of Physiology, Radboud Institute for Health Sciences, Radboud University Medical Centre, Nijmegen, Netherlands, [3]Multimodality Medical Imaging Group, Techmed Centre, University of Twente, Enschede, Netherlands
**Background:** The SMART risk score calculates the risk to develop major cardiovascular events (MACE) in cardiovascular disease patients. The efficacy of this tool for abdominal aortic aneurysm (AAA) patients is unknown, while they often develop MACE. MACE is strongly linked to vascular health, which can be measured with the carotid artery reactivity (CAR)-test. Therefore, this study investigates the predictive capacity of the CAR-test for the occurrence of MACE in AAA patients compared to the SMART risk score.

**Method:** In this study, 167 patients who are under surveillance for AAA were included. All patients underwent the CAR- test to assess carotid artery diameter changes to sympathetic stimulation. Follow-up data was obtained including aneurysm progression and MACE. MACE was defined as myocardial infarction, transient ischemic attack, angina pectoris, atrial fibrillation de novo and aortic valve stenosis.
**Results:** This interim analysis had a median follow-up of 44 [29–79] weeks. Nine patients (5.4%) experienced ≥ 1 MACE, five patients (3.0%) died and 144 patients (86.0%) experienced no event during follow up. SMART risk score was highest in patients who died (60.9%, [46.8–92.3]) and in the MACE group (48.7%, [40.6–75.0]). The MACE group showed the largest dilation during CAR-test (4.5%, [1.7–5.4]) and AAA progression (6.2 mm/year, [3.0–15.6]).
**Conclusion:** In conclusion, these preliminary results suggest a relation between the CAR-test and the development of MACE in AAA patients. Patients who developed MACE showed an average dilation of the carotid artery in contrast to literature. Completion of this study should determine whether and to what extent this is a predictor for MACE.

### P.64 New method to estimate central systolic blood pressure from peripheral pressure

**Chemla D**[1], Millasseau S[2], Hamzaoui O[1], Teboul J[1], Monnet X[1], Michard F[3], Jozwiak M[4]
[1]Medical Faculty Paris-Saclay, [2]Pulse Wave Consulting, [3]MiCo, [4]APHP Cochin-Paris University
**Objective:** Cardiovascular complications may be more closely related to central than peripheral blood pressure (BP). The noninvasive estimation of central systolic BP (cSBP) is increasingly performed using new devices based on various pulse acquisition techniques and mathematical analyses. These devices are calibrated assuming unchanged mean (MBP) and diastolic (DBP) BP from aorta to peripheral artery, an assumption which is evidence-based. We tested the accuracy and precision of a new empirical formula for the Direct Central Blood Pressure estimation of cSBP (DCBP) using MBP and DBP only.

**Methods and Results:** First, we performed a post-hoc analysis of our prospective high-fidelity pressure database (n = 139, age 49 ±

12 years, 78% males). The measured aortic CSBP was 146.0 ± 31.1 mmHg. The error between aortic DCBP and aortic cSBP was − 0.9 ± 7.4 mmHg, and there was no bias across the cSBP range (82.5–204.0 mmHg). Second, we retrospectively analyzed 64 patients from the only two independent studies of the literature in whom high-fidelity pressures were simultaneously obtained in the aorta and brachial artery. The weighed mean error between brachial DCBP and aortic cSBP was 1.1 mmHg. Finally, 30 intensive care unit patients equipped with radial intra-arterial fluid-filled catheter were prospectively studied. The cSBP (115.7 ± 18.2mmHg) was obtained by carotid tonometry. The error between radial DCBP and carotid cSBP was − 0.4 ± 5.8 mmHg and there was no bias across the range.

**Conclusion:** Our study shows that cSBP could be reliably estimated from peripheral MBP and DBP without the need for any supplementary device or waveform recording, providing MBP and DBP measurement errors are minimized.

### P.65

### The effect of FluoRoquinolones on Aortic Growth, aortic stIffness and wave refLEctionS: Rationale and Design of the FRAGILES Trial

Vasiliki Gardikioti[1], Christos Georgakopoulos[1], Dimitris Iliopoulos[2], Dimitrios Terentes-Printzios[1], Eirini Solomou[1], Costas Tsioufis[1], Charalambos Vlachopoulos[1]

[1]First Department of Cardiology, Hippokration Hospital, Medical School, National and Kapodistrian University of Athens, Athens, Greece.
[2]Laboratory of Experimental Surgery and Surgical Research, Medical School, National and Kapodistrian University of Athens, Athens, Greece.

**Purpose/background/objectives:** The recently established association of quinolone use with the formation and rupture of aortic aneurysms is critical not only due to the widespread use of quinolones but also due to the severity of the induced disease. Arterial biomarkers are established predictors of cardiovascular events. The present study is designed to investigate for the first time the effect of quinolones on arterial stiffness and aortic size.

**Methods/Design:** This will be a randomized, open-label, active-comparator, assessor-blinded clinical trial of 2 parallel groups of short-term (<15 days) antibiotic therapy involving quinolones or an alternative to quinolones antibiotic agent (2 groups of 70 people each, with a patient allocation ratio of 1: 1). The study will involve individuals > 40 years of age with an indication for quinolone due to localized infection, but not sepsis, or planned procedure/surgery. The follow-up will last 6 months. The primary endpoint of the study is the estimation of the mean difference in pulse wave velocity (PWV) between the 2 groups from baseline (time 0) at 2 months after initial administration of the therapy. Secondary endpoints are PWV values at 6 months, augmentation index, central pressures (systolic, diastolic, pulse pressure) and echocardiographically-assessed aortic root diameter, as well as any adverse event (including aortic dissection and aortic rupture) at 2 and 6 months after initial treatment.

**Discussion:** To our knowledge, FRAGILES will be the first study to provide insight into the possible effects of quinolones on the aortic function. It will also investigate the possible value of arterial biomarkers on risk assessment for possible aortic dilation in this population.

**Author Index**

**A**
A Harrington P.33
Abbaoui, Y P.1
Abi-Nasr, I P.37
Adamopoulos, D 2.5
Adams, N P.2
Adapa, S P.9
Agharazii, M 1.3, P.1, P.17, P.36, P.39
Aguiar, R P.31, P.32
Aizawa, K P.3
Akamine, E. H. 2.3
AL Shezawi, O P.4
Al-Khairulla, H P.22
Alves, M 3.10, P.51, P.52, P.53
Amar, L P.44
Amaral, V 3.9
Anderson, S. G P.21
Anyfanti, P P.5
Arathy, R P.49
Aringhieri, G P.44
Armenia, S P.30, P.43
Avolio, A P.10, P.19, P.26, P.57
Avril, S P.40
Ayden, A P.38
Aznaouridis, K 1.7
**Å**
Åström Malm, Ida P.6
**B**
Badhwar, S 7.5
Barcelos, A P.31, P.32
Barros, P. R. 2.3
Batta, D P.34
Baynard, T P.56
Bellien, J P.50
Ben Moussa, Olfa P.40
Benbia, A P.37
Benetos, A 2.2
Beutel, F 7.1
Bianchini, E 2.4, P.30
Bidar, E 7.2
Bikia, V 2.5, 7.3, P.36
Blomstrand, P P.6
Boban, M P.42
Bortolotto, L 3.10, P.8. P.51, P.52, P.53
Boutouyrie, P 3.4, P.16, P.44
Brandt, D P.35
Brassard, P P.39
Brum, T P.11
Bruno, R. M 3.4,P.16,P.30, P.43, P.44, P.59
Büschges, J. C 1.2
Butlin, Mark P.10, P.26, P.57
Buzzelli, Andrea P.30
**C**
Cardoso, Filipa P.60
Carretta, R P.19
Casal, D P.61
Casanova, F P.3
Chandran, D 7.5, P.55
Chang, A P.9
Charlton, P 2.4
Chaturvedi, N 1.1
Chemla, D P.64
Cheung, M P.33
Chiappino, D 1.6
Chowienczyk, P 1.4
Christopoulou, G 3.3
Ciolac, Emmanuel Gomes 3.9
Climie, R 2.4, 3.6
Cockcroft, J P.4
Colhoun, H. M P.3
Cosottini, M P.43
Costa, M 3.10, P.51, P.52, P.53
Costa, T. J. 2.3
Costa-Hong, Valéria 3.10, P.8, P.51, P.52, P.53
Cox, James P.10, P.26
Cozma, Angela P.25
Cruickshank, K. 1.6
Cseprekál, O P.34
Cunha, M P.11, P.12
Cunha, P P.60
**d**
da Silva-Neto, J. A. 2.3
de Korte, C 7.4, P.48
**D**
Dániel, K P.24
Dantas, A. P. 2.3
Dbrzyn, P P.14
De Basso, R P.6
De Censi, L P.19
Debeij, G 7.2
Deepak, K. K 7.5, P.55
Delhaas, Tammo 3.7, 7.2, P.59
Della Latta, D 1.6
Di Franco, G P.30
Dima, I 1.7, 3.3
Dinnissen, D P.20
Dogan, S P.38
Douma, S P.5
Drapeau, A P.39
Duarte, D. A. 2.3
DuBose, L P.35
Duval, K P.17, P.36, P.39
**E**
Elia, S. A. 3.6
Emmanouil, E 1.7, 3.3
Engström, G 3.2
Engvall, J P.6
Eveilleau, K P.37
**F**
Fabbri, S 3.4
Fabris, B P.19
Fachim, H P.21
Faconti, L 1.4
Faita, F P.43, P.44
Farukh, B 1.4
Faulkner, J 3.1, 3.5, P.54
Fayol, A P.16
Fekkes, S P.48
Fernandes, B 3.9
Fernhall, B P.29, P.56
Filip, A P.14
Filipovský, J P.15
Fodor, A P.25
Fortier, C P.16,P.17, P.36, P.39, P.44
Francesconi, M P.30
Fraser, A 1.1
Fryer, S 3.1, 3.5, P.2, P.54
**G**
Gale, N P.4
Ganizada, B 7.2
Gardikioti, V 3.3, P.65
Garneau, C P.17
Garzon, S P.8
Gates, P. E P.3
Gavish, B P.18
Gavriilaki, E P.5
Gelžinský, J P.15
Gemignani, V P.30
Georgakopoulos, C 3.3, P.65
Ghiadoni, L P.43, P.44
Giannattasio, C P.59

Gibson, M P.21
Gimenez, L. B 3.9
Giudici, A 1.6, 3.7
Gkaliagkousi, E P.5
Glenning, J P.33
Gonçalves, I P.3
Gooding, K. M P.3
Goupil, R 1.3, P.1
Gourgouli, I 3.3
Grigoriadis, G P.56
Grillo, A P.19
Guignandon, A P.40
Guvenc T. B P.38
Gyöngyösi, H P.34
**H**
Hallab, M P.37
Hametner, B 1.3
Hamrefors, V 3.2
Hamzaoui, O P.64
Hannink, G 3.8
Hansen, H 7.4, P.48
Hartman, Y P.20
Hashimoto, J 1.5
Heald, A P.21
Heffernan, Kevin 3.1, 3.5, P.54
Hermeling, E 7.1
Holewijn, S P.63
Houillier, P 3.4
Howe, L 1.1
Huberts, W 7.2
Hudson, L P.22
Hughes, A 1.1 P.22, P.3
Hughes, T 3.1, 3.5
**I**
Iacob, M P.50
Iliopoulos, D P.65
Ioakeimidis, N 1.7
Ito, S 1.5
**J**
Jacobs, D P.22
Jamagidze, G 1.6
Jaryal, A 7.5
Jerončić, A P.42
Jiménez-Altayó, F 2.3
Joannides, R P.50
Johansson, M 3.2
Jones, S 1.1
Joseph, J P.23, P.27, P.28, P.49, P.55
Jozwiak, M P.64
Junior, E P.61
**K**
Kaanders, J 7.4
Kahn, F P.3
Karkhaneh Yousefi, A P.40
Kettab, H 3.4, P.16, P.44
Khir, A. W. 1.6, 3.7
Klein, M P.11, P.12
Kling, J 7.1
Kondiboyina, A P.33
Königstein, Ka 1.2
Koren, P P.42
Korompoki, E 2.1
Kőrösi, B. Z P.34
Kowalski, R P.33
Kozakova, M 1.6
Kucharska-Newton, A 3.1, 3.5
Kulin, S P.24
Kyrkou, A 2.1
**L**
Labat, C 2.2
Labrecque, L P.39
Lacolley, P 2.2
Lagrange, J 2.2
Lamarche, F 1.3, P.1
Lambrick, D P.54
László, A P.34
Lazar, A P.25
Lazaridis, A P.5
Lazaridou, E P.5
Lazaros, G 3.3
Leftheriotis, G P.37
Lemos, P P.8
Locato, G 3.9
Loonen, J 7.4
Lorthioir, A P.44
Louka, K P.26
**M**
Maas, D 3.8
Madore, F 1.3, P.1
Maessen, J 7.2
Maloberti, A P.59
Mancuso, M P.43
Manios, E 2.1
Manoj, R P.27, P.28
Marçal, I. R 3.9
Margouta, A P.5
Marôco, João Luís P.29
Martina, M. R. P.30
Maruani, G 3.4
Masson, G P.25
Matsushita, K 3.1, 3.5
Mattos, S P.11, P.12
Mawson, D. M P.3
Mayer, C. C. 2.4, 3.6
Mayer, O P.15
Mayet, J P.9
Mazeda, C P.31, P.32
McDonnell, B P.4
McNally, R 1.4
Melo, X P.29
Mendes, N P.8
Mesquita B. J P.31, P.32
Meyer, M 3.1, 3.5 P.2, P.54
Michard, F P.64
Michel, J.B. P.40
Miklós, Z P.24
Miliou, A 3.3
Millasseau, S P.64
Monnet, X P.64
Moretti, F P.19
Morizzo, C 1.6
Mudnic, I P.42
Munnery, M P.4
Mynard, J P.33
**N**
Nabeel, P M P.23, P.27, P.28, P.49, P.55
Nadeau-Fredette, A 1.3, P.1
Nandi, M 2.4
Narang, R 7.5
Natour, E7.2
Negrean, V P.25
Nemcsik, J P.34, P.62
Nemcsik-Bencze, Z P.34
Neuhauser, H 1.2
Neves, M P.11, P.12
Ngomane, A.Y 3.9
Nicholls, D P.22
Nikolaidou, B P.5
Nilsson, J P.3
Nilsson, P. M 3.2
Ntaios, G 2.1
Nuckols, V P.35

**O**
Obeid, Hasan P.17, P.36, P.37
Oliveira-da-Silva, R. D. N 2.3
Ollier, B P.21
Orasan, O P.25
O'Rourke, M P.26
Oztemiz, E P.38
**P**
Pagan L, P 3.5
Pagoulatou, S 2.5, 7.3
Palombo, C 1.6, P.3
Panayiotou, A. G. 3.6
Papamichael, C 2.1
Parati, G. P.19
PARE, M P.17,P.36, P.39
Parikh, S 7.2
Park, C 1.1, 3.6
Parker, K. H. P.3
Patel, C 7.5
Paterson, C 3.1, 3.5, P.2
Patsatsi, A P.5
Peng, Y P.21
Penno, G 1.6
Perrini, P P.43
Peterson, C P.54
Petit, C P.40
Pierce, G P.35
Pinto, M P.29
Pirounaki, M 3.3
Piskin, S P.41
Podrug, M P.42
Poles, J 3.1, P.2
Poli, F P. 43, P.44
Pölz, A P.45
Pomeroy, A P.46
Poplavska, A P.61
Posokhov, I P.47
Protogerou, A 2.1
Pruijssen, J 7.4, P.48
**Q**
Qasem, Ahmad P.10
**R**
Raj, K. V P.23, P.27, P.28, P.49, P.55
Rapala, A P.22
Ratneswaren, A P.9
Reavette, R P.9
Reddy, D P.9
Reesink, K 7.2
Reesink, K.D. 3.7, P.59
Regnault, V 2.2
Reijnen, Mi 3.8, P.63
Riksen, Niels 3.8
Roca, Frédéric P.50
Rodrigues, B. G. P.25
Rodrigues, Sara 3.10, P.51, P.52, P.53
Rojas, J P.54
Rovas, G 2.5, 7.3
Rovina, M P.19
Rowland, E P.9
Roy, M.A. P.39
**S**
Salvi, L P.19
Salvi, P P.19
Samara, S 2.1
Santa-Clara, H P.29
Santillan, M P.35
Santos-Eichler, R.A. 2.3
Sarganas, G 1.2
Scalise, F P.19
Schaffrath Rosario, A 1.2
Schalkwijk, C. G. P.59
Schienkiewitz, A 1.2
Schmidt-Trucksäss, A 1.2, 2.4
Schurgers, Leon 7.2
Segers, P 2.4, 7.3
Seidlerová, J P.15
Sen, S P.55
Serrano, A P.60
Sherman, S P.56
Shirbani, F P.57
Shore, A. C. P.3
Silva, L P.12
Silva, S P.31, P.32
Sitar, A P.25
Smolich, J P.33
Solomou, E 3.3, P.65
Sorropago, A P.19
Sorropago, G P.19
Souza, F. B. 3.9
Spronck, Bart 3.7, 7.2, P.59
Stamatelopoulos, K 2.1
Stanford, K P.46
Stergiopulos, Nikolaos 2.5, 7.3, P.36
Stone, K 3.1, 3.5, P.2, P.54
Stoner, L 3.1, 3.5, P.2, P.46, P.54
Strain, D. W. P.3
Stroud, A P.35
**Š**
Šunjić, B P.42
**T**
Taddei, S P.44
Tagawa, K 1.5
Tan, I P.10, P.19, P.26 P.57
Tanaka, H 3.1, 3.5
Taylor, H 1.1
Tayyarah, M P.37
Teboul, J.L. P.64
Ten Cate, H 3.8
Terentes-Printzios, D 1.7, 2.4, 3.3, P.65
Thijssen, D P.20,P.63
Thill, Caroline P.50
Thomas, A 2.2
Tislér, A P.34
Tomiyama, H P.7
Torunova, A P.58
Tostes, R. C. 2.3
Toupance, S 2.2
Tousoulis, D 1.7
Triantafyllou, Areti 3.6, P.5
Tsioufis, Konstantinos 3.3, P.65
**v**
van der Bruggen, M P.59
van der Heijden, A 7.1
**V**
Van der Vijver-Coppen, R 3.8
Van Hoof, C 7.1
Vassilenko, V P.60, P.61
Vecsey-Nagy, M P.62
Vemmos, Konstantinos 2.1
Vemmou, A 2.1
Verardino, R 3.10, P.51, P.52, P.53
Vermeulen, JP.63
Viner, R P.22
Vitali, S P.44
Vlachopoulos, C 1.7, 3.3, P.65
**W**
Warlé, M 3.8
Wassertheurer, S 1.3
Watkeys, L P.4
Weinberg, P P.9
Wenmakers, A 7.4
Westerhof, B 1.5

Wilbers, J 7.4
Willems, L 3.8
**X**
Xydis, P 3.3
**Y**
Yiannaki, E P.5
**Z**
Zanini, G 3.9
Zax, C 7.1
Zeebregts, C 3.8
Zieff, G 3.1, 3.5, P.2, P.54
